# Aldiminium and 1,2,3-triazolium dithiocarboxylate zwitterions derived from cyclic (alkyl)(amino) and mesoionic carbenes

**DOI:** 10.3762/bjoc.19.145

**Published:** 2023-12-20

**Authors:** Nedra Touj, François Mazars, Guillermo Zaragoza, Lionel Delaude

**Affiliations:** 1 Laboratory of Catalysis, MolSys Research Unit, Université de Liège, Institut de chimie organique (B6a), Allée du six août 13, 4000 Liège, Belgium; 2 Unidad de Difracción de Rayos X, RIAIDT, Universidade de Santiago de Compostella, Edificio CACTUS, Campus Vida, 15782 Santiago de Compostela, Spain

**Keywords:** betaines, carbenes, ligand effects, nitrogen heterocycles, zwitterions

## Abstract

The synthesis of zwitterionic dithiocarboxylate adducts was achieved by deprotonating various aldiminium or 1,2,3-triazolium salts with a strong base, followed by the nucleophilic addition of the in situ-generated cyclic (alkyl)(amino) or mesoionic carbenes (CAACs or MICs) onto carbon disulfide. Nine novel compounds were isolated and fully characterized by ^1^H and ^13^C NMR, FTIR, and HRMS techniques. Moreover, the molecular structures of two CAAC·CS_2_ and two MIC·CS_2_ betaines were determined by X-ray diffraction analysis. The analytical data recorded for all these adducts were compared with those reported previously for related NHC·CS_2_ betaines derived from imidazolinium or (benz)imidazolium salts. Due to the absence of electronic communication between the CS_2_ unit and the orthogonal heterocycle, all the CAAC·CS_2_, MIC·CS_2_, and NHC·CS_2_ zwitterions displayed similar electronic properties and featured the same bite angle. Yet, their steric properties are liable to ample modifications by varying the exact nature of their cationic heterocycle and its substituents.

## Introduction

**Figure 1:**
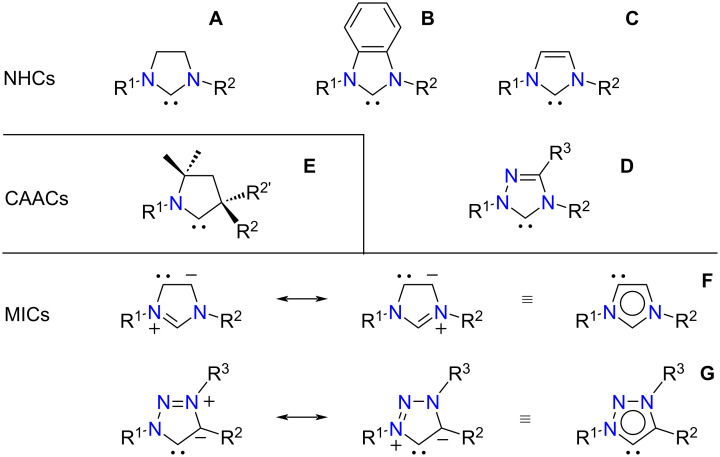
Various types of stable singlet carbenes and their acronyms.

Following the seminal discovery from the group of Arduengo, who isolated and fully characterized 1,3-di(1-adamantyl)imidazol-2-ylidene in 1991 [1], stable divalent carbon species have evolved from fleeting intermediates to ubiquitous catalysts, ligands, and reagents in just three decades (Figure 1) [2]. In particular, cyclic diaminocarbenes based on the imidazoline, benzimidazole, or imidazole ring system (**A**–**C**) have led to a myriad of applications in organometallic chemistry, homogeneous catalysis, and materials science, to name just a few [3–5]. Due to their weaker basicity and greater modularity, the related 1,2,4-triazol-5-ylidene derivatives **D** have been mainly employed in organocatalysis [6]. Besides these four types of N-heterocyclic carbenes (NHCs), other families of cyclic compounds have been actively pursued to further expand the diversity of singlet carbenes available to the chemist [7]. Among them, the cyclic (alkyl)(amino)carbenes (CAACs, **E**) introduced by Bertrand et al. in 2005 [8] have attracted a great deal of attention, thanks to their remarkable nucleophilic (σ-donating) and electrophilic (π-accepting) properties, which allow them to activate a variety of small molecules and to bind strongly to metal centers, thereby affording very robust catalysts [9–12].

Another category of stable carbenes that has emerged in the new millennium is made of mesoionic compounds, for which no reasonable canonical resonance form can be drawn in the absence of charges (Figure 1) [13–16]. Crabtree and co-workers first reported the abnormal binding of an imidazolium salt to an iridium hydride at the C4 carbon atom instead of C2 in 2001 [17–18]. Since then, many other metal complexes bearing imidazol-4-ylidene ligands (**F**) have been reported [7,19]. These mesoionic carbenes (MICs), together with their pyrazolin-4-ylidene [20] and 1,2-isoxazol-4-ylidene cousins [21], are the strongest donors among the various types of carbene ligands known thus far [22]. A distinct class of mesoionic or abnormal carbenes based on the 1,2,3-triazole ring system (**G**) was first investigated by Albrecht et al. in 2008 [23]. Because the heterocyclic precursors needed to prepare 1,2,3-triazol-5-ylidenes are readily available through the [3 + 2] cycloaddition of an azide and an alkyne, these compounds are currently the most popular MICs for catalytic and other applications [24–28].

**Figure 2:**
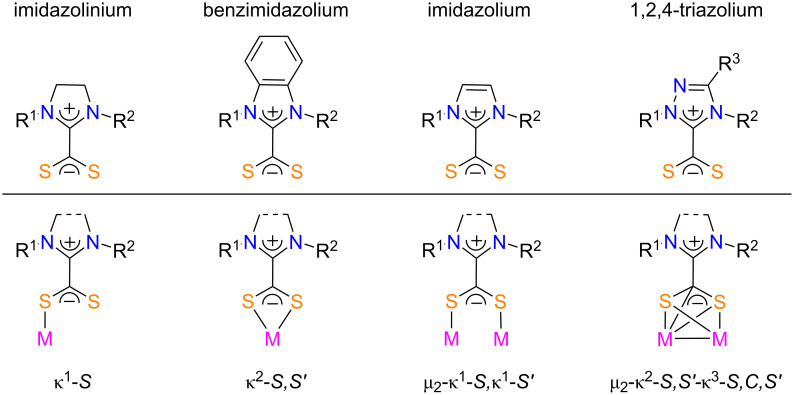
Various types of NHC·CS_2_ zwitterions and their coordination modes.

N-Heterocyclic carbenes and their enetetramine dimers readily add to the central carbon atom of allenes and heteroallenes X=C=Y (X, Y = CR_2_, NR, O, S) to afford zwitterionic adducts [29]. In particular, their reaction with carbon disulfide affords stable azolium-2-dithiocarboxylate zwitterions (Figure 2) [30–43]. These 1,1-dithiolate inner salts strongly bind main group elements and transition metals through various coordination modes. Indeed, we and others have already reported the synthesis of diverse metallic complexes featuring monodentate [44–45], chelating bidentate [46–55], or bridging bidentate NHC·CS_2_ ligands [45,51–52]. Small bimetallic clusters [51–52,56], coordination polymers [57], self-assembled monolayers [58], and nanoparticles [45] based on these zwitterions were also prepared, while a few reports disclosed the formation of polynuclear clusters, in which the dithiocarboxylate unit underwent further chemical transformations [59–61].

**Scheme 1:**
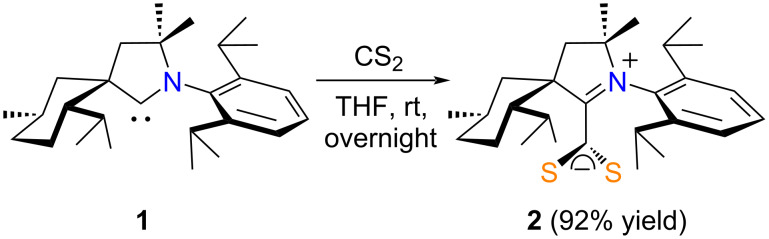
Synthesis of CAAC·CS_2_ zwitterion **2** from its free carbene parent **1**.

To the best of our knowledge, 1,2,3-triazolium-5-dithiocarboxylate species are hitherto unknown in the literature, and only a single report described the preparation of a CAAC·CS_2_ zwitterion. Thus, in 2009 Bertrand et al. obtained the betaine **2** by reacting the free carbene **1** with a slight excess of CS_2_ in THF at room temperature (Scheme 1) [62]. The starting material that featured a bulky and rigid spirocyclic alkyl group derived from (−)-menthone was obtained in a separate step by deprotonating the corresponding aldiminium triflate with lithium diisopropylamide (LDA) at −78 °C [8].

Herein, we disclose the synthesis of three CAAC·CS_2_ and six MIC·CS_2_ inner salts from the corresponding aldiminium or 1,2,3-triazolium salts and carbon disulfide. All these adducts were fully characterized by using various analytical techniques and their structural properties compared to those displayed by known, related imidazolinium and (benz)imidazolium-2-dithiocarboxylate betaines.

## Results and Discussion

Currently, the most general strategy to prepare NHC·CS_2_ zwitterions relies on the deprotonation of an azolium salt with a strong base, typically potassium *tert*-butoxide or potassium bis(trimethylsilyl)amide (also known as potassium hexamethyldisilazide, KHMDS) followed by the addition of carbon disulfide either in one pot or after the isolation of the free carbene [39,41–42,58]. Hence, we decided to probe the feasibility of this approach for the synthesis of CAAC·CS_2_ and MIC·CS_2_ betaines from readily available aldiminium or 1,2,3-triazolium salts.

### Synthesis of CAAC·CS_2_ zwitterions

**Scheme 2:**
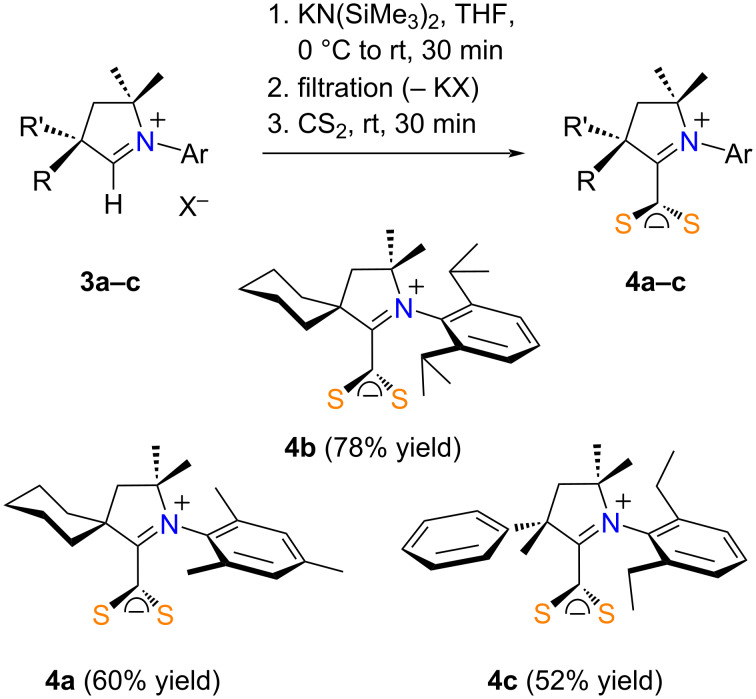
Synthesis of CAAC·CS_2_ zwitterions **4a**–**c** with KN(SiMe_3_)_2_.

To begin our study, we investigated the synthesis of CAAC·CS_2_ zwitterions starting from three commercially available aldiminium salts **3a**–**c** (Scheme 2). These reagents were suspended in THF and cooled to 0 °C before a 1 M solution of KN(SiMe_3_)_2_ in THF was slowly added to release the free carbenes. Of note, compound **3b** was purchased as a hydrogen dichloride salt and required the use of a double amount of base. The suspensions were brought back to room temperature and allowed to settle down to ease the filtration of the inorganic byproducts (KCl or KBF_4_), along with any unreacted starting materials. Adding an excess of CS_2_ to the free carbene solutions led to the instantaneous formation of the desired zwitterionic adducts, as evidenced by the appearance of an intense orange-red color. The solvent was removed and the residues were brought back to air, washed with *n*-pentane, and dried under high vacuum to afford pseudo-cross-conjugated mesomeric betaines **4a**–**c** in high yields (ca. 80%). NMR analysis showed that compounds **4a** and **4c** required further purification. Thus, they were recrystallized from acetonitrile, which led to a non-negligible loss of materials, thereby leading to final yields in the 50–60% range.

### Synthesis of MIC·CS_2_ zwitterions

**Scheme 3:**
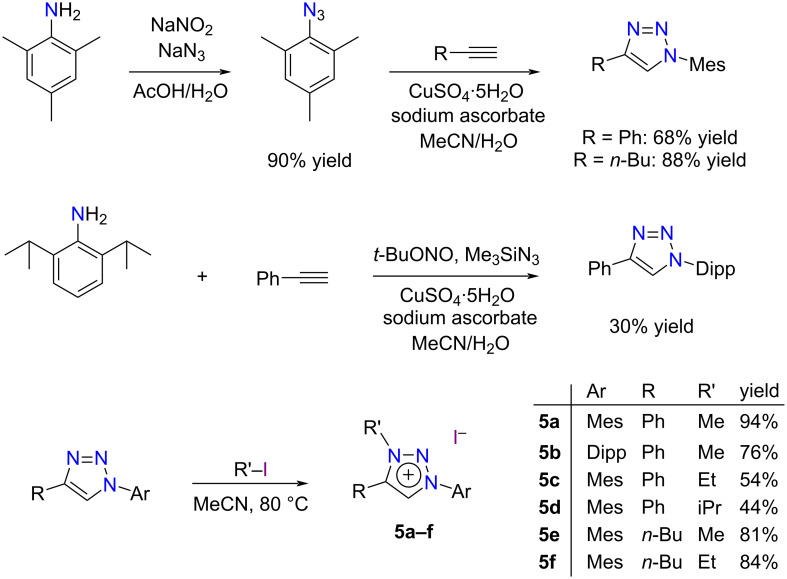
Synthesis of 1,2,3-triazolium iodides **5a**–**f**.

The synthesis of 1,4-disubstituted-1,2,3-triazole derivatives is readily achieved via the copper(I)-catalyzed [3 + 2] cycloaddition of an azide and a terminal alkyne (CuAAC) [63–65]. A further alkylation of the N3 position with an alkyl halide is an equally straightforward procedure that ultimately affords a large assortment of MIC precursors [24–28]. By analogy with the archetypical NHCs bearing mesityl (Mes) or 2,6-diisopropylphenyl (Dipp) substituents on their nitrogen atoms, we have prepared three triazole derivatives with mixed Mes/Ph, Mes/Bu, or Dipp/Ph substituents on N1 and C4, respectively (Scheme 3). The active catalytic species for the CuAAC reaction were generated by reducing copper(II) sulfate with sodium ascorbate according to literature procedures [66–67]. 2-Azido-1,3,5-trimethylbenzene (mesityl azide) was easily synthesized in a distinct, preliminary step through the Sandmeyer reaction of mesitylamine with sodium nitrite and acetic acid followed by a substitution of the intermediate diazonium salt with sodium azide [68]. All our attempts to prepare 2-azido-1,3-diisopropylbenzene along the same lines failed. Nevertheless, its in situ formation in the presence of phenylacetylene led to the desired cycloadduct, although in a modest 30% yield [69]. The subsequent alkylation of N3 with methyl, ethyl, or isopropyl iodide afforded triazolium salts **5a**–**f** in satisfactory to excellent yields. It should be pointed out that compounds **5c**–**f** had never been described before (see the Supporting Information File 1 for experimental details).

**Scheme 4:**
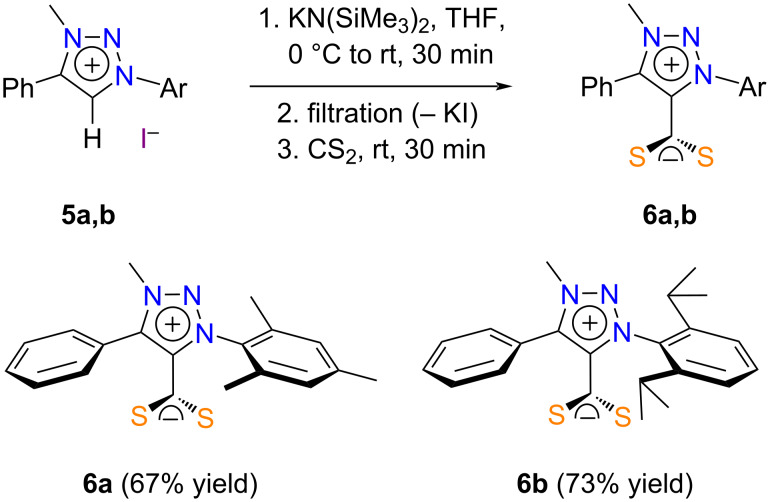
Synthesis of MIC·CS_2_ zwitterions **6a** and **6b** with KN(SiMe_3_)_2_.

We initially carried out the synthesis of MIC·CS_2_ zwitterions from the set of six triazolium iodides in our hands according to the experimental procedure described above for CAAC·CS_2_ betaines (cf. Scheme 2). Thus, the salts **5a**–**f** were deprotonated with KN(SiMe_3_)_2_ (1.2 equiv) in THF at 0 °C. The potassium iodide byproduct was filtered off and carbon disulfide (3.3 equiv) was added to the carbene solution leading to an immediate color change. After 30 min at room temperature, the solvent was evaporated under vacuum. The residue was washed with petroleum ether and dried under high vacuum. ^1^H NMR analysis revealed that a significant amount of starting material was still present in most cases. Moreover, unidentified byproducts were also detected in some instances. Products **6a** and **6b** could be isolated in pure form and satisfactory yields upon recrystallization from acetonitrile (Scheme 4). However, all our attempts to purify compounds **6c**–**f** by recrystallization or column chromatography remained unsuccessful.

**Scheme 5:**
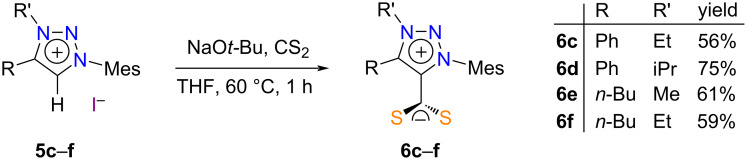
Synthesis of MIC·CS_2_ zwitterions **6c**–**f** with NaO*t*-Bu.

Several studies have shown that metal alkoxides, such as potassium *tert*-butoxide (p*K*_a_ = 22), were basic enough to deprotonate 1,2,3-triazolium salts (p*K*_a_ ≈ 22–23) depending on the nature of their aliphatic or aromatic substituents, and that the use of the stronger base KN(SiMe_3_)_2_ (p*K*_a_ = 26) was not always mandatory [25,69–70]. Grubbs, Bertrand et al. also noticed that treating 1-benzyl-3-methyl-4-phenyl-1*H*-1,2,3-triazolium iodide with KO*t*-Bu did not afford the desired MIC but led to a debenzylated triazole instead [71]. Based on these observations, we decided to revise our experimental procedure for the synthesis of MIC·CS_2_ zwitterions by using a mixture of NaO*t*-Bu and CS_2_ from the onset of the reaction in THF at 60 °C. These two reagents were added in large excess to compensate for the possible formation of sodium *O*-*tert*-butyl xanthate [72–74]. We reasoned that these conditions should favor a quantitative deprotonation of the starting triazolium salts and the concomitant trapping of the free carbenes by CS_2_ prior to their potential decomposition. Gratifyingly, this method allowed us to isolate the cross-conjugated mesomeric betaines **6c–f** in good yields (Scheme 5). Clean NMR spectra were recorded in all cases, although elemental analysis revealed that the products were not entirely homogeneous. This might be due to the presence of inorganic impurities. Yet, we did not try to purify them any further.

Before closing this section, it should be stressed that we were not able to isolate any dithiocarboxylate betaines when aldiminium salt **3b** or triazolium salt **5a** were treated with cesium carbonate and carbon disulfide in acetonitrile in the presence of air and moisture, followed by an aqueous work-up. These results sharply contrasted with those obtained previously with a range of imidazolinium, benzimidazolium, or imidazolium salts, which were successfully converted into NHC·CS_2_ zwitterions under mild aerobic conditions [75]. Yet, the greater proton affinity and the higher basicity of CAACs and MICs vs NHCs [25,69–71] did not prevent the formation of the desired adducts using Cs_2_CO_3_ instead of KN(SiMe_3_)_2_ or NaO*t*-Bu, as evidenced by the appearance of revealing orange-red colors and by the emergence of a characteristic resonance for the CS_2_^−^ unit on ^13^C NMR spectroscopy (see below). We suspect that deleterious hydrophilic effects caused the subsequent decomposition of the CAAC·CS_2_ and MIC·CS_2_ zwitterions when an aqueous work-up was applied.

### Structural analysis

Several analytical techniques were employed to characterize the nine aldiminium and 1,2,3-triazolium dithiocarboxylate betaines under investigation. ^1^H NMR spectra of compounds **4a**–**c** and **6a**–**f** were rather similar to those of their precursors **3a**–**c** and **5a**–**f**, respectively, with only one less resonance due to the replacement of their acidic proton with a CS_2_ group (Table 1). Because the vanishing signal was always the most deshielded singlet in the spectra of the reagents, it was a very convenient probe to monitor the success of the deprotonation step. Concomitantly, the incorporation of CS_2_ in products **4a**–**c** and **6a**–**f** led to the emergence of an equally characteristic resonance in the ^13^C NMR spectra. Indeed, with values higher than 220 ppm, the chemical shift of a dithiocarboxylate unit is located in a spectral region where it can hardly be mistaken for anything else.

**Table 1:** Characteristic ^1^H and ^13^C NMR chemical shifts recorded for CAAC·CS_2_ and MIC·CS_2_ zwitterions **4a**–**c** and **6a**–**f** and their precursors **3a**–**c** and **5a**–**f** (data recorded in CDCl_3_ at 298 K).

Reagent	δ NC*H* (ppm)	δ N*C*H (ppm)	Product	δ N*C*CS_2_ (ppm)	δ CS_2_ (ppm)

**3a**	8.78	191.3	**4a**	188.7^a^	230.5^a^
**3b**	10.7	193.0	**4b**	189.0^a^	227.7^a^
**3c**	9.53	189.7	**4c**	188.2^a^	228.8^a^
**5a**	9.03	130.3	**6a**	150.5^a^	225.6^a^
**5b**	8.77	130.6	**6b**	150.5	225.0
**5c**	8.90	130.7	**6c**	150.2	225.0
**5d**	9.43	130.0	**6d**	150.3	224.9
**5e**	8.46	130.3	**6e**	150.3	225.5
**5f**	8.55	130.5	**6f**	150.4^a^	226.3^a^

^a^Data recorded in CD_2_Cl_2_.

On average, the δ CS_2_ value recorded for aldiminium inner salts **4a**–**c** (229 ppm) was slightly higher than for triazolium derivatives **6a**–**f** (225 ppm). Previously, we had reported chemical shifts in the 220–226 ppm range for a series of imidazolinium, benzimidazolium, or imidazolium-2-dithiocarboxylate zwitterions with various aliphatic or aromatic substituents on their nitrogen atoms [40,75]. Hence, the CS_2_ resonance is not significantly altered by the nature of the adjacent heterocycle, in line with a lack of electronic communication between these two moieties, as further discussed below. Contrastingly, the ^13^C NMR resonance for the carbenoid center of all the reagents and products used in this study was clearly affected by the type of heterocycle it belonged to (Table 1). The average chemical shift for C2 was 191 ppm in aldiminium salts **3a**–**c** and 189 ppm in inner salts **4a**–**c**. These values are significantly higher than those recorded for NHC·CS_2_ zwitterions based on imidazolinium (165 ppm), benzimidazolium (152 ppm), or imidazolium (149 ppm) derivatives [75], which are surrounded by two nitrogen atoms instead of one. It is noteworthy that the C2 resonance found at ca. 130 ppm in triazolium salts **5a**–**f** underwent a significant downfield shift to about 150 ppm in inner salts **6a**–**f**. This is the largest variation of chemical shift observed for C2 when replacing its acidic proton with a CS_2_ group among all the nucleophilic carbene precursors that we have investigated so far [40,75]. Yet, we do not have an explanation for it.

On IR spectroscopy, the most intense absorption in the ATR spectra of compounds **4a**–**c** and **6a**–**f** was always due to the asymmetric stretching of the CS_2_ group (Table 2). This band was observed at wavenumbers ranging from 1037 to 1050 cm^−1^, down from 1052–1080 cm^−1^ for common imidazol(in)ium-2-dithiocarboxylate inner salts bearing aliphatic or aromatic substituents on their nitrogen atoms [40]. This shift to lower energies is a likely consequence of the greater donicity of CAACs and MICs vs NHCs. Hence, the ν̃ CS_2_ values recorded on IR spectroscopy constitute a more sensitive probe than the δ CS_2_ values obtained from ^13^C NMR spectroscopy to help discriminate the various types of dithiocarboxylate adducts derived from nucleophilic carbenes. More sophisticated methods, such as the determination of the Huynh electronic parameter, should, however, be better suited to evaluate more precisely the influence of substituents on the donating ability of carbene ligands [22,76–77].

**Table 2:** IR stretching vibrations recorded for CAAC·CS_2_ and MIC·CS_2_ zwitterions **4a**–**c** and **6a**–**f** in the ATR mode.

Compound	ν̃ C=S (cm^−1^)	ν̃ C=C (cm^−1^)	ν̃ C=N (cm^−1^)

**4a**	1037	1424	1552
**4b**	1050	1446	1536
**4c**	1040	1456	1554
**6a**	1043	1482, 1456	
**6b**	1044	1465, 1440	
**6c**	1045	1481, 1448	
**6d**	1047	1480, 1448	
**6e**	1044	1454	
**6f**	1042	1455	

Apart from the asymmetric stretching vibration of the S=C–S^−^ group, another strong absorption was clearly visible in the IR spectra of CAAC·CS_2_ betaines **4a**–**c**. This second most intense band was observed around 1550 cm^−1^ (Table 2). It probably originated from the asymmetric stretching of the aldiminium group, in line with similar high intensity bands previously observed at ca. 1528 and 1477 cm^−1^, respectively, in the IR spectra of imidazolinium and imidazolium inner salts [40]. Contrastingly, no remarkable absorption was detected in the IR spectra of triazolium derivatives **6a**–**f** for the CNN or NNN motifs. Yet, in all the cases, medium bands were observed in the 1400–1500 cm^−1^ region (Table 2). These patterns, often a doublet, were tentatively assigned to skeletal vibrations involving C=C stretching of the aromatic rings, although the intervention of asymmetrical CH deformation modes (e.g., bending or scissoring) could not be excluded.

### Crystallography

**Figure 3:**
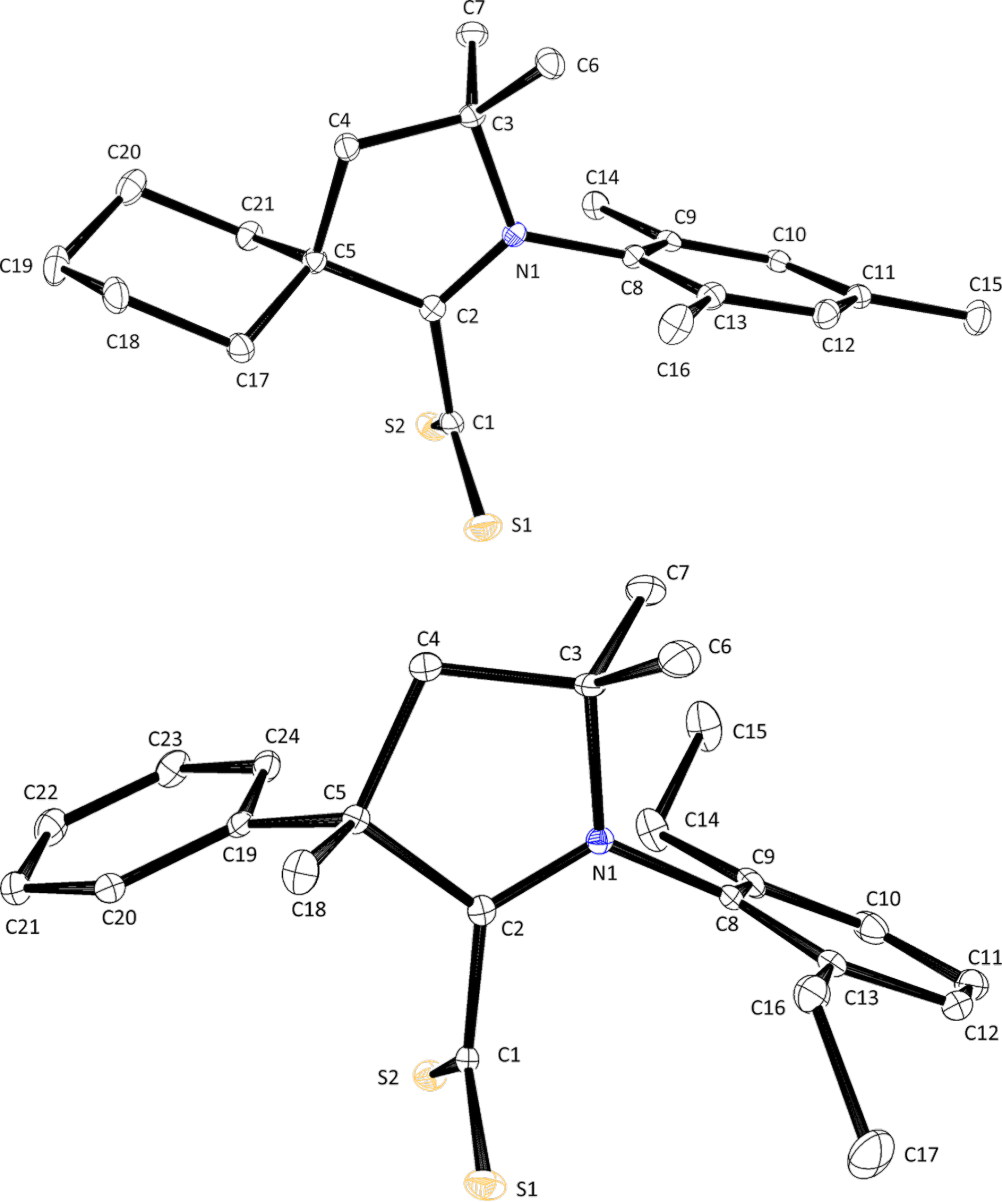
ORTEP representations of zwitterions **4a** (CAAC-Mes-Cy·CS_2_, top) and **4c** (CAAC-Die-MePh·CS_2_, bottom) with thermal ellipsoids drawn at the 50% probability level.

Crystals of CAAC·CS_2_ zwitterions **4a** and **4c** suitable for X-ray diffraction (XRD) analysis were grown by slow diffusion of cyclohexane in a THF solution at 6 °C. Their molecular structures are depicted in Figure 3. The orange-red needles of compound **4a** belonged to the trigonal 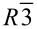 space group, while the orange plates of compound **4c** belonged to the monoclinic *P*2_1_/*c* group. Due to the asymmetry of the quaternary carbon atom adjacent to the carbene center, the latter compound crystallized as a racemic mixture.

**Figure 4:**
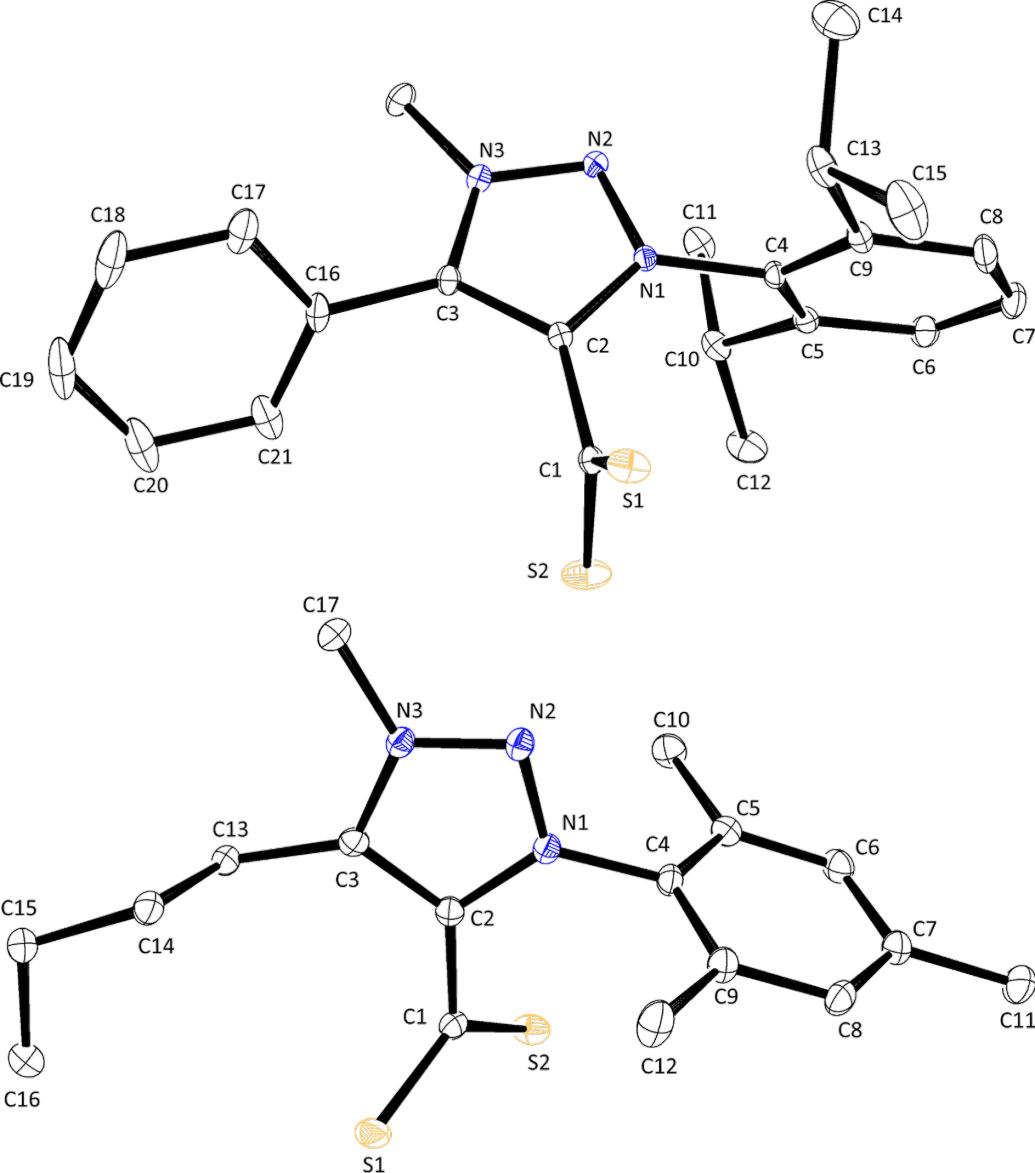
ORTEP representations of zwitterions **6b** (MIC-Dip-Ph-Me·CS_2_, top) and **6e** (MIC-Mes-Bu-Me·CS_2_, bottom) with thermal ellipsoids drawn at the 50% probability level.

Solutions of MIC·CS_2_ zwitterions **6a**–**f** in CD_2_Cl_2_ or CDCl_3_ employed for NMR analyses were layered with petroleum ether or *n*-hexane and kept at −18 °C for a few weeks. This procedure successfully afforded single crystals of products **6b** and **6e** suitable for XRD analysis (Figure 4). Orange prisms of zwitterion **6b** belonged to the monoclinic *P*2_1_/*n* space group, while the dark red-brown blocks of compound **6e** belonged to the *P*2_1_/*c* space group.

A comparison of the C1–S1 and C1–S2 distances recorded in the four crystal structures under scrutiny and those determined previously for 1,3-dimesitylimidazolium-2-dithiocarboxylate (IMes·CS_2_) and its more saturated analogue SIMes·CS_2_ showed that the negative charge of the CS_2_^−^ unit was uniformly delocalized between the two sulfur atoms (Table 3). Moreover, the average length of 1.67 Å recorded for all the C–S bonds matched the typical distance compiled for double rather than single CS bonds (1.67 vs 1.75 Å) [78]. At (130 ± 1)°, the S1–C1–S2 bite angle of the various 1,1-dithiolate ligands was also remarkably constant. With values comprised between 87 and 126°, the S1–C1–C2–N1 dihedral angle between the CS_2_^−^ moiety and the heterocyclic ring was more fluctuant, but a nearly orthogonal rather than planar disposition was maintained in all cases. Through-space attractive coulombic interactions between the opposite charges were held responsible for this orientation [79]. In addition, the presence of bulky aryl substituents in the vicinity of the sulfur atoms should further restrict their conformational freedom. As a matter of fact, the largest deviation from perpendicularity was observed in compound **6e**, which featured a flexible *n*-butyl chain rather than a more rigid cycloalkyl or aryl group next to the attachment point of the CS_2_^−^ group. The twisted nature of the zwitterions prevented any electronic communication between their anionic and cationic parts in the solid state, in line with their cross-conjugated or pseudo-cross-conjugated nature [80–81]. Indeed, an average C1–C2 distance of 1.49 Å indicated that the adducts were essentially assembled via the formation of a single rather than a double C–C bond (1.51 vs 1.34 Å) [78]. These data are in line with the trends observed on ^13^C NMR and IR spectroscopies for δ CS_2_ and ν̃ CS_2_ and support the hypothesis that the perpendicular arrangement between the CS_2_^−^ and CCN^+^ units is retained in solution. Likewise, all the exocyclic C–C and C–N bond lengths in the crystal structures under examination were typical of single rather than double bonds (see for instance the N–Mes distances N1–C8 or N1–C4 in Table 3). This observation, combined with the orthogonal disposition of all the aryl substituents relative to the central heterocycle evidenced by C2–N1–C8–C9 or C2–N1–C4–C5 dihedral angles close to 90°, evidenced the lack of conjugation between the heterocyclic core of the molecules and their peripherical decorations.

**Table 3:** Selected bond lengths (Å) and angles (°) derived from the molecular structures of various CAAC·CS_2_, MIC·CS_2_, and NHC·CS_2_ zwitterions.^a^

compound	C1–S1	C1–S2	C1–C2	C2–C3 (or C2–C5)	C2–N1	N1–C8 (or N1–C4)

**4a**	1.664(2)	1.671(2)	1.483(3)	1.520(2)	1.302(3)	1.456(2)
**4c**	1.675(1)	1.661(2)	1.487(2)	1.529(2)	1.302(2)	1.465(2)
**6b**	1.661(1)	1.672(1)	1.491(2)	1.384(2)	1.367(1)	1.446(1)
**6e**	1.680(2)	1.674(2)	1.486(3)	1.383(2)	1.365(2)	1.449(2)
**IMes·CS** **_2_** ^b,c^	1.667(3)	1.667(3)	1.489(7)	/	1.336(5)	1.461(6)
**SIMes·CS** **_2_** ^b,d^	1.662(2)	1.662(2)	1.502(6)	/	1.315(4)	1.446(4)

compound	S1–C1–S2	N1–C2–C5 (or N1–C2–C3)	S1–C1–C2–N1	N1–C3–C4–C5 (or N1–N2–N3–C3)	C2–N1–C8–C9 (or C2–N1–C4–C5)	

**4a**	131.4(2)	112.2(1)	86.9(2)	−23.0(2)	85.6(2)	
**4c**	131.17(9)	112.4(1)	91.6(2)	12.2(2)	90.1(2)	
**6b**	129.00(7)	104.57(9)	87.7(1)	0.3(1)	94.2(1)	
**6e**	128.5(1)	104.9(1)	126.3(2)	0.8(2)	100.1(2)	
**IMes·CS** **_2_** ^b,c^	129.1(4)	107.2(4)	114.7(2)	0.5(5)	104.9(6)	
**SIMes·CS** **_2_** ^b,d^	130.3(3)	112.0(4)	92.4(2)	9.5(5)	94.4(5)	

^a^See Figure 3 and Figure 4 for atom labeling. ^b^Data from ref. [40]. ^c^IMes·CS_2_ crystallized with two molecules in the asymmetric unit. ^d^Only half of the molecule of SIMes·CS_2_ formed the asymmetric unit.

## Conclusion

The synthesis of three CAAC·CS_2_ and six MIC·CS_2_ zwitterions derived from aldiminium or 1,2,3-triazolium salts was achieved via a two-step procedure involving the in situ generation of free carbenes with a strong base, followed by their nucleophilic addition onto carbon disulfide. The nine products obtained were characterized by ^1^H and ^13^C NMR spectroscopy, FTIR spectroscopy, HR–ESI mass spectrometry, and elemental analysis. Moreover, the molecular structures of two CAAC·CS_2_ and two MIC·CS_2_ betaines were determined by X-ray diffraction analysis. The various analytical data recorded for all these compounds were compared with those reported previously for related NHC·CS_2_ zwitterions derived from imidazolinium or (benz)imidazolium salts.

Due to the absence of electronic communication between the CS_2_ unit and the orthogonal heterocycle, all the CAAC·CS_2_, MIC·CS_2_, and NHC·CS_2_ zwitterions under scrutiny displayed rather similar electronic properties and featured the same bite angle. Yet, their steric properties are liable to ample modifications by varying the nature of the cationic heterocycle and its substituents. The synthesis of 1,2,3-triazolium salts via a “click” reaction is a particularly attractive and straightforward strategy to prepare dithiocarboxylate zwitterions with two different alkyl or aryl groups flanking the carbenoid center and the adjacent CS_2_ unit. This is in sharp contrast with the most common cyclization processes leading to imidazol(in)ium derivatives, which afford symmetrical products with identical substituents on both nitrogen atoms [82]. Although cyclic aldiminium salts are less readily available, they feature a quaternary carbon atom next to the carbenoid center that may act as a source of chirality. Thus, the novel compounds reported in this study represent a valuable addition to the family of neutral dithiolate ligands derived from stable nucleophilic carbenes, and we are currently investigating their coordination chemistry toward various transition metals. Details of these studies will be disclosed in a forthcoming publication.

## Supporting Information

File 1 Experimental procedures, X-ray crystal structure determinations, copies of ^1^H NMR, ^13^C NMR, and FTIR spectra.

## Data Availability

All data that supports the findings of this study is available in the published article and/or the supporting information to this article.
