# Supplementary material for: Aldiminium and 1,2,3-triazolium dithiocarboxylate zwitterions derived from cyclic (alkyl)(amino) and mesoionic carbenes
Source: Beilstein J Org Chem. 2023 Dec 20;19:1947–56. doi: 10.3762/bjoc.19.145 (PMC10760461; doi:10.3762/bjoc.19.145)
Supplement: File 1 — Experimental procedures, X-ray crystal structure determinations, copies of 1H NMR, 13C NMR, and FTIR spectra. [file Beilstein_J_Org_Chem-19-1947-s001.pdf]

BEILSTEIN JOURNAL OF ORGANIC CHEMISTRY

Supporting Information

for

Aldiminium and 1,2,3-triazolium dithiocarboxylate zwitterions derived from cyclic (alkyl)(amino) and mesoionic carbenes

Nedra Touj, François Mazars, Guillermo Zaragoza and Lionel Delaude

Beilstein J. Org. Chem. **2023,** *19,* 1947–1956. doi:10.3762/bjoc.19.145

Experimental procedures, X-ray crystal structure determinations, copies of ^{1}H NMR, ^{13}C NMR, and FTIR spectra

License and Terms: This is a supporting information file under the terms of the Creative Commons Attribution License (https://creativecommons.org/licenses/by/4.0). Please note that the reuse, redistribution and reproduction in particular requires that the author(s) and source are credited and that individual graphics may be subject to special legal provisions.
The license is subject to the *Beilstein Journal of Organic Chemistry* terms and conditions: (https://www.beilstein-journals.org/bjoc/terms)

Table of contents

1. General information S2

2. Synthesis of triazolium iodides **5a–f** and their precursors S2
   2.1. Synthesis of 2-azido-1,3,5-trimethylbenzene S2
   2.2. Synthesis of 1-mesityl-4-phenyl-1*H*-1,2,3-triazole S3
   2.3. Synthesis of 4-butyl-1-mesityl-1*H*-1,2,3-triazole S4
   2.4. Synthesis of 1-(2,6-diisopropylphenyl)-4-phenyl-1*H*-1,2,3-triazole S4
   2.5. Synthesis of 1-mesityl-3-methyl-4-phenyl-1*H*-1,2,3-triazol-3-ium iodide (**5a**) S5
   2.6. Synthesis of 1-(2,6-diisopropylphenyl)-3-methyl-4-phenyl-1*H*-1,2,3-triazol-3-ium iodide (**5b**) S6
   2.7. Synthesis of 3-ethyl-1-mesityl-4-phenyl-1*H*-1,2,3-triazol-3-ium iodide (**5c**) S6
   2.8. Synthesis of 3-isopropyl-1-mesityl-4-phenyl-1*H*-1,2,3-triazol-3-ium iodide (**5d**) S7
   2.9. Synthesis of 4-butyl-1-mesityl-3-methyl-1*H*-1,2,3-triazol-3-ium iodide (**5e**) S7
   2.10. Synthesis of 4-butyl-3-ethyl-1-mesityl-1*H*-1,2,3-triazol-3-ium iodide (**5f**) S8

3. Synthesis of CAAC·CS_2 zwitterions with $KN(SiMe_3)_2$ S8
   3.1. Typical procedure S8
   3.2. Analytical data for CAAC-Mes-Cy·CS_2 (**4a**) S9
   3.3. Analytical data for CAAC-Dip-Cy·CS_2 (**4b**) S9
   3.4. Analytical data for CAAC-Die-MePh·CS_2 (**4c**) S10

4. Synthesis of MIC·CS_2 zwitterions with $KN(SiMe_3)_2$ S10
   4.1. Typical procedure S10
   4.2. Analytical data for MIC-Mes-Ph-Me·CS_2 (**6a**) S11
   4.3. Analytical data for MIC-Dip-Ph-Me·CS_2 (**6b**) S11

5. Synthesis of MIC·CS_2 zwitterions with NaO^tBu S11
   5.1. Typical procedure S11
   5.2. Analytical data for MIC-Mes-Ph-Et·CS_2 (**6c**) S12
   5.3. Analytical data for MIC-Mes-Ph-iPr·CS_2 (**6d**) S12
   5.4. Analytical data for MIC-Mes-Bu-Me·CS_2 (**6e**) S12
   5.5. Analytical data for MIC-Mes-Bu-Et·CS_2 (**6f**) S13

6. X-ray crystal structure determinations S13
   6.1. Experimental details S13
   6.2. Crystal data for CAAC-Mes-Cy·CS_2 (**4a**) S14
   6.3. Crystal data for CAAC-Die-MePh·CS_2 (**4c**) S14
   6.4. Crystal data for MIC-Dip-Ph-Me·CS_2 (**6b**) S14
   6.5. Crystal data for MIC-Mes-Bu-Me·CS_2 (**6e**) S14

7. References S15

8. NMR Spectra S16

9. IR Spectra S35

1. General information

The aldiminium hydrogen dichloride **3b** (CAAC-Dip-Cy·2HCl) was purchased from TCI and the tetrafluoroborate salts **3a** (CAAC-Mes-Cy·HBF_4) (Apeiron AS1056) and **3c** (CAAC-Die-MePh·HBF_4) (Apeiron AS1042) were kindly supplied by Apeiron Synthesis. All the syntheses of dithiocarboxylate zwitterions were carried out under a dry argon or nitrogen atmosphere using standard Schlenk techniques. Solvents were distilled from appropriate drying agents and deoxygenated prior to use. Sodium *tert*-butoxide and potassium bis(trimethylsilyl)amide (1 M solution in THF) were purchased from Aldrich. All the other chemicals were purchased from Aldrich or TCI and used without any further purification. Melting points were measured with an Electrothermal 9100 apparatus and are not corrected. ^{1}H and ^{13}C NMR spectra were recorded at 298 K on a Bruker DRX 400 spectrometer operating at 400.13 and 100.62 MHz, respectively. Chemical shifts are listed in parts per million downfield from TMS and are referenced from the solvent peaks or TMS. Infrared spectra were recorded in the ATR mode on a Nicolet iS5 FT-IR spectrometer. Electrospray ionization mass spectra were obtained using a Micromass LCT Premier instrument. Elemental analyses were carried out in the Laboratory of Pharmaceutical Chemistry at the University of Liège.

2. Synthesis of triazolium iodides 5a–f and their precursors

2.1. Synthesis of 2-azido-1,3,5-trimethylbenzene

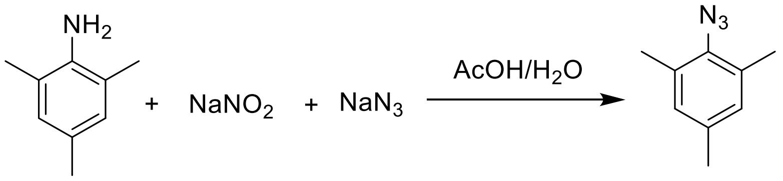

Caution: azides are heat- and shock-sensitive compounds that may decompose violently. They must be handled using appropriate safety precautions. Any attempt to scale up the procedures listed below should be carefully evaluated.

A saturated aqueous solution of $NaNO_2$ (5.17 g, 75 mmol) was added to a mixture of 2,4,6-tri-methylaniline (6.46 g, 48 mmol), water (20 mL), and AcOH (20 mL) cooled to 0 °C in an ice/water bath. After 1 h, a saturated aqueous solution of NaN_3 (5.72 g, 88 mmol) was added dropwise at 0 °C. The reaction mixture was warmed to room temperature and further stirred for 1 h. It was diluted with Et_2O (100 mL) and distilled water (100 mL). Solid Na_2CO_3 was slowly added until the pH of the aqueous phase reached 7. The two phases were separated and the aqueous phase was

extracted with additional Et_2O (2 × 60 mL). The combined organic phases were washed with distilled water (40 mL) and brine (40 mL). The resulting organic phase was dried over anhydrous $MgSO_4$ and filtered. The filtrate was concentrated on a rotary evaporator and dried under high vacuum to afford the desired mesityl azide as a yellow-orange oil kept in a refrigerator (6.96 g, 90% yield).

^{1}H NMR (400 MHz, $CDCl_3$, 298 K): δ = 6.84 (s, 2H, *m*-CH_{ar}), 2.34 (s, 6H, *o*-CH_3), 2.26 ppm (s, 3H, *p*-CH_3). ^{13}C NMR (100 MHz, $CDCl_3$, 298 K): δ = 135.4 (*p*-C_{ar}), 134.5 (CN), 131.9 (*o*-C_{ar}), 129.6 (*m*-CH_{ar}), 20.8 (*p*-CH_3), 18.1 ppm (*o*-CH_3). These data matched those reported in the literature [1].

2.2. Synthesis of 1-mesityl-4-phenyl-1*H*-1,2,3-triazole

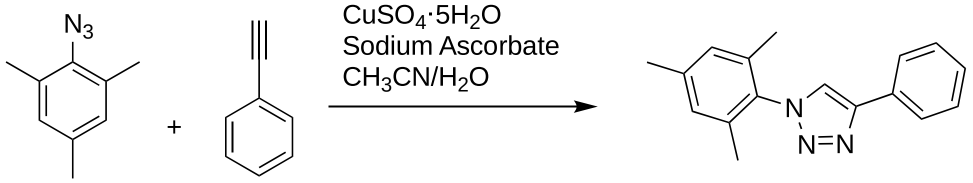

2-Azido-1,3,5-trimethylbenzene (6.7 g, 42 mmol) was dissolved in CH_3CN (27 mL) in a 100 mL round-bottomed flask and the solution was cooled to 0 °C in an ice/water bath. A solution of $CuSO_4{\cdot}5H_2O$ (1.04 g, 4.16 mmol) and sodium ascorbate (3.95 g, 20 mmol) in water (14 mL) followed by phenylacetylene (6.45 g, 63.17 mmol) were added dropwise. The reaction mixture was slowly warmed to room temperature and stirred overnight. Acetonitrile was removed on a rotary evaporator and a small amount of CH_2Cl_2 was added to dissolve the solid materials. The reaction was quenched by adding a concentrated solution of aqueous NH_4OH until the pH became basic and the resulting mixture was stirred for 3 h at room temperature. It was extracted with CH_2Cl_2 (4 × 30 mL) and the combined organic phases were dried over anhydrous $MgSO_4$. The solvent was removed on a rotary evaporator and the crude product was recrystallized from *n*-heptane to yield pure 1-mesityl-4-phenyl-1*H*-1,2,3-triazole as a white solid (7.47 g, 68% yield).

^{1}H NMR (400 MHz, $CDCl_3$, 298 K): δ = 7.93 (d, *J* = 7.2 Hz, 2H, CH_{ar} Ph), 7.83 (s, 1H, NCH), 7.47 (t, *J* = 7.6 Hz, 2H, CH_{ar} Ph), 7.37 (t, *J* = 7.4 Hz, 1H, CH_{ar} Ph), 7.01 (s, 2H, *m*-CH_{ar} Mes), 2.37 (s, 3H, *p*-CH_3 Mes), 2.02 ppm (s, 6H, *o*-CH_3 Mes). ^{13}C NMR (100 MHz, $CDCl_3$, 298 K): δ = 147.7 (C_{ar}), 140.2 (*p*-C_{ar} Mes), 135.3 (*o*-C_{ar} Mes), 133.6 (*i*-C_{ar} Mes), 130.6 (*i*-C_{ar} Ph), 129.3 (*m*-CH_{ar} Mes), 129.1 (*o*-CH_{ar} Ph), 128.4 (*p*-CH_{ar} Ph), 125.9 (*m*-CH_{ar} Ph), 121.6 (NCH), 21.3 (*p*-CH_3 Mes), 17.5 ppm (*o*-CH_3 Mes). These data matched those reported in the literature [2].

2.3. Synthesis of 4-butyl-1-mesityl-1*H*-1,2,3-triazole

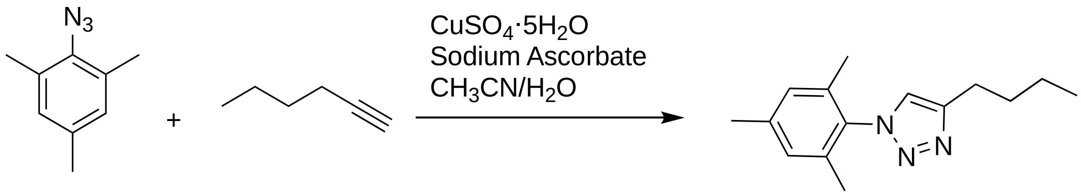

2-Azido-1,3,5-trimethylbenzene (3.44 g, 15 mmol) was dissolved in CH_3CN (10 mL) in a 50 mL round-bottomed flask and the solution was cooled to 0 °C in an ice/water bath. A solution of $CuSO_4{\cdot}5H_2O$ (0.37 g, 1.5 mmol) and sodium ascorbate (1.39 g, 7 mmol) in water (5 mL) followed by 1-hexyne (1.83 g, 22.3 mmol), were added dropwise. The reaction mixture was slowly warmed to room temperature and stirred overnight. The reaction was quenched by adding a concentrated solution of aqueous NH_4OH until the pH became basic and the resulting mixture was stirred overnight at room temperature. It was extracted with CH_2Cl_2 (4 × 20 mL) and the combined organic phases were dried over anhydrous $MgSO_4$. The solvent was removed on a rotary evaporator and the residue was dried under high vacuum to yield 4-butyl-1-mesityl-1*H*-1,2,3-triazole as a dark yellow oil (3.14 g, 88% yield).

^1H NMR (400 MHz, $CDCl_3$, 298 K): δ = 7.29 (s, 1H, NCH), 6.92 (s, 2H, *m*-CH_{ar} Mes), 2.78 (t, *J* = 7.7 Hz, 2H, CH_2 Bu), 2.29 (s, 3H, *p*-CH_3 Mes), 1.90 (s, 6H, *o*-CH_3 Mes), 1.69 (quint, *J* = 7.6 Hz, 2H, CH_2 Bu), 1.38 (sext, *J* = 7.4 Hz, 2H, CH_2 Bu), 0.91 ppm (t, *J* = 7.4 Hz, 3H, CH_3 Bu). ^{13}C NMR (100 MHz, $CDCl_3$, 298 K): δ = 148.1 (C_{ar}), 139.7 (*p*-C_{ar} Mes), 135.1 (*o*-C_{ar} Mes), 133.7 (*i*-C_{ar} Mes), 128.9 (*m*-CH_{ar} Mes), 122.5 (NCH), 31.5 (*C*$H_2CH_2CH_2$ Bu), 25.3 (CH_2*C*H_2CH_2 Bu), 22.3 (*C*H_2CH_3 Bu), 21.1 (*p*-CH_3 Mes), 17.2 (*o*-CH_3 Mes), 13.8 ppm (CH_3 Bu). These data matched those reported in the literature [3].

2.4. Synthesis of 1-(2,6-diisopropylphenyl)-4-phenyl-1*H*-1,2,3-triazole

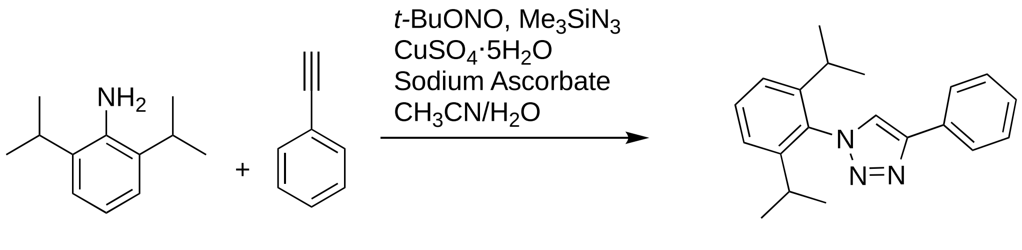

Freshly distilled 2,6-diisopropylaniline (5.56 g, 31 mmol) was dissolved in CH_3CN (20 mL) in a 50 mL round-bottomed flask and the solution was cooled to 0 °C in an ice/water bath. 90% *t*-BuONO (6.21 mL, 4.85 g, 47 mmol) and 95% Me_3SiN_3 (5.12 mL, 4.34 g, 37 mmol) were sequentially added dropwise at 0 °C, and stirring was maintained for 2 h at 0 °C. A solution of $CuSO_4{\cdot}5H_2O$ (0.79 g, 3.1 mmol) and sodium ascorbate (3.09 g, 15 mmol) in water (10 mL) followed by phenylacetylene

(4.74 g, 47 mmol) were added dropwise. The reaction mixture was slowly warmed to room temperature and stirred overnight. The reaction was quenched by adding a concentrated solution of aqueous NH_4OH until the pH became basic and the resulting mixture was stirred overnight at room temperature. It was extracted with CH_2Cl_2 (4 × 25 mL) and the combined organic phases were dried over anhydrous $MgSO_4$. The solvent was removed on a rotary evaporator and the crude product was washed with *n*-pentane to yield 1-(2,6-diisopropylphenyl)-4-phenyl-1*H*-1,2,3-triazole as a white solid (2.86 g, 30% yield).

^1H NMR (400 MHz, $CDCl_3$, 298 K): δ = 8.01 – 7.94 (m, 2H, CH_{ar} Ph), 7.9 (s, 1H, NCH), 7.52 (dt, J = 13.3, 7.8 Hz, 3H, CH_{ar} Ph), 7.43 – 7.37 (m, 1H, CH_{ar} Dip), 7.34 (d, J = 7.8 Hz, 2H, CH_{ar} Dip), 2.37 (sept, J = 6.8 Hz, 2H, CH iPr), 1.20 (d, J = 8.0, Hz, 6H, CH_3 iPr), 1.18 ppm (d, J = 4.0 Hz, 6H, CH_3 iPr). ^{13}C NMR (100 MHz, $CDCl_3$, 298 K): δ = 147.4 (C_{ar}), 146.1 (*o*-C_{ar} Dip), 133.2 (*i*-C_{ar} Dip), 130.9 (*i*-C_{ar} Ph), 130.4 (*p*-CH_{ar} Ph), 129.0 (*m*-CH_{ar} Ph), 128.4 (*p*-CH_{ar} Dip), 125.8 (*o*-CH_{ar} Ph), 123.9 (*m*-CH_{ar} Dip), 122.4 (NCH), 28.4 (CH iPr), 24.4 (CH_3 iPr), 24.1 ppm (CH_3 iPr). These data matched those reported in the literature [4].

2.5. Synthesis of 1-mesityl-3-methyl-4-phenyl-1*H*-1,2,3-triazol-3-ium iodide (**5a**)

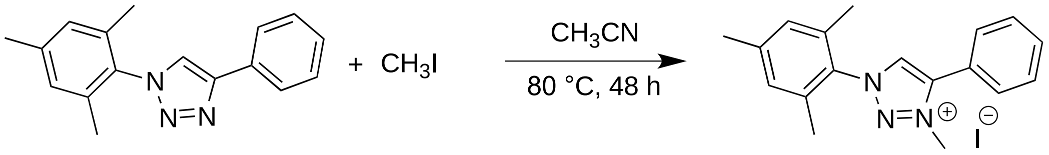

1-Mesityl-4-phenyl-1*H*-1,2,3-triazole (2.5 g, 9.5 mmol), acetonitrile (16 mL), and methyl iodide (8.1 g, 57 mmol, 6 equiv) were stirred and heated in a closed Schlenk tube placed in an oil bath at 80 °C for 48 h. After cooling to room temperature, the solvent was removed on a rotary evaporator. The orange sticky residue was washed with hot ethyl acetate (6 × 10 mL) and dried under high vacuum to afford 1-mesityl-3-methyl-4-phenyl-1*H*-1,2,3-triazol-3-ium iodide as an off-white solid (3.6 g, 94% yield).

^1H NMR (400 MHz, $CDCl_3$, 298 K): δ = 9.03 (s, 1H, NCH), 8.06 – 7.95 (m, 2H, *m*-CH_{ar} Mes), 7.60 (dd, J = 5.3, 1.9 Hz, 3H, CH_{ar} Ph), 7.05 (s, 2H, CH_{ar} Ph), 4.57 (s, 3H, CH_3 Me), 2.37 (s, 3H, *p*-CH_3 Mes), 2.21 ppm (s, 6H, *o*-CH_3 Mes). ^{13}C NMR (100 MHz, $CDCl_3$, 298 K): δ = 144.4 (C_{ar}), 142.8 (*p*-C_{ar} Mes), 134.6 (*o*-C_{ar} Mes), 132.3 (*p*-CH_{ar} Ph), 131.3 (*i*-C_{ar} Mes), 130.6 (*m*-CH_{ar} Mes), 130.3 (NCH), 130.1 (*o*-CH_{ar} Ph), 129.9 (*m*-CH_{ar} Ph), 121.4 (*i*-C_{ar} Ph), 40.8 (CH_3 Me), 21.4 (*p*-CH_3 Mes), 18.7 ppm (*o*-CH_3 Mes). These data matched those reported in the literature [5].

2.6. Synthesis of 1-(2,6-diisopropylphenyl)-3-methyl-4-phenyl-1*H*-1,2,3-triazol-3-ium iodide (**5b**)

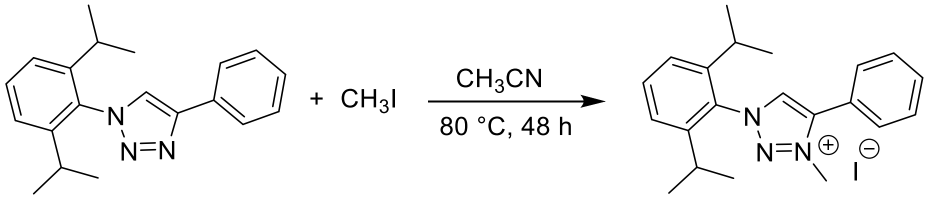

1-(2,6-Diisopropylphenyl)-4-phenyl-1*H*-1,2,3-triazole (2.86 g, 9.4 mmol), acetonitrile (17 mL), and methyl iodide (8.04 g, 56.4 mmol, 6 equiv) were stirred and heated in a closed Schlenk tube placed in an oil bath at 80 °C for 48 h. After cooling to room temperature, the solvent was removed on a rotary evaporator. The solid residue was washed with ethyl acetate (5 × 10 mL) and dried under high vacuum to afford 1-(2,6-diisopropylphenyl)-3-methyl-4-phenyl-1*H*-1,2,3-triazol-3-ium iodide as an off-white solid (3.23 g, 76% yield).

^{1}H NMR (400 MHz, $CDCl_3$, 298 K): δ = 8.77 (s, 1H, NCH), 8.14 – 8.07 (m, 2H, CH_{ar} Ph) 7.68 – 7.58 (m, 4H, 3H CH_{ar} Ph + 1H *p*-CH_{ar} Dip), 7.39 (d, *J* = 7.9 Hz, 2H, *m*-CH_{ar} Dip), 4.66 (s, 3H, CH_3 Me), 2.53 (hept, *J* = 6.7 Hz, 2H, CH iPr), 1.26 (d, *J* = 8.0 Hz, 6H, CH_3 iPr), 1.23 ppm (d, *J* = 8.0 Hz, 6H, CH_3 iPr). ^{13}C NMR (100 MHz, $CDCl_3$, 298 K): δ = 145.8 (C_{ar}), 144.5 (*o*-C_{ar} Dip), 133.1 (*p*-CH_{ar} Ph), 132.4 (*p*-CH_{ar} Dip), 130.7 (*m*-CH_{ar} Ph), 130.6 (NCH), 129.9 (*o*-CH_{ar} Ph), 125.0 (CH_{ar} Dip), 121.2 (*i*-C_{ar} Ph), 41.2 (CH_3 Me), 29.0 (CH iPr), 24.8 (CH_3 iPr), 24.2 (CH_3 iPr). These data matched those reported in the literature [6].

2.7. Synthesis of 3-ethyl-1-mesityl-4-phenyl-1*H*-1,2,3-triazol-3-ium iodide (**5c**)

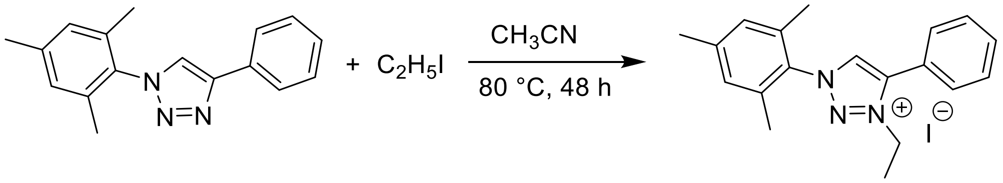

1-Mesityl-4-phenyl-1*H*-1,2,3-triazole (1.5 g, 5.7 mmol), acetonitrile (16 mL), and ethyl iodide (5.33 g, 34 mmol, 6 equiv) were stirred and heated in a closed Schlenk tube placed in an oil bath at 80 °C for 48 h. After cooling to room temperature, the solvent was removed on a rotary evaporator. The dark red sticky residue was washed with hot ethyl acetate (6 × 10 mL) and dried under high vacuum to afford 3-ethyl-1-mesityl-4-phenyl-1*H*-1,2,3-triazol-3-ium iodide as a pale orange powder (1.28 g, 54% yield).

^{1}H NMR (400 MHz, $CDCl_3$, 298 K): δ = 8.90 (s, 1H, NCH), 8.05 – 7.96 (m, 2H, *m*-CH_{ar} Mes), 7.62 (dd, *J* = 5.2, 1.9 Hz, 3H, CH_{ar} Ph), 7.07 (s, 2H, CH_{ar} Ph), 4.92 (q, *J* = 7.3, 2H, CH_2 Et), 2.38 (s, 3H, *p*-CH_3

Mes), 2.23 (s, 6H, *o*-CH_3 Mes), 1.68 ppm (t, *J* = 7.3, 3H, CH_3 Et). ^{13}C NMR (100 MHz, $CDCl_3$, 298 K): δ = 143.8 (C_{ar}), 142.8 (*p*-C_{ar} Mes), 134.6 (*o*-C_{ar} Mes), 132.3 (*p*-CH_{ar} Ph), 131.4 (*i*-C_{ar} Mes), 130.7 (NCH), 130.5 (*m*-CH_{ar} Mes), 130.2 (*o*-CH_{ar} Ph), 130.0 (*m*-CH_{ar} Ph), 121.4 (*i*-C_{ar} Ph), 49.3 (CH_2 Et), 21.4 (*p*-CH_3 Mes), 18.6 (*o*-CH_3 Mes), 14.3 ppm (CH_3 Et).

2.8. Synthesis of 3-isopropyl-1-mesityl-4-phenyl-1*H*-1,2,3-triazol-3-ium iodide (**5d**)

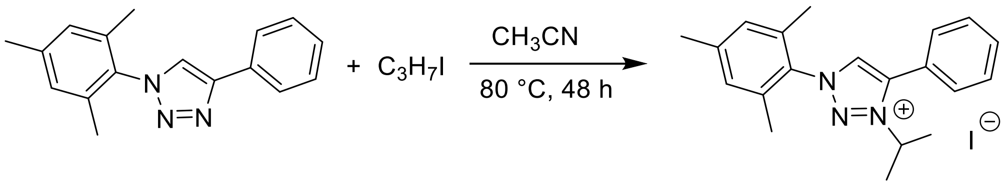

1-Mesityl-4-phenyl-1*H*-1,2,3-triazole (3.16 g, 12 mmol), acetonitrile (20 mL), and 2-iodopropane (12.24 g, 72 mmol, 6 equiv) were stirred and heated in a closed Schlenk tube placed in an oil bath at 80 °C for 48 h. After cooling to room temperature, the solvent was removed on a rotary evaporator. The sticky residue was washed with ethyl acetate (6 × 10 mL) and dried under high vacuum to afford 3-isopropyl-1-mesityl-4-phenyl-1*H*-1,2,3-triazol-3-ium iodide as a pale yellow solid (2.3 g, 44% yield).

^1H NMR (400 MHz, $CDCl_3$, 298 K): δ = 9.43 (s, 1H, NCH), 7.98 – 7.90 (m, 2H, CH_{ar} Ph), 7.62 (m, *J* = 4.9, 1.9 Hz, 3H, CH_{ar} Ph), 7.05 (s, 2H, *m*-CH_{ar} Mes), 5.24 (hept, *J* = 6.7, 2H, CH iPr), 2.36 (s, 3H, *p*-CH_3 Mes), 2.18 (s, 6H, *o*-CH_3 Mes), 1.73 ppm (d, *J* = 7.3, 3H, CH_3 iPr). ^{13}C NMR (100 MHz, $CDCl_3$, 298 K): δ = 143.1 (*p*-C_{ar} Mes), 142.7 (C_{ar}), 134.3(*o*-C_{ar} Mes), 132.3 (*m*-CH_{ar} Mes), 131.4 (*i*-C_{ar} Mes), 130.7 (*p*-CH_{ar} Ph), 130.5 (*o*-CH_{ar} Mes), 130.2 (*m*-CH_{ar} Ph), 130.0 (NCH), 121.4 (*i*-C_{ar} Ph), 49.3 (CH_3), 21.4 (*p*-CH_3 Mes), 18.6 (*o*-CH_3 Mes), 14.3 ppm (CH_3 Et).

2.9. Synthesis of 4-butyl-1-mesityl-3-methyl-1*H*-1,2,3-triazol-3-ium iodide (**5e**)

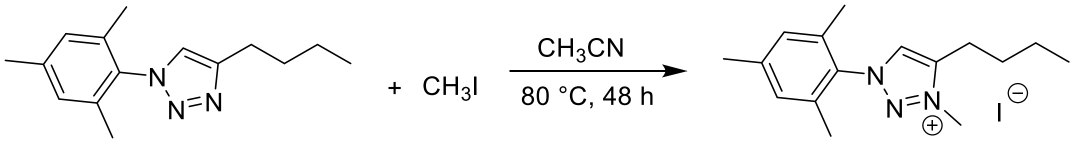

4-Butyl-1-mesityl-1*H*-1,2,3-triazole (1.5 g, 6.2 mmol), acetonitrile (11 mL), and methyl iodide (5.25 g, 37 mmol, 6 equiv) were stirred and heated in a closed Schlenk tube placed in an oil bath at 80 °C for 48 h. After cooling to room temperature, the solvent was removed on a rotary evaporator. The brown solid residue was washed with hot ethyl acetate (1 × 60 mL) and dried under high vacuum to afford 4-butyl-1-mesityl-3-methyl-1*H*-1,2,3-triazol-3-ium iodide as a white solid (1.93 g, 81% yield).

^{1}H NMR (400 MHz, $CDCl_3$, 298 K): δ = 8.46 (s, 1H, NCH), 7.03 (s, 2H, *m*-CH_{ar} Mes), 4.51 (s, 3H, CH_3 Me), 3.24 (t, *J* = 7.9 Hz, 2H, CH_2 Bu), 2.36 (s, 3H, *p*-CH_3 Mes), 2.11 (s, 6H, *o*-CH_3 Mes), 1.84 (quint, *J* = 7.6 Hz, 2H, CH_2 Bu), 1.51 (sext, *J* = 7.4 Hz, 2H, CH_2 Bu), 0.98 ppm (t, *J* = 7.3 Hz, 3H, CH_3 Bu). ^{13}C NMR (100 MHz, $CDCl_3$, 298 K): δ = 146.5 (C_{ar}), 142.6 (*p*-C_{ar} Mes), 134.5 (*o*-C_{ar} Mes), 131.3 (*i*-C_{ar} Mes), 130.3 (NCH), 130.0 (*m*-CH_{ar} Mes), 40.0 (CH_3 Me), 29.7 ($\mathit{C}H_2CH_2CH_2$ Bu), 24.6 ($CH_2\mathit{C}H_2CH_2$ Bu), 22.3 ($\mathit{C}H_2CH_3$ Bu), 21.3 (*p*-CH_3 Mes), 18.2 (*o*-CH_3 Mes), 13.9 ppm (CH_3 Bu).

2.10. Synthesis of 4-butyl-3-ethyl-1-mesityl-1*H*-1,2,3-triazol-3-ium iodide (**5f**)

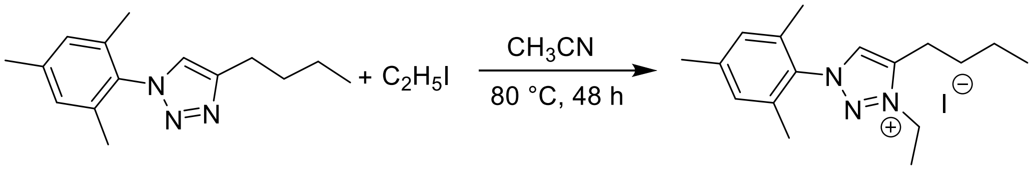

4-Butyl-1-mesityl-1*H*-1,2,3-triazole (1.5 g, 6.2 mmol), acetonitrile (11 mL), and ethyl iodide (5.77 g, 37 mmol, 6 equiv) were stirred and heated in a closed Schlenk tube placed in an oil bath at 80 °C for 48 h. After cooling to room temperature, the solvent was removed on a rotary evaporator. The solid residue was washed with hot ethyl acetate (1 × 30 mL) and dried under high vacuum to afford 4-butyl-3-ethyl-1-mesityl-1*H*-1,2,3-triazol-3-ium iodide as a pale yellow solid (2.94 g, 84% yield).

^{1}H NMR (400 MHz, $CDCl_3$, 298 K): δ = 8.55 (s, 1H, NCH), 7.05 (s, 2H, *m*-CH_{ar} Mes), 4.81 (q, *J* = 7.3 Hz, 2H, CH_2 Et), 3.26 (t, *J* = 7.9 Hz, 2H, CH_2 Bu), 2.37 (s, 3H, *p*-CH_3 Mes), 2.12 (s, 6H, *o*-CH_3 Mes), 1.86 (sext, *J* = 7.8 Hz, 2H, CH_2 Bu), 1.74 (t, *J* = 7.3 Hz, 3H, CH_3 Et), 1.52 (quint, *J* = 7.4 Hz, 2H, CH_2 Bu), 1.00 ppm (t, *J* = 7.3 Hz, 3H, CH_3 Bu). ^{13}C NMR (100 MHz, $CDCl_3$, 298 K): δ = 145.9 (C_{ar}), 142.7 (*p*-C_{ar} Mes), 134.5 (*o*-C_{ar} Mes), 131.5 (*i*-C_{ar} Mes), 130.5 (NCH), 130.0 (*m*-CH_{ar} Mes), 48.4 (CH_2 Et), 29.6 ($\mathit{C}H_2CH_2CH_2$ Bu), 24.5 ($CH_2\mathit{C}H_2CH_2$ Bu), 22.4 ($\mathit{C}H_2CH_3$ Bu), 21.4 (*p*-CH_3 Mes), 18.1 (*o*-CH_3 Mes), 14,0 (CH_3 Et), 13.9 ppm (CH_3 Bu).

3. Synthesis of CAAC·CS_2 zwitterions with $KN(SiMe_3)_2$

3.1. Typical procedure

An oven-dried 50 mL round-bottomed flask equipped with a magnetic stirring bar and capped with a three-way stopcock was charged with an aldiminium salt (2.5 mmol). The reactor was purged of air by applying three vacuum/nitrogen cycles before dry THF (25 mL) was added. The suspension was cooled to 0 °C in an ice/water bath before a 1 M solution of $KN(SiMe_3)_2$ in THF (3 mL, 3 mmol) was slowly added with a syringe. The cooling bath was removed and the reaction mixture was

stirred for 30 min at room temperature. After the solid had settled down, the supernatant solution was transferred with a cannula and filtered through Celite under an inert atmosphere into a two-neck 100 mL round-bottomed flask equipped with a magnetic stirring bar and capped with a three-way stopcock. The filtering device was rinsed twice with dry THF (2 × 5 mL). Carbon disulfide (0.5 mL, 8.3 mmol) was added with a syringe to the carbene solution. A color change occurred instantaneously. After 30 min of stirring at room temperature, the solvent was evaporated under vacuum. The residue was brought back to air, washed twice with *n*-pentane (2 × 5 mL), and dried under high vacuum.

3.2. Analytical data for CAAC-Mes-Cy·CS_2 (**4a**)

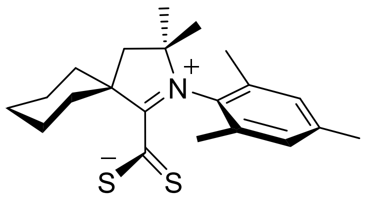

Dark red crystals obtained by recrystallization from acetonitrile (0.54 g, 60% yield); m.p. 216–218 °C (dec.). ^{1}H NMR (400 MHz, CD_2Cl_2, 298 K): δ = 6.92 (s, 2H, *m*-CH_{ar}), 2.41 (s, 2H, CH_2), 2.36 (s, 6H, *o*-CH_3), 2.27 (s, 3H, *p*-CH_3), 2.09–2.05 (m, 4H, Cy), 1.83–1.79 (m, 2H, Cy), 1.71–1.68 (m, 1H, Cy), 1.50 (s, 6H, $C(CH_3)_2$), 1.44–1.37 (m, 2H, Cy), 1.29–1.20 ppm (m, 1H, Cy). ^{13}C NMR (100 MHz, CD_2Cl_2, 298 K): δ = 230.5 (CS_2), 188.7 (*C*CS_2), 140.0 (*p*-C_{ar} Mes), 135.7 (*o*-C_{ar} Mes), 130.8 (*m*-CH_{ar} Mes), 129.8 (*i*-C_{ar} Mes), 77.8 (*C*$(CH_3)_2$), 52.8 (*spiro*-C) 46.1 (CH_2), 39.3 (CH_2 Cy), 29.1 (C(*C*$H_3)_2$), 25.0 (CH_2 Cy), 22.8 (CH_2 Cy) 22.2 (*o*-CH_3 Mes). 20.9 ppm (*p*-CH_3 Mes); IR (ATR): ν = 2922 (m), 2851 (w), 1552 (s), 1442 (m), 1377 (m), 1157 (m), 1037 (s), 983 (m), 852 (m), 702 (m), 566 (m) cm^{-1}; HRMS (ESI): *m/z* calcd for $C_{42}H_{58}N_2S_4$+K^+: 757.31144 [2*M*+K]$^+$; found: 757.31945; elemental analysis calcd for $C_{21}H_{29}NS_2$: C 70.14, H 8.13, N 3.90, S 17.83; found: C 69.82, H 8.11, N 4.02, S 16.64.

3.3. Analytical data for CAAC-Dip-Cy·CS_2 (**4b**)

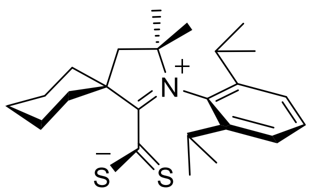

Orange-brown microcrystalline powder (0.79 g, 78% yield); m.p. 225 °C (dec.). ^{1}H NMR (400 MHz, CD_2Cl_2, 298 K): δ = 7.39 (t, *J* = 8.0 Hz, 1H, *p*-CH_{ar}), 7.24 (d, *J* = 8.0, 2H, *m*-CH_{ar}), 2.93 (sept, *J* = 8.0 Hz, 2H, CH iPr), 2.39 (s, 2H, CH_2), 2.10–2.06 (m, 4H, Cy), 1.83–1.79 (m, 2H, Cy), 1.70–1.67 (m, 1H, Cy), 1.50 (s, 6H, $C(CH_3)_2$), 1.44–1.41 (m, 2H, Cy), 1.31 (d, *J* = 8.0 Hz, 6H, CH_3 iPr), 1.28 (d, *J* = 8.0 Hz, 6H, CH_3 iPr), 1.24–1.21 ppm (m, 1H, Cy); ^{13}C NMR (100 MHz, CD_2Cl_2, 298 K): δ = 227.7 (CS_2), 189.0 (*C*CS_2), 147.1 (*o*-C Dip), 130.7 (*p*-CH Dip), 130.0 (*i*-C Dip), 126.5 (*m*-CH Dip), 77.5 (*C*$(CH_3)_2$), 53.3 (*spiro*-C) 45.9 (CH_2), 39.9 (CH_2 Cy), 29.9 (CH iPr), 29.6 (C(*C*$H_3)_2$), 28.0 (CH_3 iPr), 26.0 (CH_3 iPr), 25.0 (CH_2 Cy), 23.1 ppm (CH_2 Cy); IR (ATR): ν = 2927 (m), 2857 (m), 1536 (s), 1446 (m), 1143 (m),

1050 (s), 932 (m), 808 (m), 694 (m) cm^{-1}; HRMS (ESI): *m/z* calcd for $C_{48}H_{70}N_2S_4$+K$^+$: 841.40534 [2*M*+K]$^+$; found: 841.41947; elemental analysis calcd for $C_{24}H_{35}NS_2$: C 71.77, H 8.78, N 3.49, S 15.96; found: C 71.33, H 8.47, N 3.77, S 16.04.

3.4. Analytical data for CAAC-Die-MePh·CS_2 (**4c**)

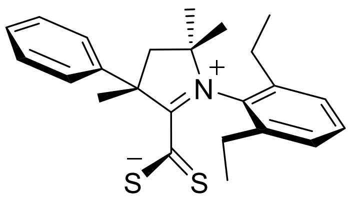

Dark red crystals obtained by recrystallization from acetonitrile (0.52 g, 52% yield); m.p. 230 °C (dec.). ^{1}H NMR (400 MHz, CD_2Cl_2, 298 K): δ = 6.69–7.67 (m, 2H, CH_{ar}), 7.41–7.25 (m, 6H, CH_{ar}), 3.17 (d, *J* = 12.0 Hz, 1H, CH_2), 3.00–2.86 (m, 2H, CH_2 Et), 3.67–2.57 (m, 2H, CH_2 Et), 2.63 (d, *J* = 12.0 Hz, 1H, CH_2), 2.37 (s, 3H, CH_3), 1.66 (s, 3H, $C(CH_3)_2$), 1.55 (s, 3H, $C(CH_3)_2$), 1.27 ppm (q, *J* = 8.0 Hz, 6H, CH_3 Et); ^{13}C NMR (100 MHz, CD_2Cl_2, 298 K): δ = 228.8 (CS_2), 188.2 (*C*CS_2), 142.5 (C_{ar}), 141.7 (C_{ar}), 141.2 (C_{ar}), 131.4 (C_{ar}), 130.4 (CH_{ar}), 128.4 (CH_{ar}), 128.2 (CH_{ar}), 128.1 (CH_{ar}), 127.6 (CH_{ar}), 127.3 (CH_{ar}), 77.3 (*C*$(CH_3)_2$), 56.2 (CMePh), 51.8 (CH_2), 33.7 (CH_3), 29.3 (C(*C*$H_3)_2$), 29.0 (C(*C*$H_3)_2$), 27.0 (CH_2 Et), 26.5 (CH_2 Et), 14.9 (CH_3 Et), 14.6 ppm (CH_3 Et); IR (ATR): ν = 2961 (w), 2862 (w), 1554 (s), 1456 (m), 1373 (m), 1148 (m), 1040 (s), 692 (s) cm^{-1}; HRMS (ESI): *m/z* calcd for $C_{48}H_{58}N_2S_4$+K$^+$: 829.31144 [2*M*+K]$^+$; found: 829.32580; elemental analysis calcd for $C_{24}H_{29}NS_2$: C 72.86, H 7.39, N 3.54, S 16.21; found: C 72.98, H 7.57, N 3.80, S 15.54.

4. Synthesis of MIC·CS_2 zwitterions with $KN(SiMe_3)_2$

4.1. Typical procedure

An oven-dried 25 mL round-bottomed flask equipped with a magnetic stirring bar and capped with a three-way stopcock was charged with a triazolium salt (1 mmol). The reactor was purged of air by applying three vacuum/nitrogen cycles before dry THF (10 mL) was added. The suspension was cooled to 0 °C in an ice/water bath before a 1 M solution of $KN(SiMe_3)_2$ in THF (1.2 mL, 1.2 mmol) was slowly added with a syringe. The cooling bath was removed and the reaction mixture was stirred for 30 min at room temperature. After the solid had settled down, the supernatant solution was transferred with a cannula and filtered through Celite under an inert atmosphere into a two-necked 100 mL round-bottomed flask equipped with a magnetic stirring bar and capped with a three-way stopcock. The filtering device was rinsed twice with dry THF (2 × 5 mL). Carbon disulfide (0.2 mL, 3.3 mmol) was added with a syringe to the carbene solution. A color change occurred instantaneously. After 30 min of stirring at room temperature, the solvent was evaporated under vacuum. The residue was brought back to air and washed twice with petroleum ether (2 × 10 mL). It was recrystallized from acetonitrile.

4.2. Analytical data for MIC-Mes-Ph-Me·CS_2 (**6a**)

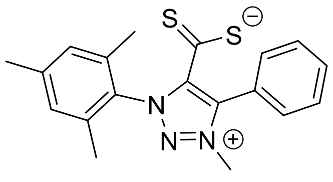

Pink solid (0.13 g, 67% yield); m.p. 192 °C (dec.). ^{1}H NMR (400 MHz, CD_2Cl_2, 298 K): δ = 7.79 (dd, *J* = 7.7, 2.0 Hz, 2H, CH_{ar} Ph), 7.66–7.53 (m, 3H, CH_{ar} Ph), 7.03 (s, 2H, *m*-CH_{ar} Mes), 4.22 (s, 3H, CH_3 Me), 2.36 (s, 3H, *p*-CH_3 Mes), 2.28 ppm (s, 6H, *o*-CH_3 Mes); ^{13}C NMR (100 MHz, CD_2Cl_2, 298 K): δ = 225.6 (CS_2), 150.5 (CCS_2), 142.5 (*p*-C_{ar} Mes), 136.3 (*o*-C_{ar} Mes), 134.9 (C_{ar}), 131.8 (*p*-CH_{ar} Ph), 130.9 (*i*-Car Mes), 129.9 (*m*-CH_{ar} Mes), 129.8 (CH_{ar} Ph), 129.7 (CH_{ar} Ph), 123.4 (*i*-C_{ar} Ph), 39.2 (CH_3 Me), 21.4 (*p*-CH_3 Mes), 18.9 ppm (*o*-CH_3 Mes); IR (ATR): ν = 2921 (w), 2852 (w), 1482 (w), 1296 (w), 1043 (s), 890 (m), 690 (m) cm^{-1}; HRMS (ESI): *m/z* calcd for $C_{38}H_{38}N_6S_4$+K^+: 745.16724 $[2M+K]^+$; found: 745.17064; elemental analysis calcd for $C_{19}H_{19}N_3S_2$: C 64.56, H 5.42, N 11.89, S 18.14; found: C 64.36, H 5.67, N 11.48, S 17.36.

4.3. Analytical data for MIC-Dip-Ph-Me·CS_2 (**6b**)

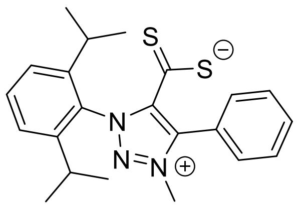

Brown solid (0.29 g, 73% yield); m.p. 189 °C (dec.). ^{1}H NMR (400 MHz, CD_2Cl_2, 298 K): δ = 7.84 (dd, *J* = 7.4, 2.2 Hz, 2H, CH_{ar} Ph), 7.65–7.53 (m, 4H, 3H CH_{ar} Ph + 1H *p*-CH_{ar} Dip), 7.34 (d, *J* = 7.8, 2H, *m*-CH_{ar} Dip), 4.23 (s, 3H, CH_3 Me), 2.76 (sept, *J* = 8.0 Hz, 2H, CH iPr), 1.31 (d, *J* = 6.7 Hz, 6H, CH_3 iPr), 1.11 ppm (d, *J* = 6.9 Hz, 6H, CH_3 iPr); ^{13}C NMR (100 MHz, CD_2Cl_2, 298 K): δ = 225.0 (CS_2), 150.5 (CCS_2), 147.0 (*o*-C_{ar} Dip), 134.1 (C_{ar}), 132.6 (*p*-CH_{ar}), 131.8 (*p*-CH_{ar}), 130.4 (i-C_{ar} Dip), 129.8 (CH_{ar} Ph), 129.7 (CH_{ar} Ph), 124.7 (CH_{ar} Dip), 123.4 (*i*-C_{ar} Ph), 39.2 (CH_3 Me), 29.9 (CH iPr), 25.8 (CH_3 iPr), 22.4 ppm (CH_3 iPr); IR (ATR): ν = 2963 (m), 2858 (w), 1465 (m), 1230 (m), 1044 (s), 895 (m) 754 (m), 693 (m) cm^{-1}; HRMS (ESI): *m/z* calcd for $C_{44}H_{50}N_6S_4$+Na^+: 829.26114 $[M+Na]^+$; found: 829.26122; elemental analysis calcd for $C_{22}H_{25}N_3S_2$: C 66.80, H 6.37, N 10.62, S 16.21; found: C 66.83, H 6.55, N 10.08, S 16.08.

5. Synthesis of MIC·CS_2 zwitterions with NaO^tBu

5.1. Typical procedure

An oven-dried 25 mL round-bottomed flask equipped with a magnetic stirring bar and capped with a three-way stopcock was charged with a triazolium salt (1 mmol) and sodium *tert*-butoxide (0.48 g, 5 mmol). The reactor was purged of air by applying three vacuum/nitrogen cycles before dry THF (10 mL) was added followed by carbon disulfide (1 mL, 16.6 mmol). The reaction mixture was stirred for 1 h in an oil bath at 60 °C. After cooling down to room temperature, the solvent was evaporated under vacuum. The residue was taken up with dichloromethane (2 × 10 mL) and

filtered through a filter paper. The filtrate was concentrated under reduced pressure. The remaining solid was washed twice with *n*-pentane (2 × 5 mL) and dried under high vacuum.

5.2. Analytical data for MIC-Mes-Ph-Et·CS_2 (**6c**)

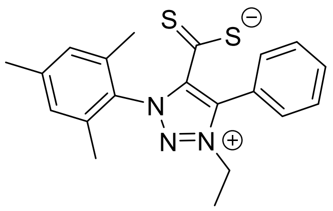

Brown solid (0.21 g, 56% yield); m.p. 180 °C (dec.). ^{1}H NMR (400 MHz, $CDCl_3$, 298 K): δ = 7.80 – 7.67 (m, 2H, CH_{ar} Ph), 7.60 – 7.43 (m, 3H, CH_{ar} Ph), 6.96 (s, 2H, *m*-CH_{ar} Mes), 4.56 (q, *J* = 7.3 Hz, 2H, CH_2 Et), 2.31 (s, 3H, *p*-CH_3 Mes), 2.27 (s, 6H, *o*-CH_3 Mes), 1.59 ppm (t, *J* = 7.3 Hz, 3H, CH_3 Et); ^{13}C NMR (100 MHz, CD_2Cl_2, 298 K): δ = 225.0 (CS_2), 150.2 (*C*CS_2), 141.8 (*p*-C_{ar} Mes), 135.8 (*o*-C_{ar} Mes), 134.3 (C_{ar}), 131.4 (*p*-CH_{ar} Ph), 130.7 (*i*-C_{ar} Mes), 129.5 (*m*-CH_{ar} Mes), 129.5 (CH_{ar} Ph), 129.4 (CH_{ar} Ph), 122.9 (*i*-C_{ar} Ph), 47.4 (CH_2 Et), 21.2 (*p*-CH_3 Mes), 18.8 (*o*-CH_3 Mes), 14.8 ppm (CH_3 Et); IR (ATR): ν = 2915 (w), 2850 (w), 1481 (m), 1221 (m), 1045 (s) 884 (m), 774 (m), 695 (m) cm^{-1}; HRMS (ESI): *m*/*z* calcd for $C_{20}H_{21}N_3S_4$+Na^+: 390.10691 [*M*+Na]$^+$; found: 390.10938; elemental analysis calcd for $C_{20}H_{21}N_3S_2$: C 65.36, H 5.76, N 11.43, S 17.45; found: C 65.83, H 5.81, N 11.12, S 16.83.

5.3. Analytical data for MIC-Mes-Ph-iPr·CS_2 (**6d**)

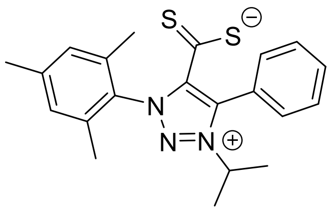

Pink solid (0.28 g, 75% yield); m.p. 220 °C (dec.). ^{1}H NMR (400 MHz, $CDCl_3$, 298 K): δ = 7.78 – 7.63 (m, 2H, CH_{ar} Ph), 7.63 – 7.42 (m, 3H, CH_{ar} Ph), 6.97 (s, 2H, *m*-CH_{ar} Mes), 4.96 (pent, *J* = 6.7 Hz, 1H, CH iPr), 1.73 – 1.61 (m, 6H, CH_3 iPr); ^{13}C NMR (100 MHz, CD_2Cl_2, 298 K): δ = 224.9 (CS_2), 150.3 (*C*CS_2), 141.9 (*p*-C_{ar} Mes), 135.9 (*o*-C_{ar} Mes), 133.9 (C_{ar}), 131.5 (*p*-CH_{ar} Ph), 131.0 (*i*-C_{ar} Mes), 129.9 (*m*-CH_{ar} Mes), 129.6 (CH_{ar} Ph), 129.5 (CH_{ar} Ph), 123.1 (*i*-C_{ar} Ph), 55.3 (CH iPr), 22.9 (CH_3 iPr), 21.3 (*p*-CH_3 Mes), 18.8 ppm (*o*-CH_3 Mes); IR (ATR): ν = 2919 (w), 2850 (w), 1480 (w), 1448 (w), 1372 (w), 1230 (m), 1047 (s), 775 (m), 698 (m) cm^{-1}; HRMS (ESI): *m*/*z* calcd for $C_{21}H_{23}N_3S_4$+Na^+: 404.12256 [*M*+Na]$^+$; found: 404.12480; elemental analysis calcd for $C_{21}H_{23}N_3S_2$: C 66.11, H 6.08, N 11.01, S 16.80; found: C 65.74, H 5.99, N 11.09, S 16.04.

5.4. Analytical data for MIC-Mes-Bu-Me·CS_2 (**6e**)

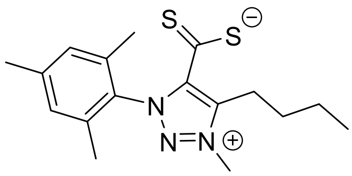

Dark brown solid (0.22 g, 61% yield); m.p. 101 °C (dec.). ^{1}H NMR (400 MHz, $CDCl_3$, 298 K): δ = 6.93 (s, 2H, *m*-CH_{ar} Mes), 4.23 (s, 3H, CH_3 Me), 3.06 (t, *J* = 8.0 Hz, 2H, CH_2 Bu), 2.30 (s, 3H, *p*-CH_3 Mes), 2.17 (s, 6H, *o*-CH_3 Mes), 1.79 (q, *J* = 7.6 Hz, 2H, CH_2 Bu), 1.46-1.41 (m, 2H, CH_2 Bu), 0.97-0.93 ppm (m, 3H, CH_3 Bu); ^{13}C NMR (100 MHz, $CDCl_3$, 298 K): δ = 225.5 (CS_2), 150.3 (*C*CS_2), 141.7 (*p*-C_{ar} Mes), 136.7 (C_{ar}), 135.8 (*o*-C_{ar} Mes), 130.8 (*i*-C_{ar} Mes), 129.4 (*m*-CH_{ar} Mes), 37.7

(CH_3 Me), 29.6 (*C*$H_2CH_2CH_2$ Bu), 23.6 (CH_2*C*H_2CH_2 Bu), 22.7 (*C*H_2CH_3 Bu), 21.3 (*p*-CH_3 Mes), 18.7 (*o*-CH_3 Mes), 13.8 ppm (CH_3 Bu); IR (ATR): ν = 2923 (m), 2857 (m), 1454 (m), 1359 (m), 1228 (s), 1044 (s), 852 (m), 606 (s) cm^{-1}; HRMS (ESI): *m*/z calcd for $C_{17}H_{23}N_3S_4$+Na^+: 356.12256 [*M*+Na]$^+$; found: 356.12422; elemental analysis calcd for $C_{17}H_{23}N_3S_2$: C 61.22, H 6.95, N 12.60, S 19.23; found: C 61.40, H 6.94, N 12.61, S 19.04.

5.5. Analytical data for MIC-Mes-Bu-Et·CS_2 (**6f**)

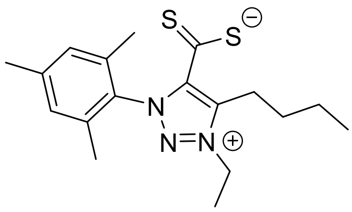

Dark brown solid (0.20 g, 59% yield), m.p. 105 °C (dec.). ^1H NMR (400 MHz, CD_2Cl_2, 298 K): δ = 6.99 (s, 2H, *m*-CH_{ar}), 4.51 (q, *J* = 7.3, 2H, CH_2 Et), 3.03 (t, *J* = 8.0 Hz, 2H, CH_2 Bu), 2.34 (s, 3H, *p*-CH_3), 2.16 (s, 6H, *o*-CH_3), 1.78 (quint, *J* = 8.0 Hz, 2H, CH_2 Bu), 1.68 (t, *J* = 8.0 Hz, 3H, CH_3 Et), 1.46 (sext, *J* = 8.0 Hz, 2H, Bu), 0.96 ppm (t, *J* = 8.0 Hz, 3H, CH_3 Bu) ; ^{13}C NMR (100 MHz, CD_2Cl_2, 298 K): δ = 226.3 (CS_2), 150.4 (*C*CS_2), 142.1 (*p*-C_{ar} Mes), 136.3 (C_{ar}), 136.2 (*o*-C_{ar} Mes), 131.3 (*i*-C_{ar} Mes), 129.5 (*m*-CH_{ar} Mes), 47.0 (CH_2 Et), 30.2 (*C*$H_2CH_2CH_2$ Bu), 23.6 (CH_2*C*H_2CH_2 Bu), 23.0 (*C*H_2CH_3 Bu), 21.3 (*p*-CH_3 Mes), 18.6 (*o*-CH_3 Mes), 14.8 (CH_3 Et), 13.8 ppm (CH_3 Bu); IR (ATR): ν = 2957 (m, 2864 (w), 1455 (m), 1225 (m), 1042 (s), 887 (m), 854 (m), 609 (m) cm^{-1}; HRMS (ESI): *m/z* calcd for $C_{36}H_{50}N_6S_4$+Na^+: 733.26114 [2*M*+Na]$^+$; found: 733.26925; elemental analysis calcd for $C_{18}H_{25}N_3S_2$: C 62.21, H 7.25, N 12.09, S 18.45; found: C 61.98, H 7.34, N 12.16, S 17.99.

6. X-ray crystal structure determinations

6.1. Experimental details

Data for compound **6b** were collected on a Bruker APEX II diffractometer using the Mo Kα radiation (λ = 0.71073 Å) from a fine focus sealed tube source at 100 K. Data for all the other compounds were collected on a Bruker D8 VENTURE PHOTON III-14 diffractometer using an Incoatec multilayer mirror monochromated with the Cu Kα radiation (λ = 1.54178 Å) from a microfocus sealed tube source at 100 K and with a detector resolution of 7.3910 pixels mm^{-1}. Computing data and reduction was made with the APEX 3 software [7]. Absorption corrections based on the multiscan method were applied [8]. All the structures were solved using SIR2004 [9]. They were refined by full-matrix, least-squares based on F^2 using SHELXL [10]. An empirical absorption correction was applied using SADABS [11]. All non-hydrogen atoms were anisotropically refined and the hydrogen atom positions were calculated and refined using a riding model.

Deposition Numbers 2130664–2130667 (for compounds **4a**, **4c**, **6b**, and **6e**, respectively) contain the supplementary crystallographic data for this paper. These data are provided free of charge by the joint Cambridge Crystallographic Data Centre and Fachinformationszentrum Karlsruhe Access Structures service www.ccdc.cam.ac.uk/structures.

6.2. Crystal data for CAAC-Mes-Cy·CS_2 (**4a**)

Orange-red needles with dimensions 0.08 × 0.08 × 0.06 mm obtained by slow diffusion of cyclohexane in THF at 6 °C, trigonal, *R*–3:*H*, *a* = 23.7061(3), *c* = 18.9250(3) Å, *V* = 9210.6(3) Å^3, *Z* = 18, μ = 2.35 mm^{-1}, reflections collected/unique = 42444/4185 (R_{int} = 0.075), final refinement converged with R^1 = 0.036 and wR^2 = 0.086 for all reflections, *GOF* = 1.02, $\Delta\rho_{max}/\Delta\rho_{min}$ = 0.31/−0.23 e·Å^{-3}.

6.3. Crystal data for CAAC-Die-MePh·CS_2 (**4c**)

Orange plates with dimensions 0.16 × 0.13 × 0.06 mm obtained by slow diffusion of cyclohexane in THF at 6 °C, monoclinic, $P2_1/c$, *a* = 13.3795(4), *b* = 9.7866(3), *c* = 16.6010(5) Å, β = 105.355(2)°, *V* = 2096.14(11) Å^3, *Z* = 4, μ = 2.35 mm^{-1}, reflections collected/unique = 32932/4277 (R_{int} = 0.053), final refinement converged with R^1 = 0.032 and wR^2 = 0.078 for all reflections, *GOF* = 1.05, $\Delta\rho_{max}/\Delta\rho_{min}$ = 0.33/–0.23 e·Å^{-3}.

6.4. Crystal data for MIC-Dip-Ph-Me·CS_2 (**6b**)

Orange prisms with dimensions 0.32 × 0.20 × 0.18 mm obtained by slow diffusion of petroleum ether in CD_2Cl_2 at −18 °C, monoclinic, $P2_1/n$, *a* = 6.9421(2), *b* = 12.7912(3), *c* = 23.4319(7) Å, β = 91.629(2)°, *V* = 2079.86(10) Å^3, *Z* = 4, μ = 0.27 mm^{-1}, reflections collected/unique = 48100/6335 (R_{int} = 0.032), final refinement converged with R^1 = 0.039 and wR^2 = 0.109 for all reflections, *GOF* = 1.04, $\Delta\rho_{max}/\Delta\rho_{min}$ = 0.59/−0.49 e·Å^{-3}.

6.5. Crystal data for MIC-Mes-Bu-Me·CS_2 (**6e**)

Dark red-brown blocky crystals with dimensions 0.14 × 0.03 × 0.01 mm obtained by slow diffusion of *n*-hexane in $CDCl_3$ at 6 °C, monoclinic, $P2_1/c$, *a* = 6.6167(4), *b* = 12.1170(6), *c* = 22.420(2) Å, β = 93.361(4)°, *V* = 1794.4(2) Å^3, *Z* = 4, μ = 0.30 mm^{-1}, reflections collected/unique = 28369/3668 (R_{int} = 0.044), final refinement converged with R^1 = 0.036 and wR^2 = 0.096 for all reflections, *GOF* = 1.05, $\Delta\rho_{max}/\Delta\rho_{min}$ = 0.29/−0.25 e·Å^{-3}.

7. References

[1] D. Brown, N. Sanguantrakun, B. Schulze, U. Schubert, C. Berlinguette, *J. Am. Chem. Soc.* **2012**, *134*, 12354–12357.

[2] D. Kumar, V. B. Reddy, *Synthesis* **2010**, 1687–1691.

[3] C. Johnson, M. Albrecht, *Organometallics* **2017**, *36*, 2902–2913.

[4] G. Guisado-Barrios, J. Bouffard, B. Donnadieu, G. Bertrand, *Angew. Chem., Int. Ed*. **2010**, *49*, 4759–4762.

[5] J. Lorkowski, P. Żak, M. Kubicki, C. Pietraszuk, D. Jędrzkiewicz, J. Ejfler, *New J. Chem.* **2018**, *42*, 10134–10141.

[6] L. Hettmanczyk, D. Schulze, L. Suntrup, B. Sarkar, *Organometallics* **2016**, *35*, 3828–3836.

[7] Bruker, APEX 3, Madison, WI, USA, **2005**.

[8] R. C. Clark, J. S. Reid, *Acta Crystallogr., Sect. A: Fundam. Crystallogr.* **1995**, *51*, 887–897.

[9] M. C. Burla, R. Caliandro, M. Camalli, B. Carrozzini, G. L. Cascarano, L. De Caro, C. Giacovazzo, G. Polidori, R. Spagna, *J. Appl. Crystallogr.* **2005**, *38*, 381–388.

[10] G. M. Sheldrick, SHELX-97 (SHELXS 97 and SHELXL 97), Programs for Crystal Structure Analyses, University of Göttingen: Göttingen (Germany), **1998**.

[11] G. M. Sheldrick, SADABS, Programs for Scaling and Correction of Area Detection Data, University of Göttingen: Göttingen (Germany), **1996**.

8. NMR Spectra

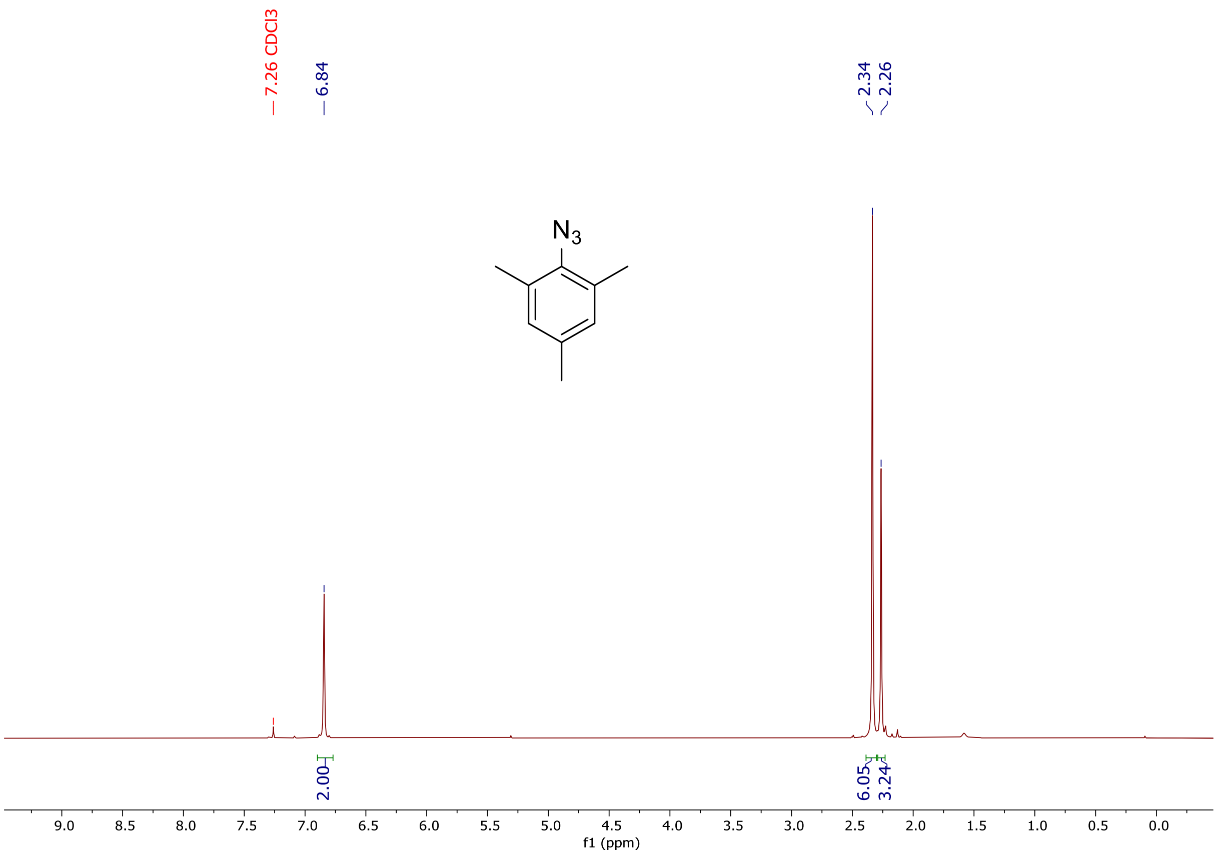

Figure S1. ^{1}H NMR spectrum (400 MHz, $CDCl_3$, 298 K) of 2-azido-1,3,5-trimethylbenzene.

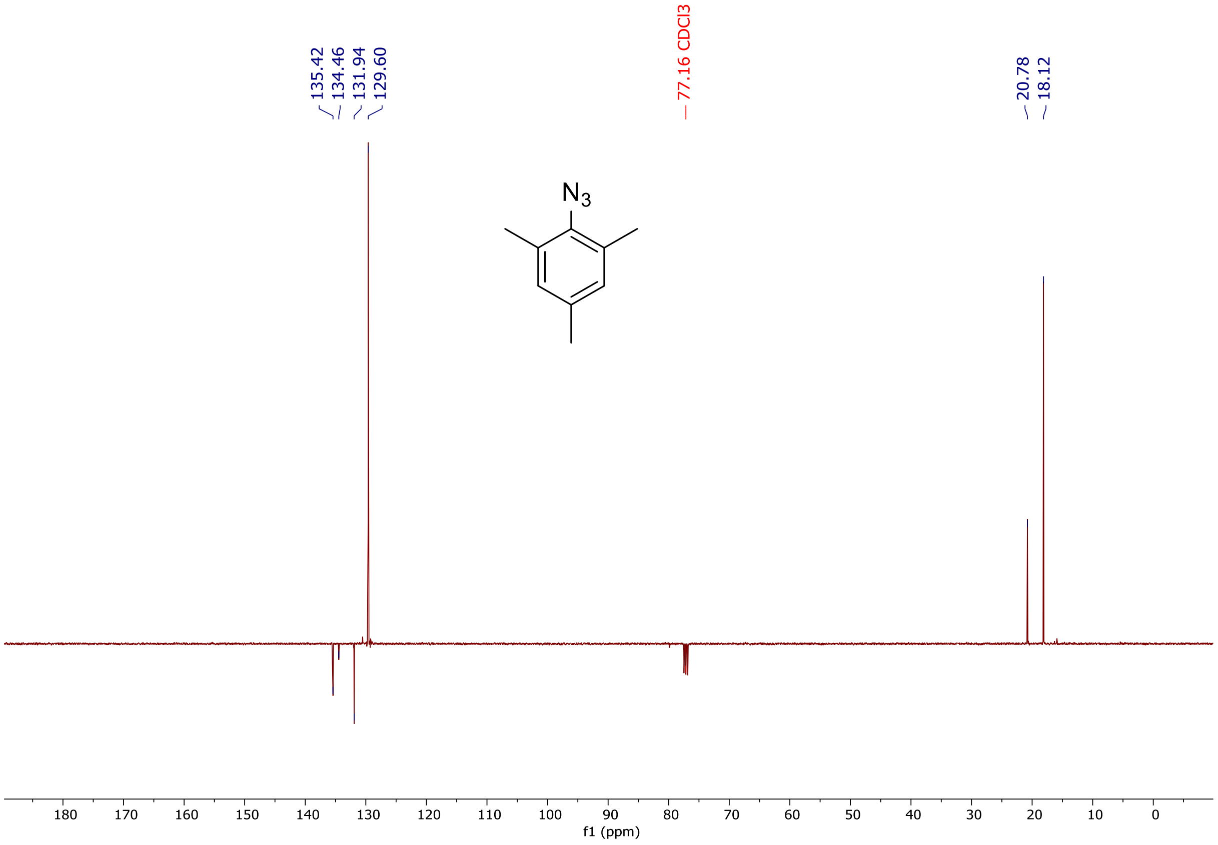

Figure S2. $^{13}C\{^{1}H\}$ APT NMR spectrum (100 MHz, $CDCl_3$, 298 K) of 2-azido-1,3,5-trimethylbenzene.

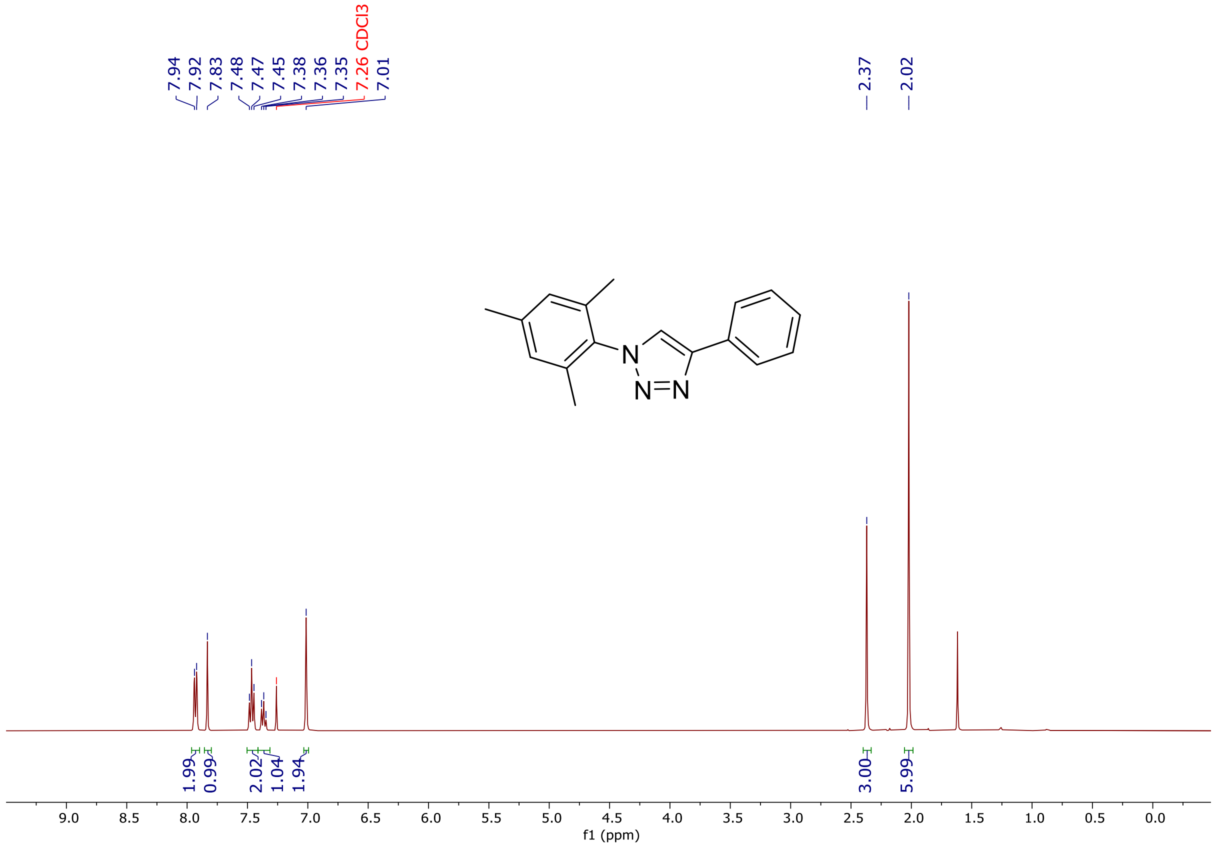

Figure S3. ^{1}H NMR spectrum (400 MHz, $CDCl_3$, 298 K) of 1-mesityl-4-phenyl-1*H*-1,2,3-triazole.

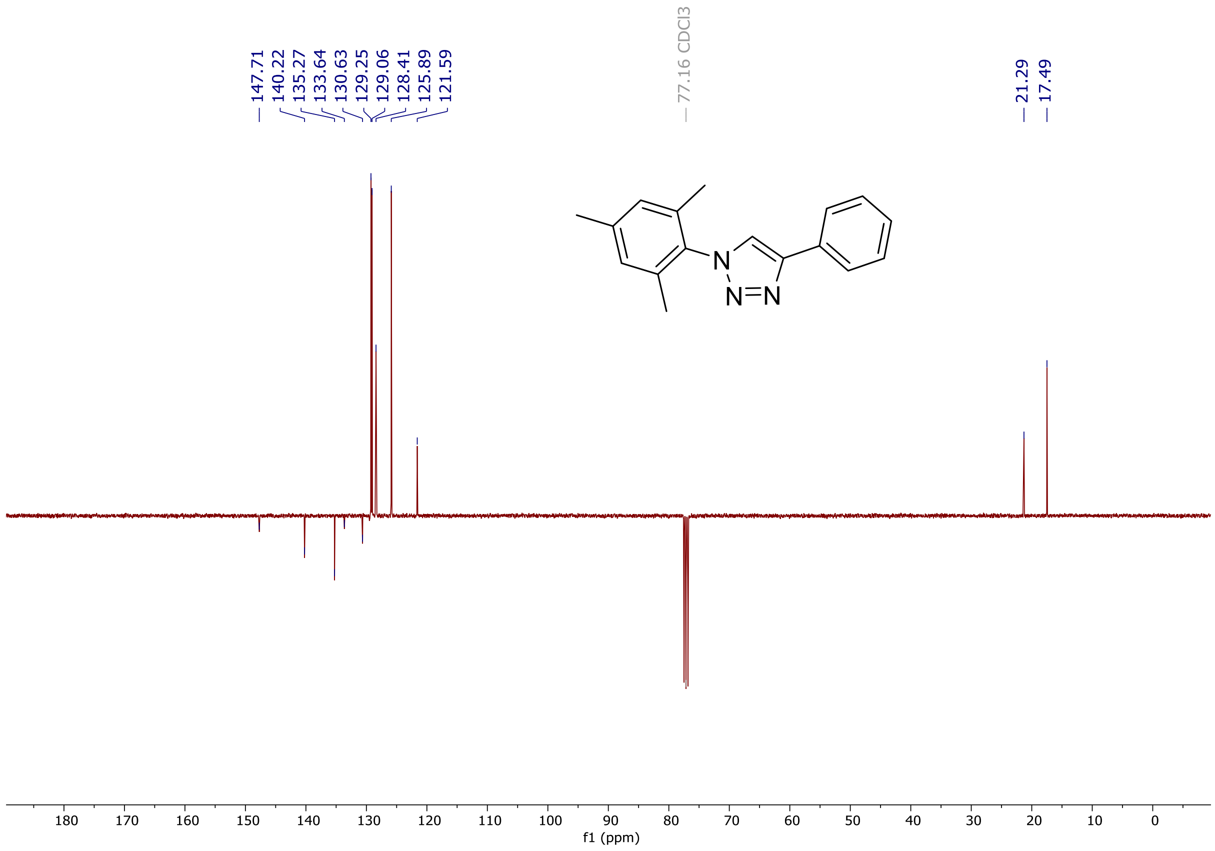

Figure S4. $^{13}C\{^{1}H\}$ APT NMR spectrum (100 MHz, $CDCl_3$, 298 K) of 1-mesityl-4-phenyl-1*H*-1,2,3-triazole.

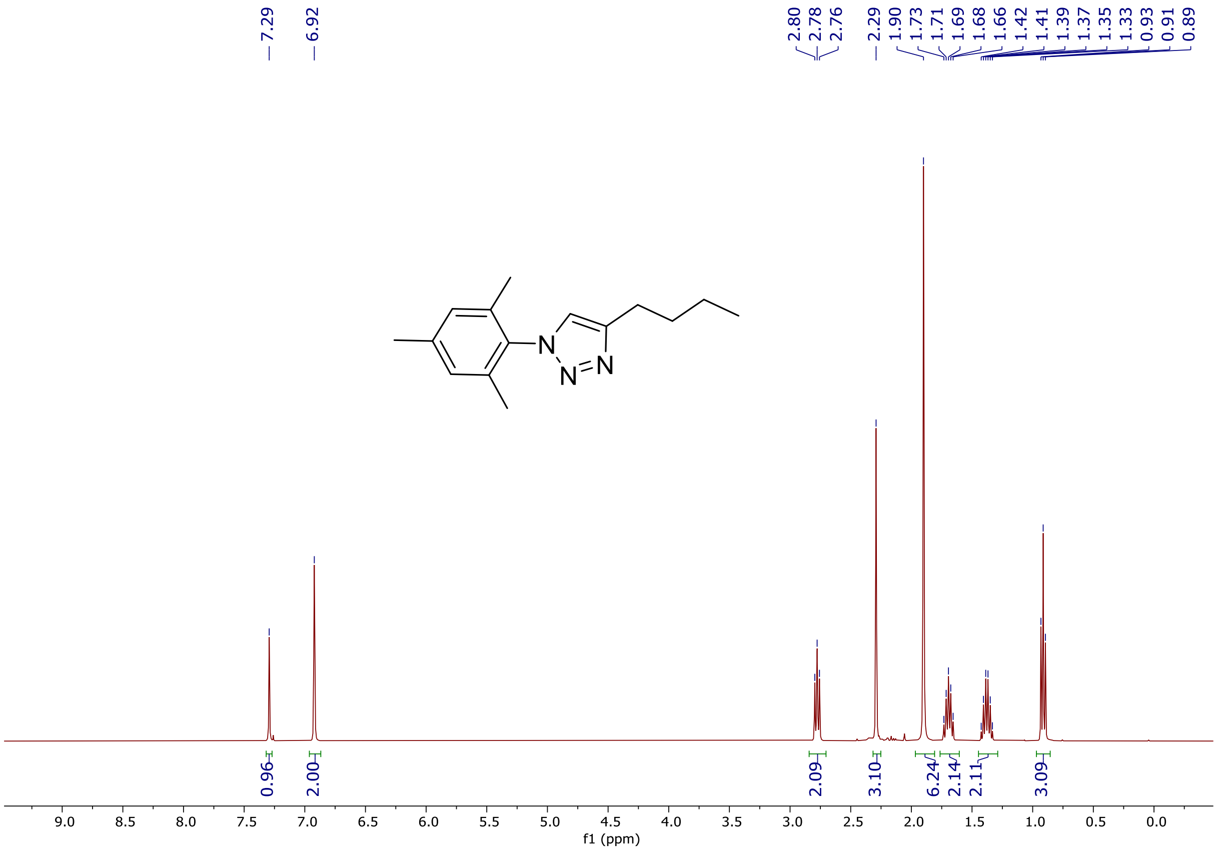

Figure S5. ^{1}H NMR spectrum (400 MHz, $CDCl_3$, 298 K) of 4-butyl-1-mesityl-1*H*-1,2,3-triazole.

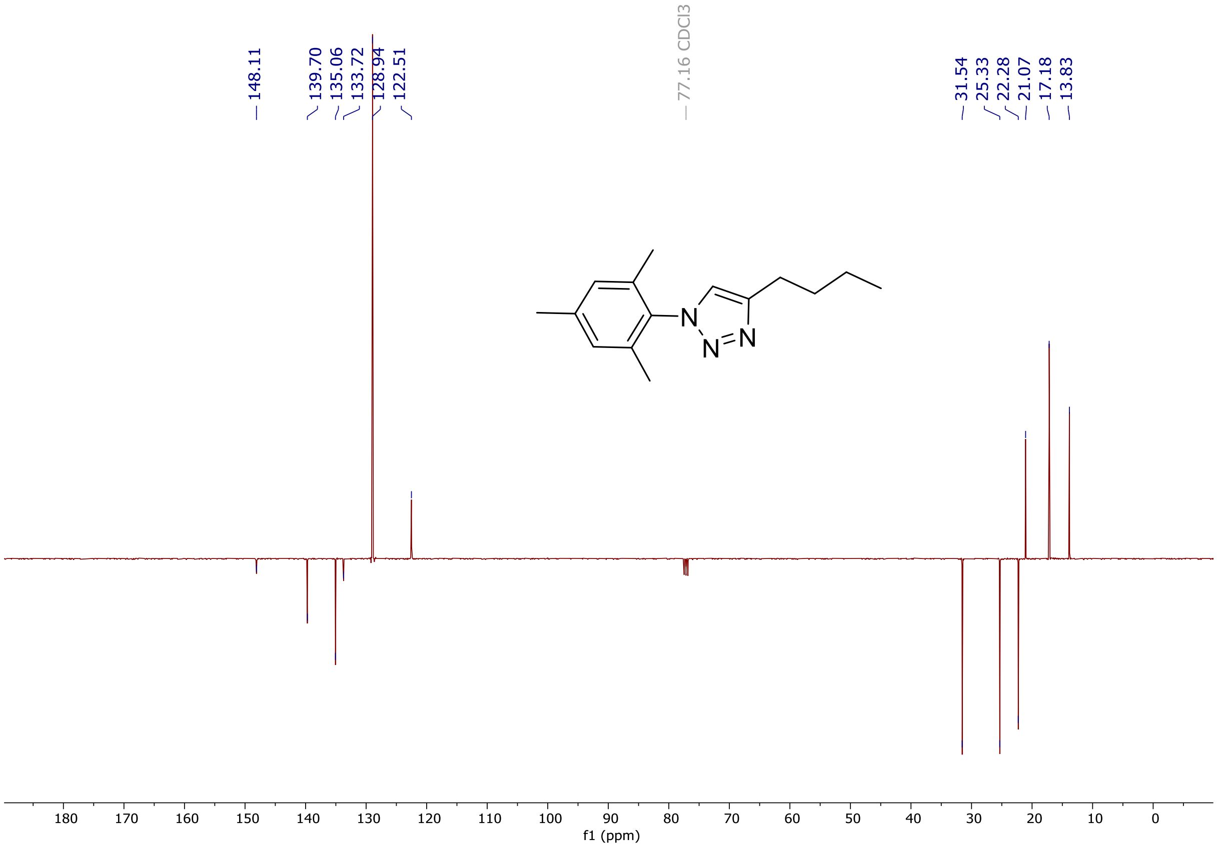

Figure S6. $^{13}C\{^{1}H\}$ APT NMR spectrum (100 MHz, $CDCl_3$, 298 K) of 4-butyl-1-mesityl-1*H*-1,2,3-triazole.

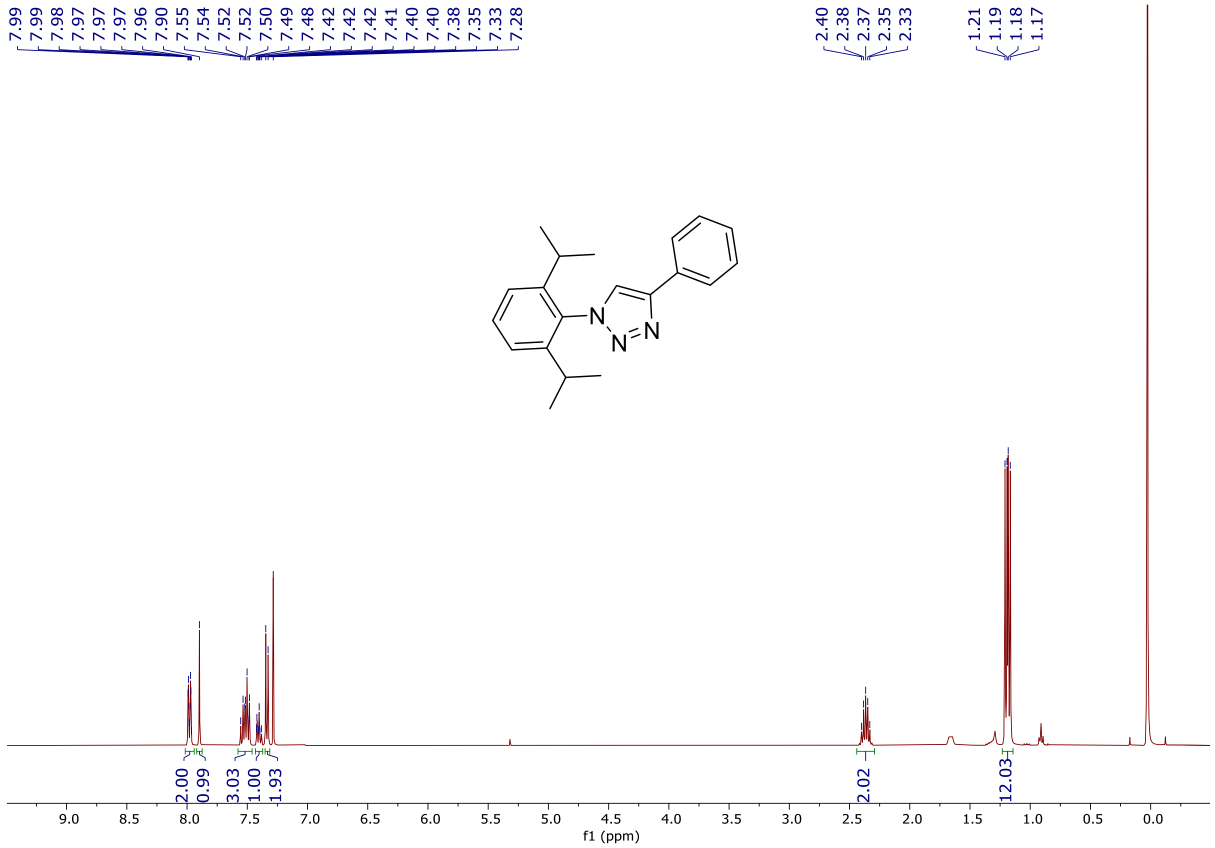

Figure S7. ^{1}H NMR spectrum (400 MHz, $CDCl_3$, 298 K) of 1-(2,6-diisopropylphenyl)-4-phenyl-1*H*-1,2,3-triazole.

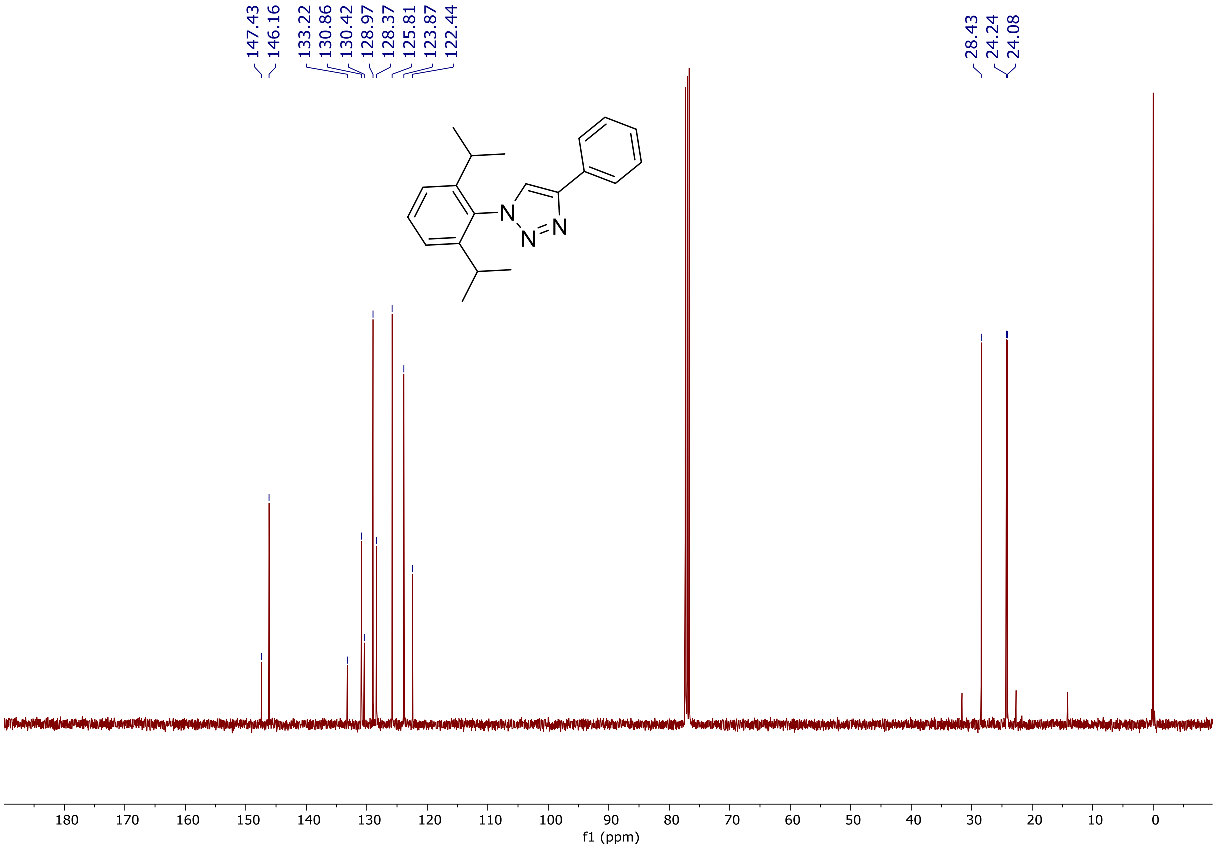

Figure S8. $^{13}C\{^{1}H\}$ NMR spectrum (100 MHz, $CDCl_3$, 298 K) of 1-(2,6-diisopropylphenyl)-4-phenyl-1*H*-1,2,3-triazole.

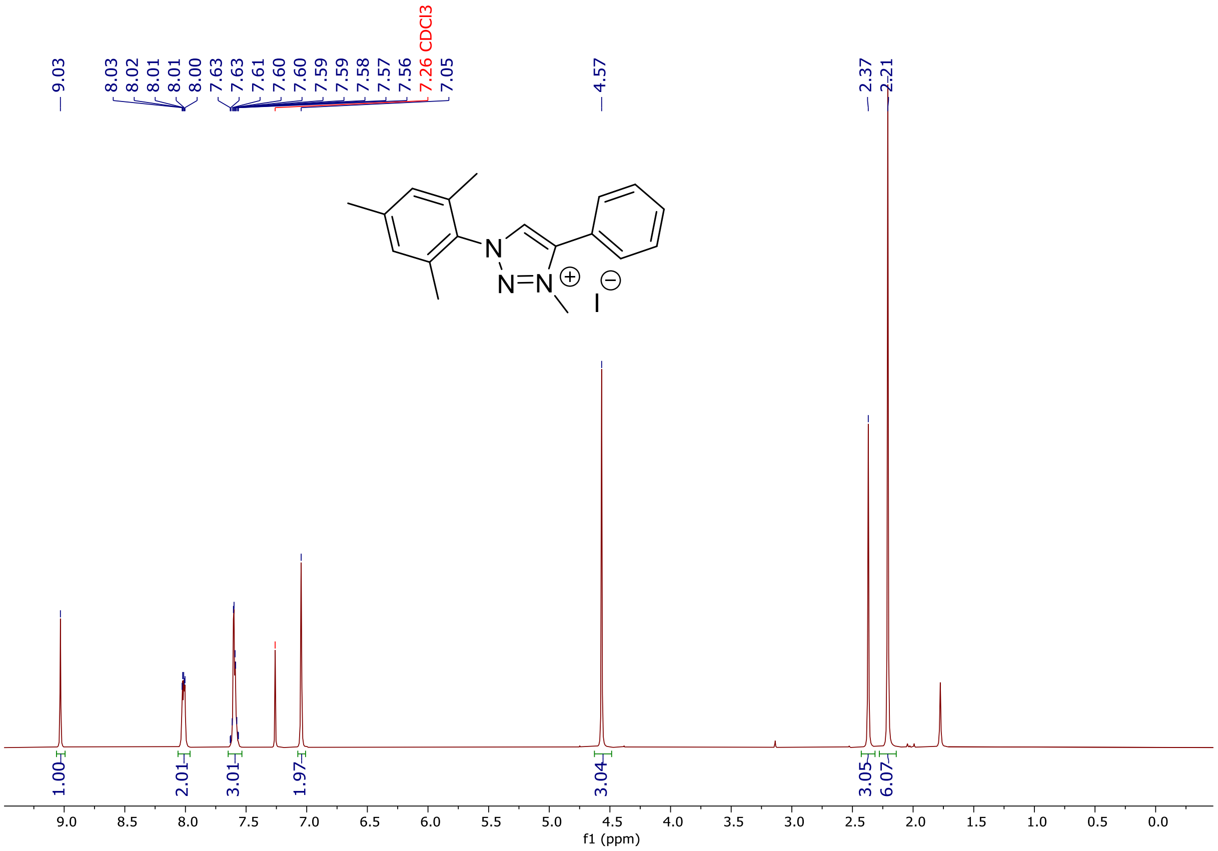

Figure S9. ^{1}H NMR spectrum (400 MHz, $CDCl_3$, 298 K) of 1-mesityl-3-methyl-4-phenyl-1*H*-1,2,3-triazol-3-ium iodide (**5a**).

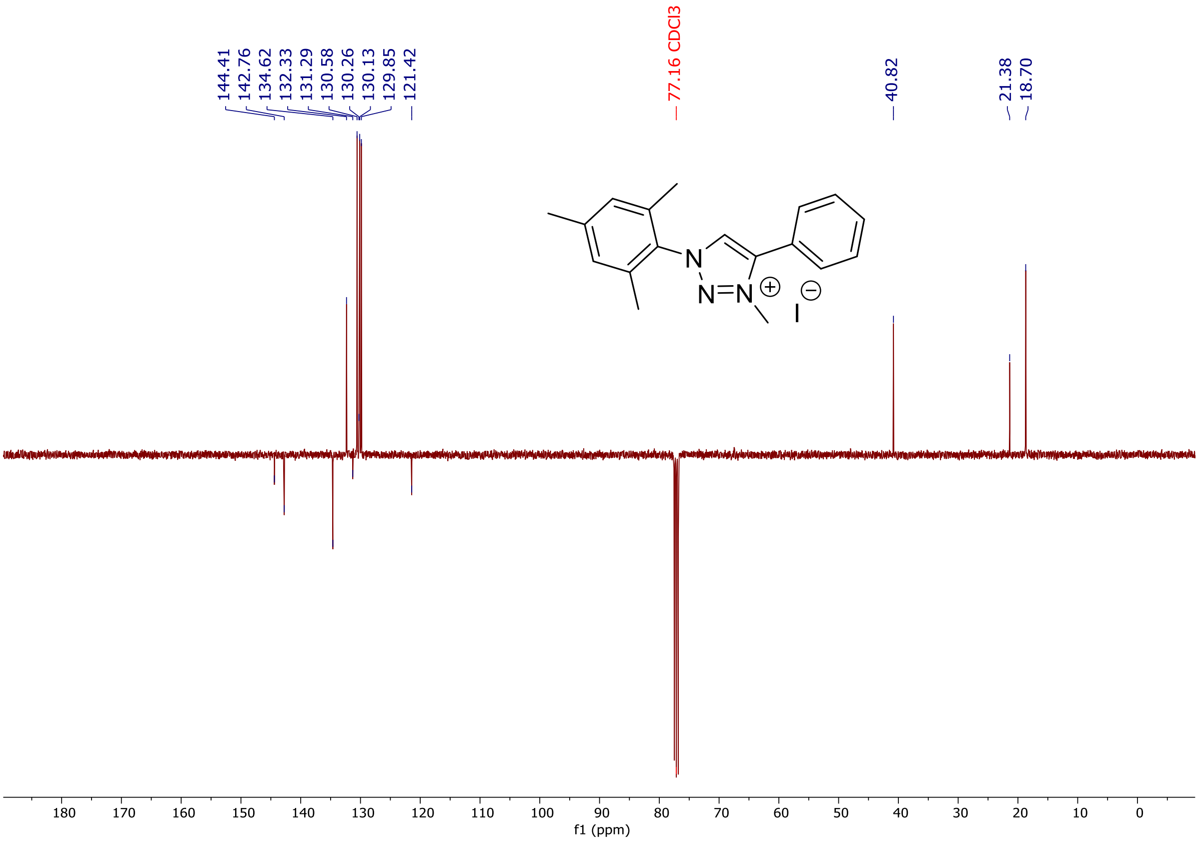

Figure S10. $^{13}C\{^{1}H\}$ APT NMR spectrum (100 MHz, $CDCl_3$, 298 K) of 1-mesityl-3-methyl-4-phenyl-1*H*-1,2,3-triazol-3-ium iodide (**5a**).

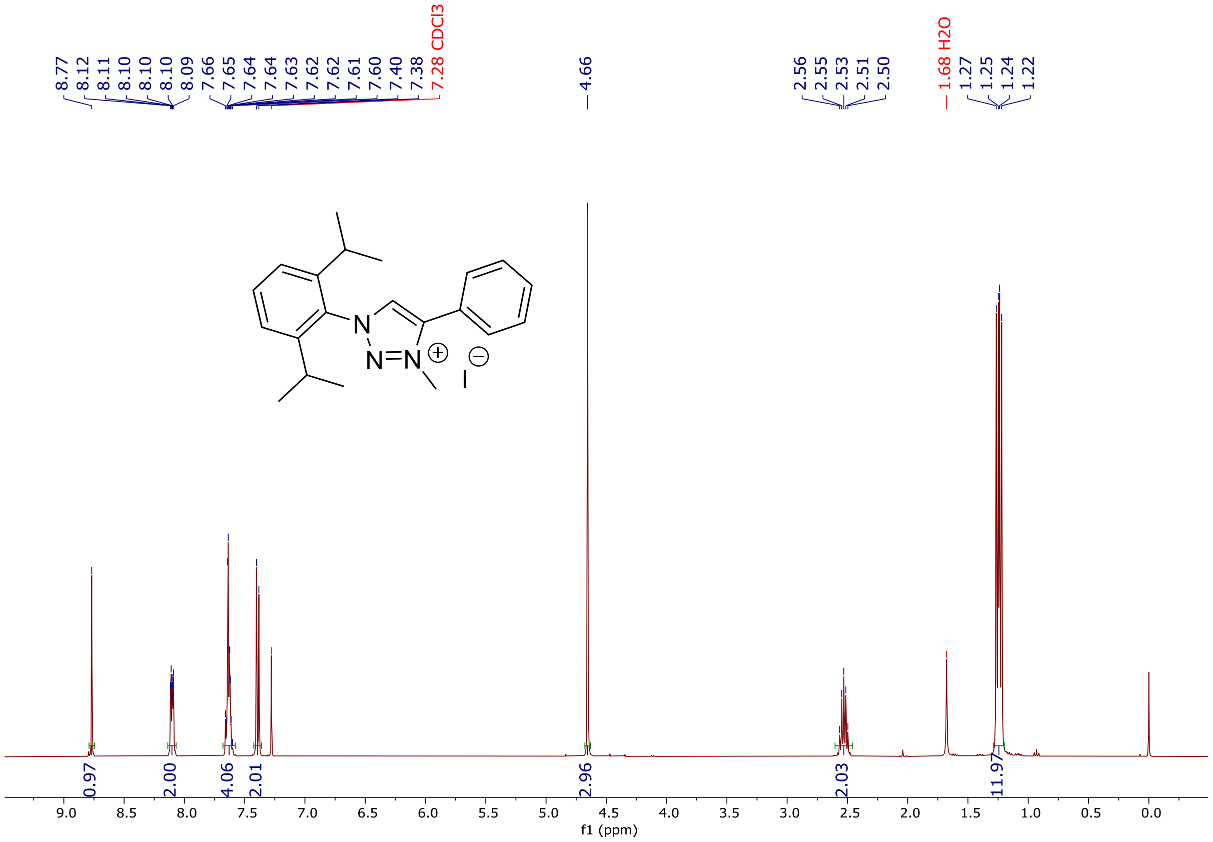

Figure S11. ^{1}H NMR spectrum (400 MHz, $CDCl_3$, 298 K) of 1-(2,6-diisopropylphenyl)-3-methyl-4-phenyl-1*H*-1,2,3-triazol-3-ium iodide (**5b**).

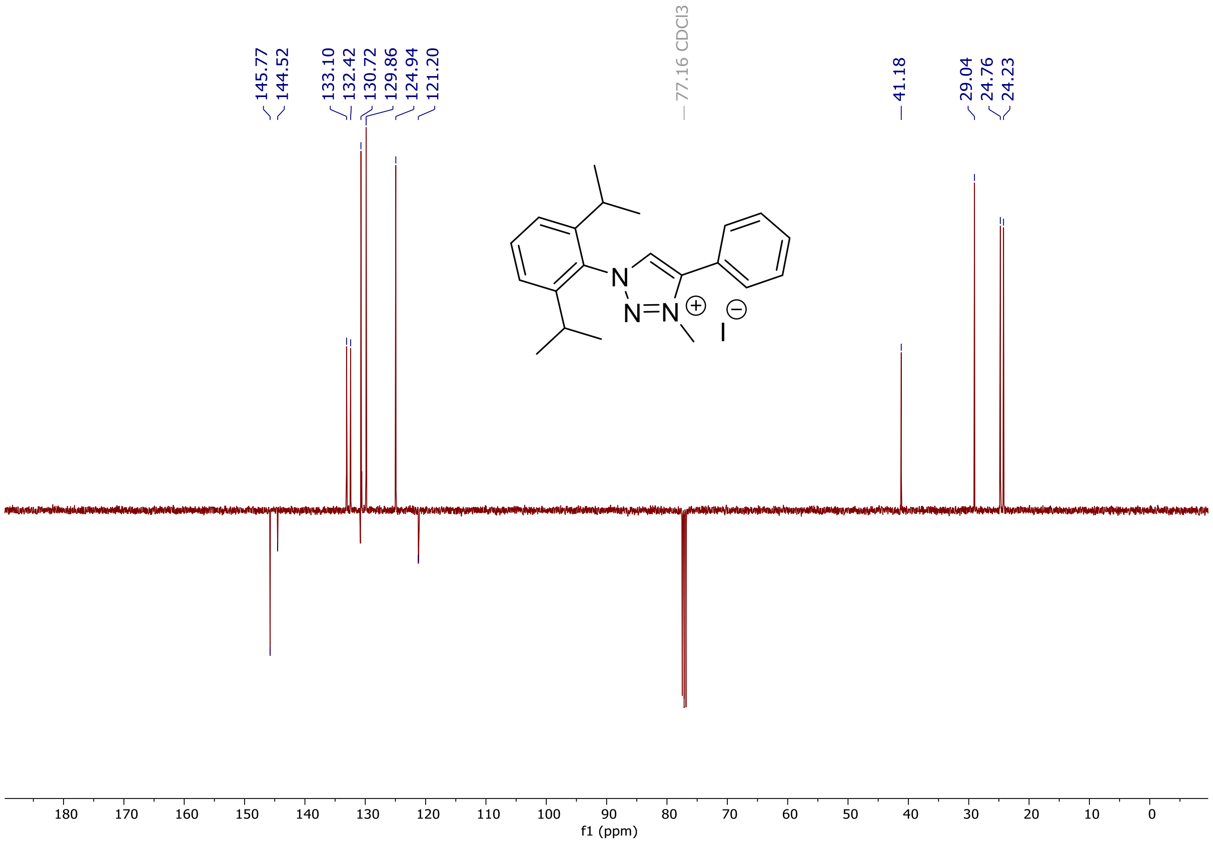

Figure S12. ^{13}C{^{1}H} APT NMR spectrum (100 MHz, $CDCl_3$, 298 K) of 1-(2,6-diisopropylphenyl)-3-methyl-4-phenyl-1*H*-1,2,3-triazol-3-ium iodide (**5b**).

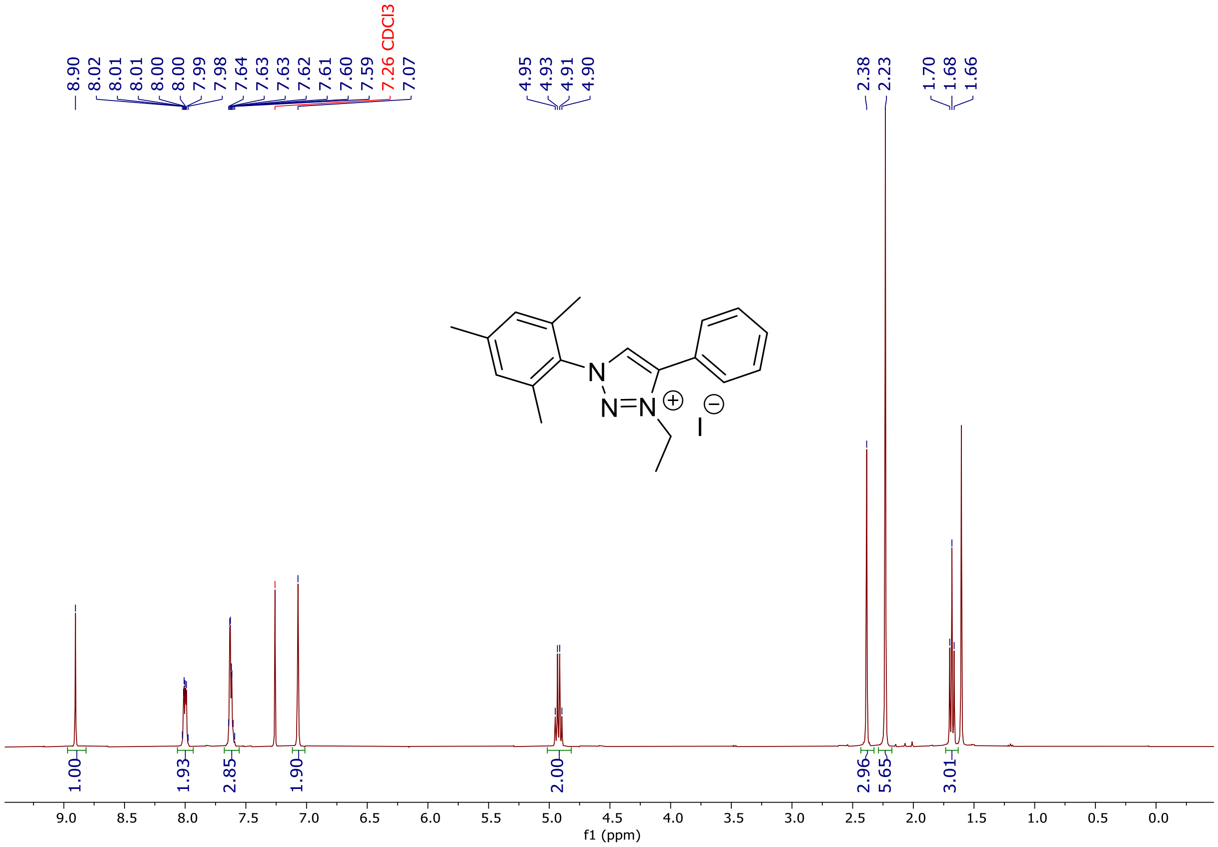

Figure S13. ^{1}H NMR spectrum (400 MHz, $CDCl_3$, 298 K) of 3-ethyl-1-mesityl-4-phenyl-1*H*-1,2,3-triazol-3-ium iodide (**5c**).

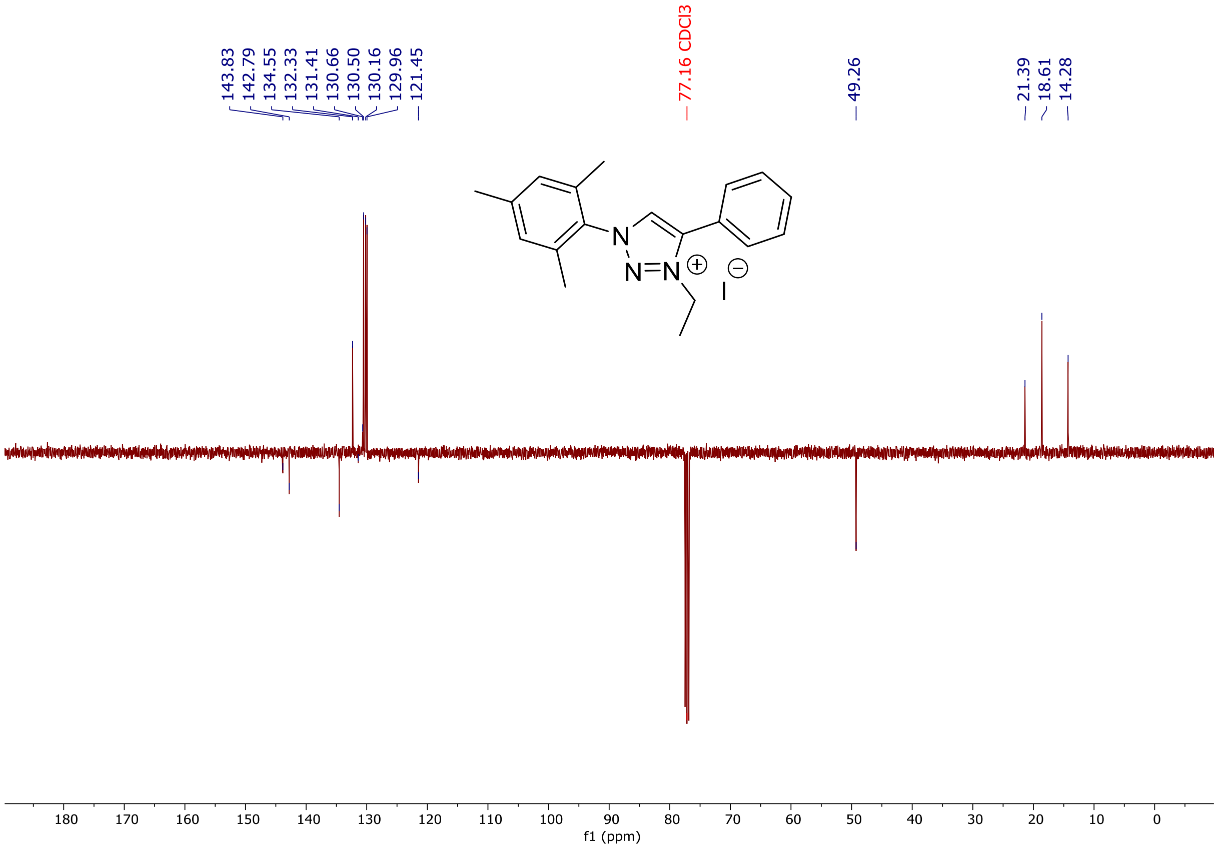

Figure S14. $^{13}C\{^{1}H\}$ APT NMR spectrum (100 MHz, $CDCl_3$, 298 K) of 3-ethyl-1-mesityl-4-phenyl-1*H*-1,2,3-triazol-3-ium iodide (**5c**).

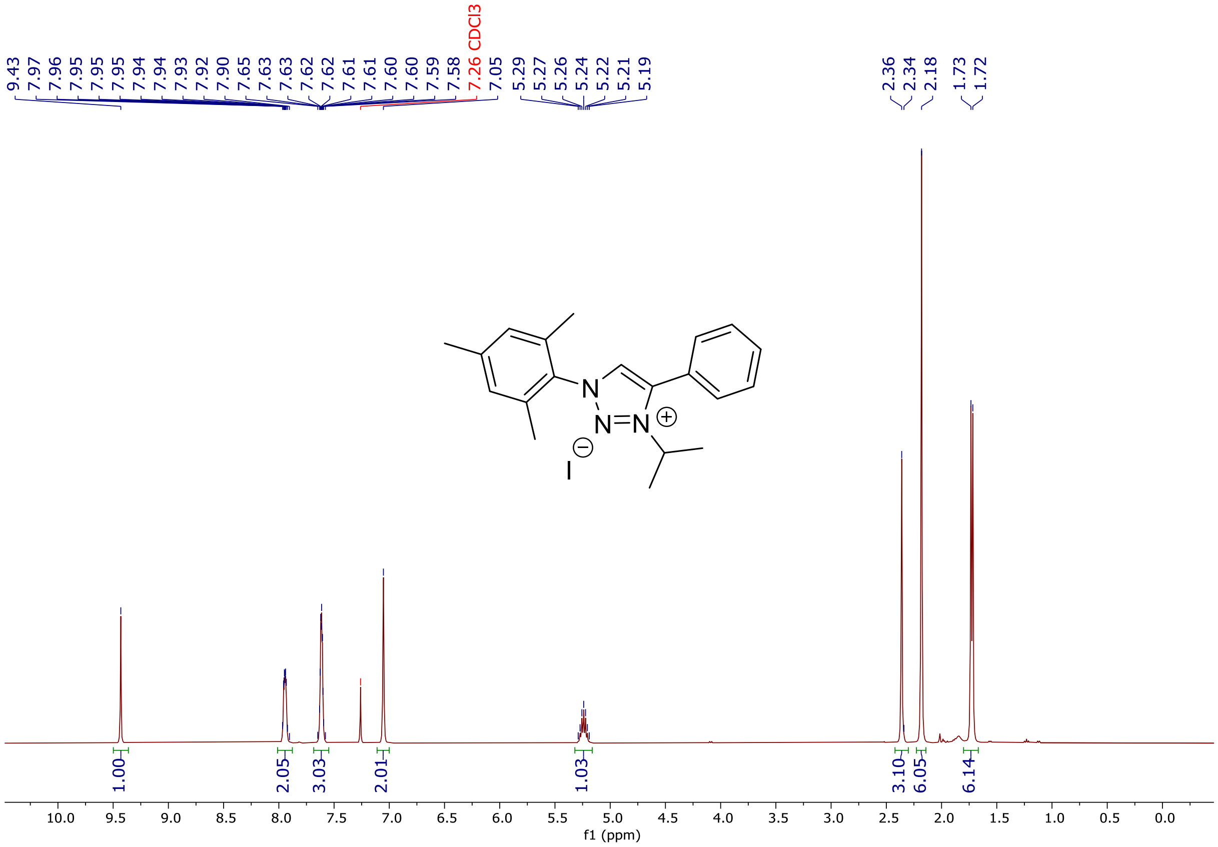

Figure S15. ^{1}H NMR spectrum (400 MHz, $CDCl_3$, 298 K) of 3-isopropyl-1-mesityl-4-phenyl-1H-1,2,3-triazol-3-ium iodide (**5d**).

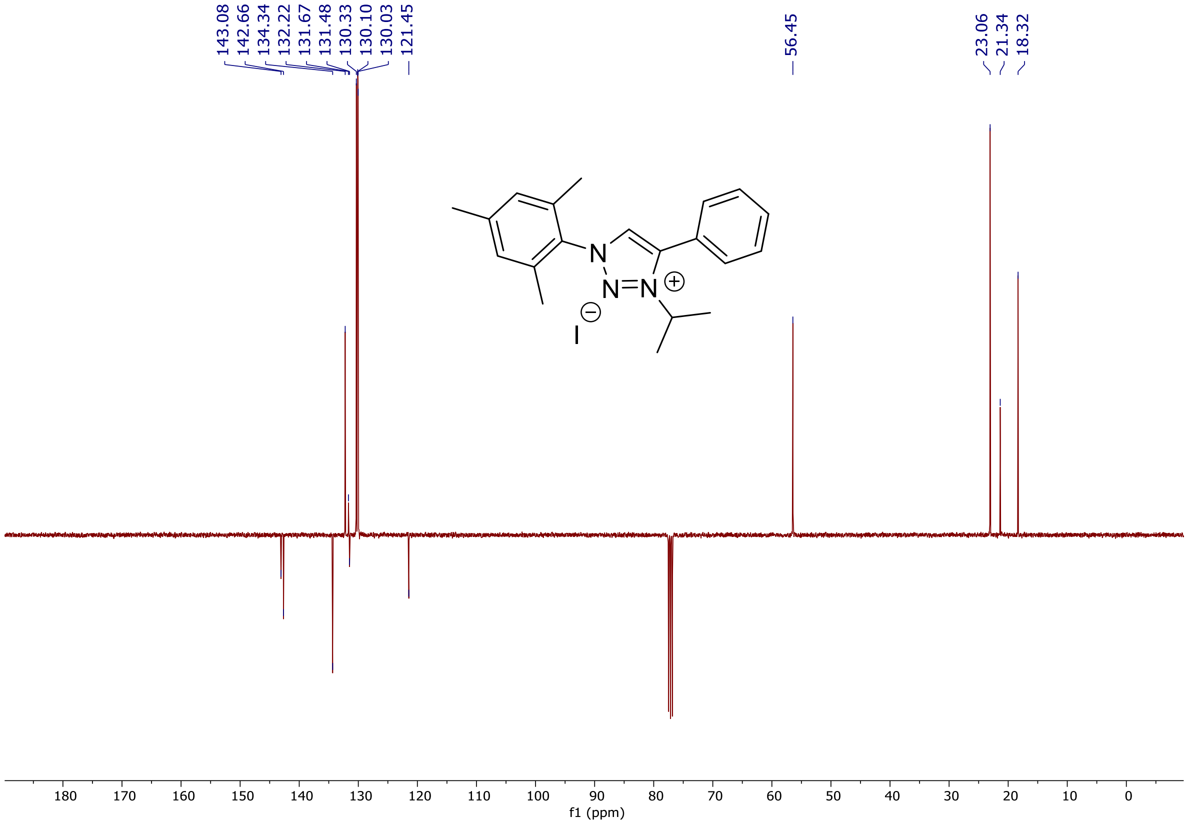

Figure S16. $^{13}C\{^{1}H\}$ APT NMR spectrum (100 MHz, $CDCl_3$, 298 K) of 3-isopropyl-1-mesityl-4-phenyl-1*H*-1,2,3-triazol-3-ium iodide (**5d**).

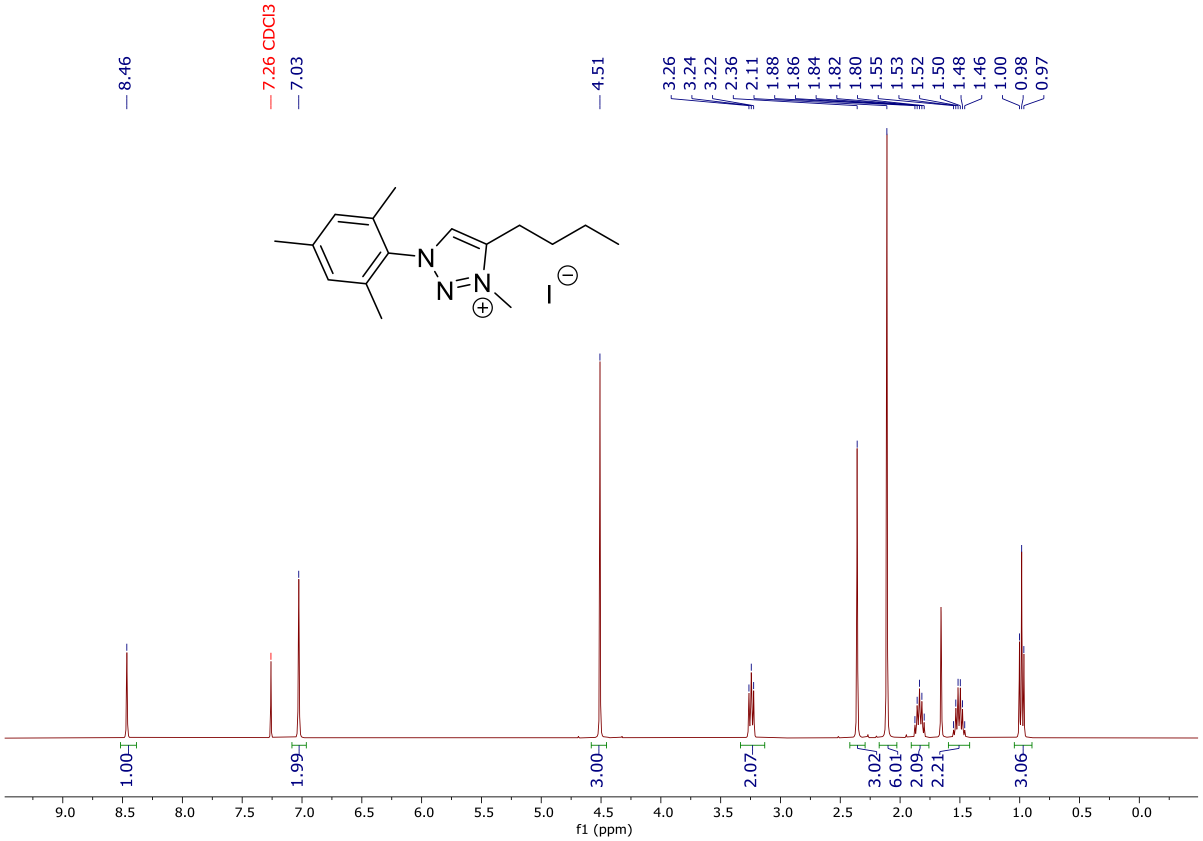

Figure S17. ^{1}H NMR spectrum (400 MHz, $CDCl_3$, 298 K) of 4-butyl-1-mesityl-3-methyl-1*H*-1,2,3-triazol-3-ium iodide (**5e**).

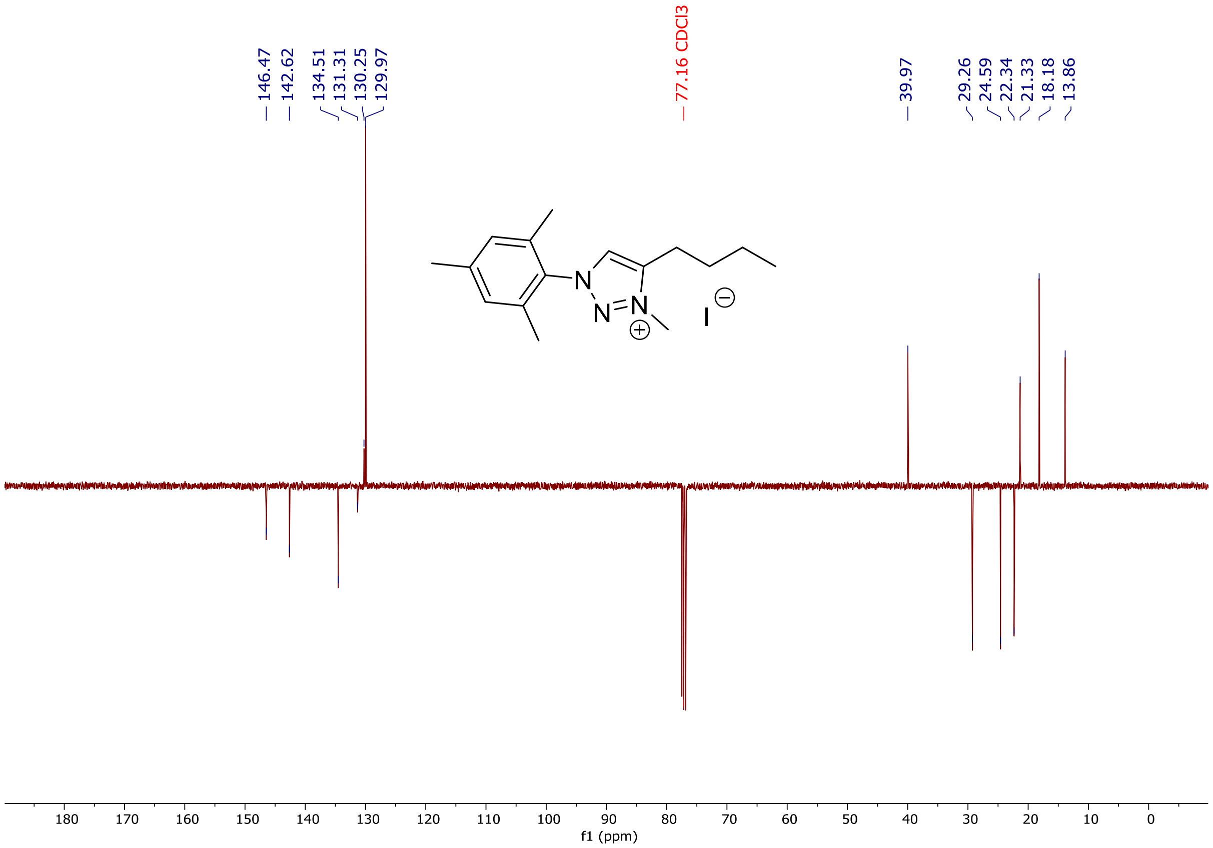

Figure S18. $^{13}C\{^{1}H\}$ APT NMR spectrum (100 MHz, $CDCl_3$, 298 K) of 4-butyl-1-mesityl-3-methyl-1*H*-1,2,3-triazol-3-ium iodide (**5e**).

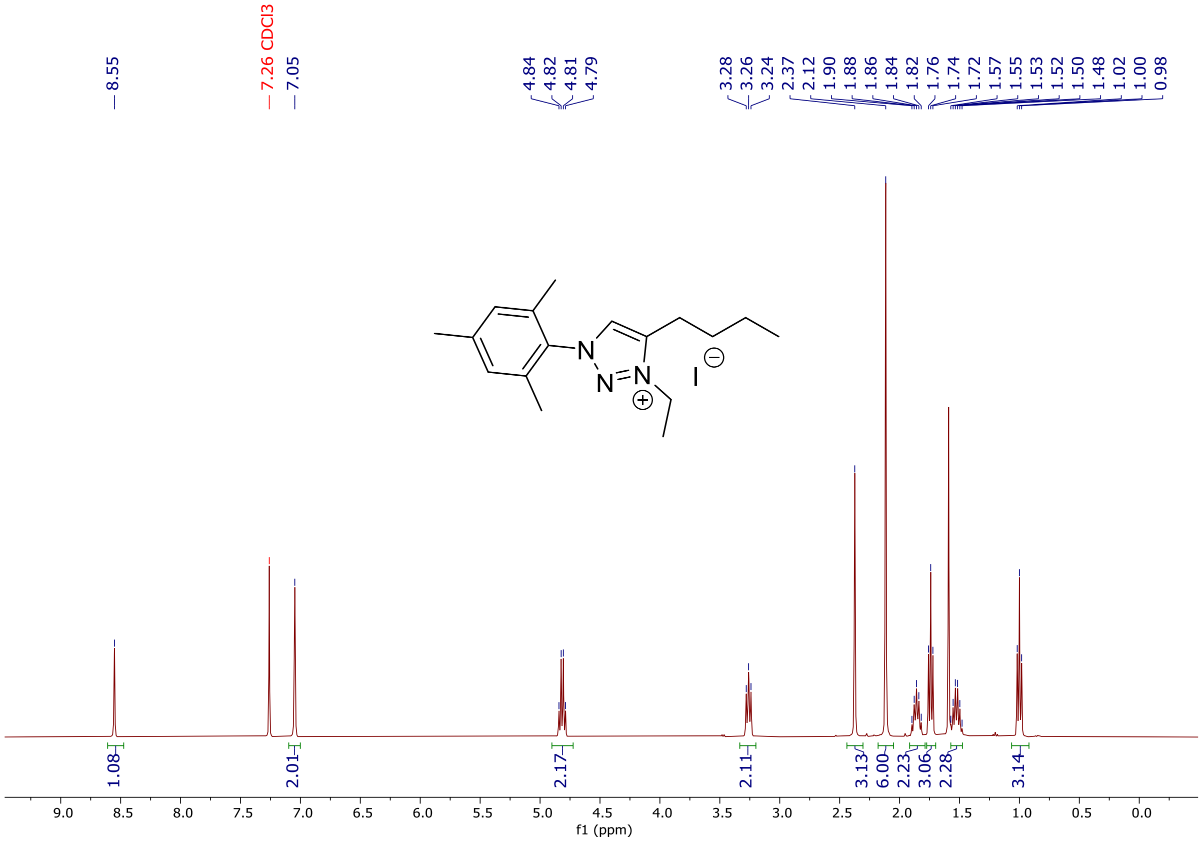

Figure S19. ^{1}H NMR spectrum (400 MHz, $CDCl_3$, 298 K) of 4-butyl-3-ethyl-1-mesityl-1*H*-1,2,3-triazol-3-ium iodide (**5f**).

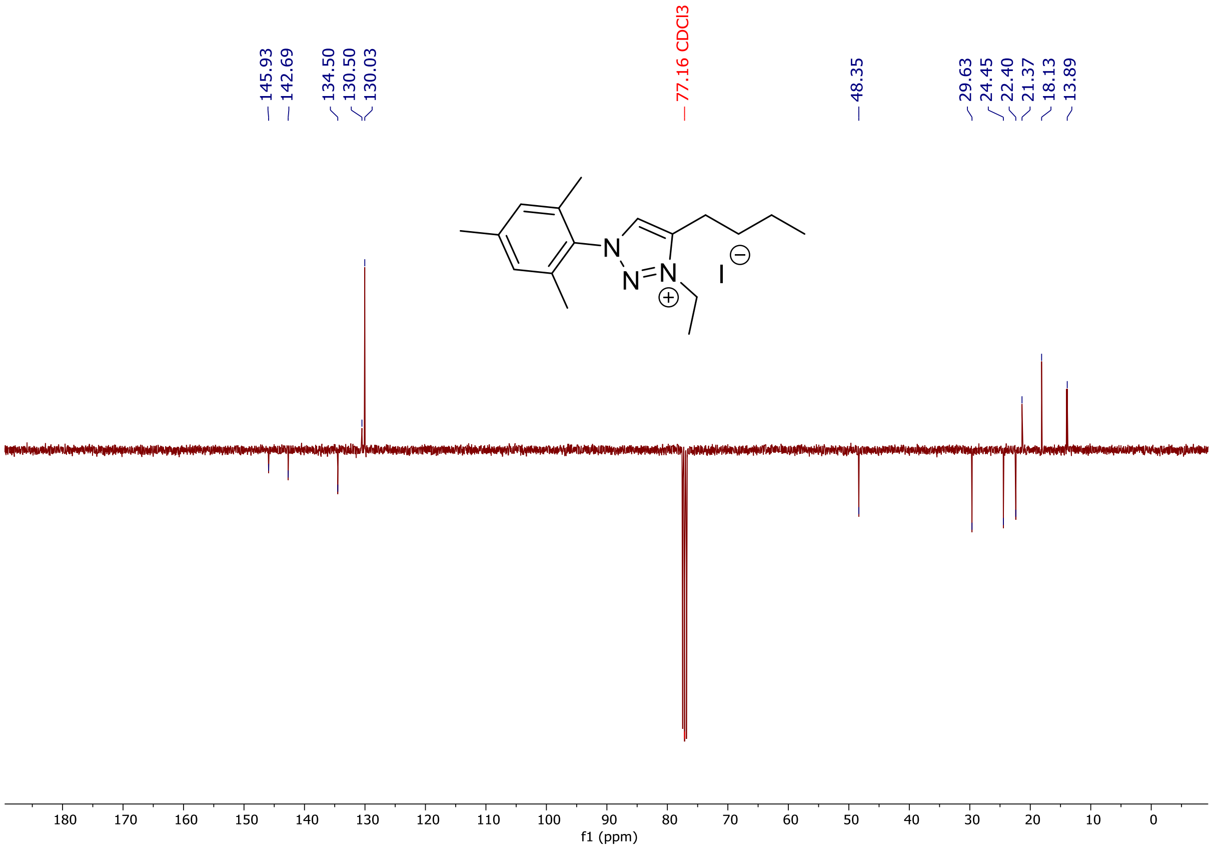

Figure S20. $^{13}C\{^1H\}$ APT NMR spectrum (100 MHz, $CDCl_3$, 298 K) of 4-butyl-3-ethyl-1-mesityl-1*H*-1,2,3-triazol-3-ium iodide (**5f**).

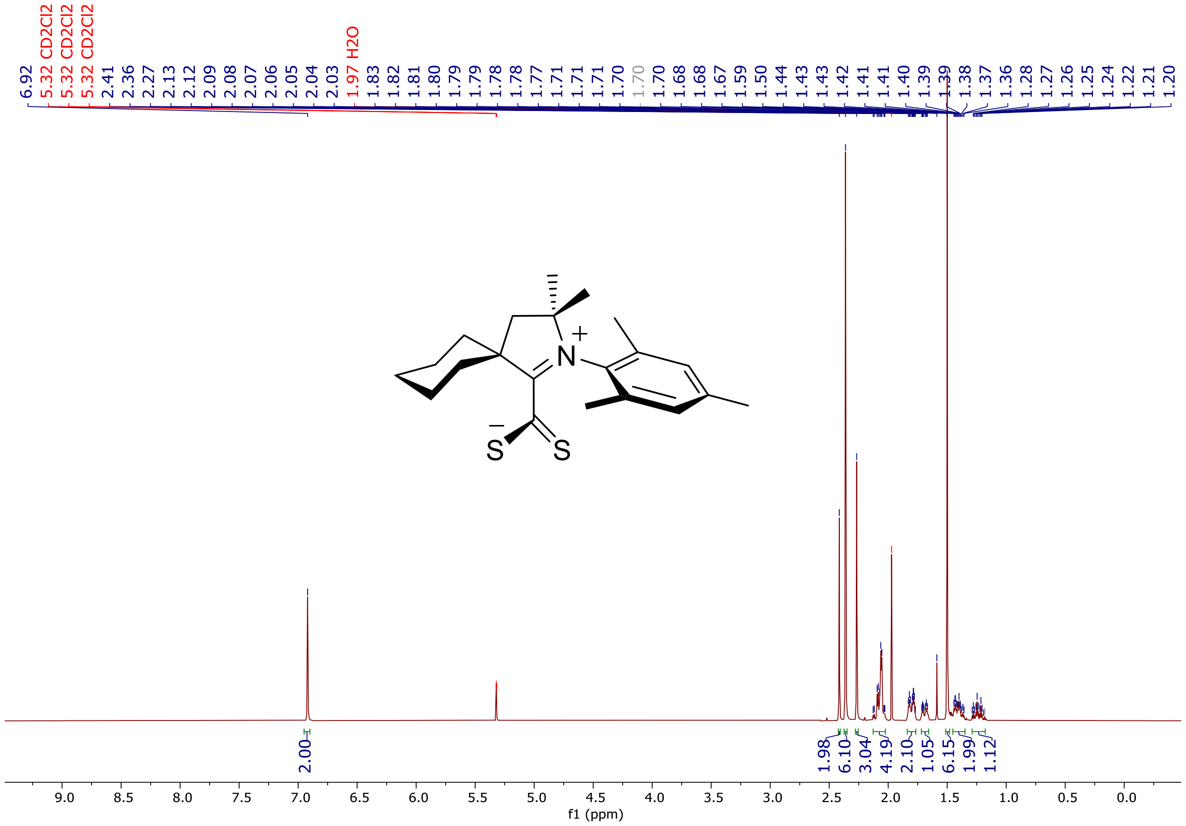

Figure S21. ^{1}H NMR spectrum (400 MHz, CD_2Cl_2, 298 K) of 2-mesityl-3,3-dimethyl-2-azaspiro[4.5]dec-1-en-2-ium-1-dithiocarboxylate (**4a**).

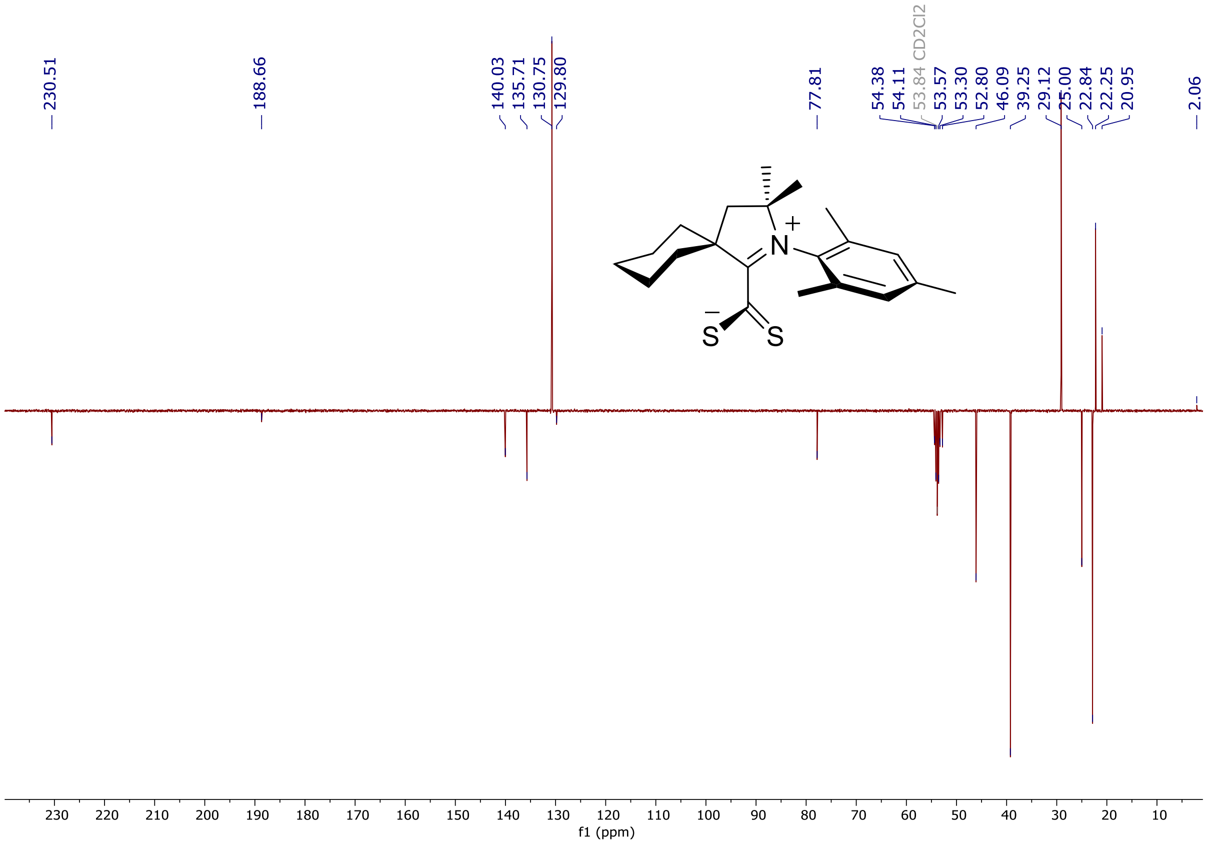

Figure S22. $^{13}C\{^{1}H\}$ APT NMR spectrum (100 MHz, CD_2Cl_2, 298 K) of 2-mesityl-3,3-dimethyl-2-azaspiro[4.5]dec-1-en-2-ium-1-dithiocarboxylate (**4a**).

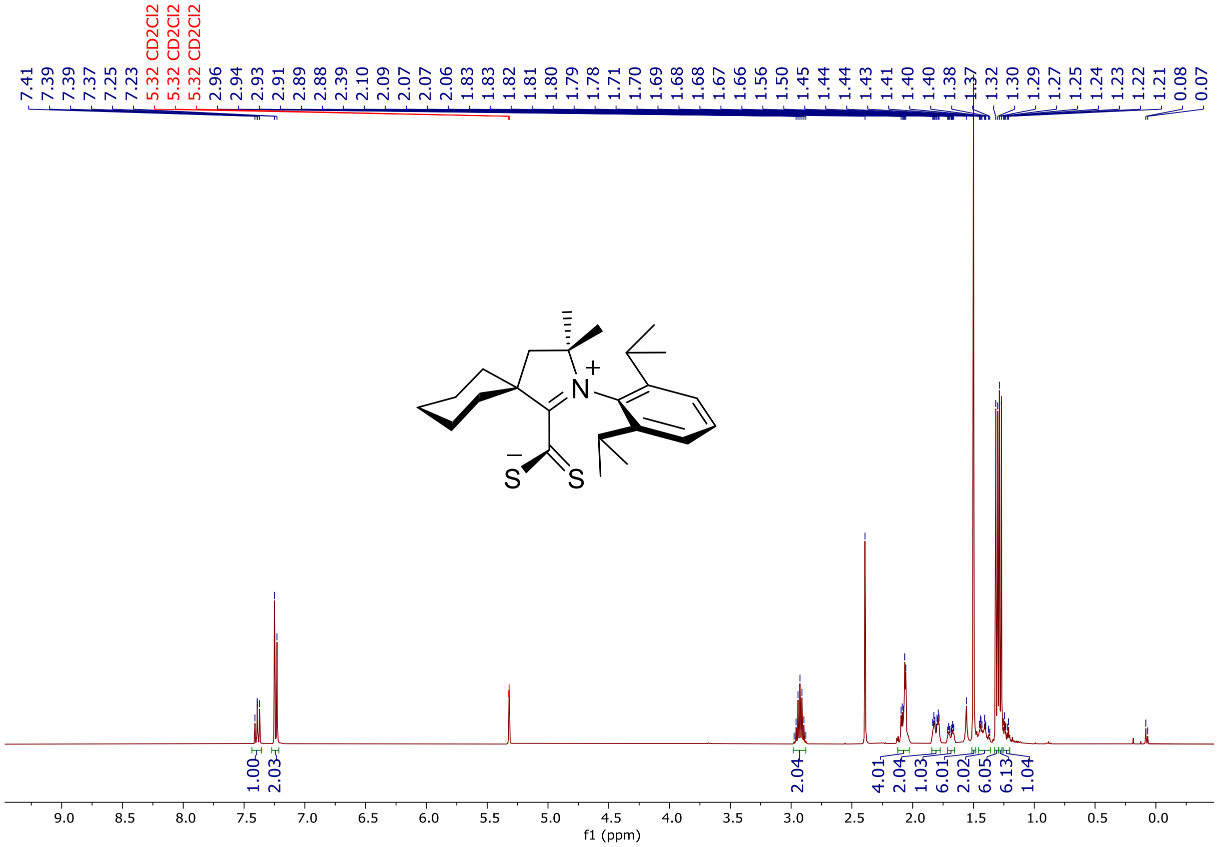

Figure S23. ^{1}H NMR spectrum (400 MHz, CD_2Cl_2, 298 K) of 2-(2,6-diisopropylphenyl)-3,3-dimethyl-2-azaspiro[4.5]dec-1-en-2-ium-1-dithiocarboxylate (**4b**).

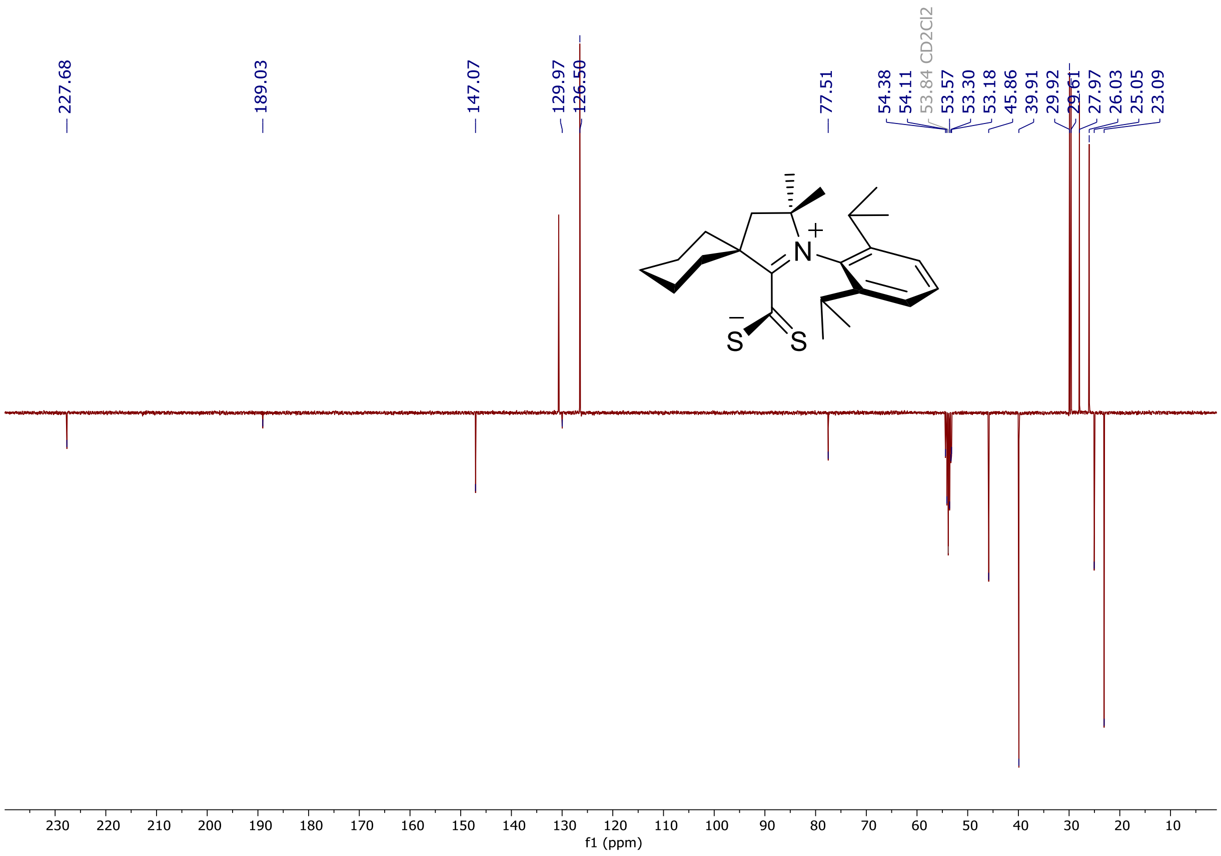

Figure S24. $^{13}C\{^1H\}$ APT NMR spectrum (100 MHz, CD_2Cl_2, 298 K) of 2-(2,6-diisopropylphenyl)-3,3-dimethyl-2-azaspiro[4.5]dec-1-en-2-ium-1-dithiocarboxylate (**4b**).

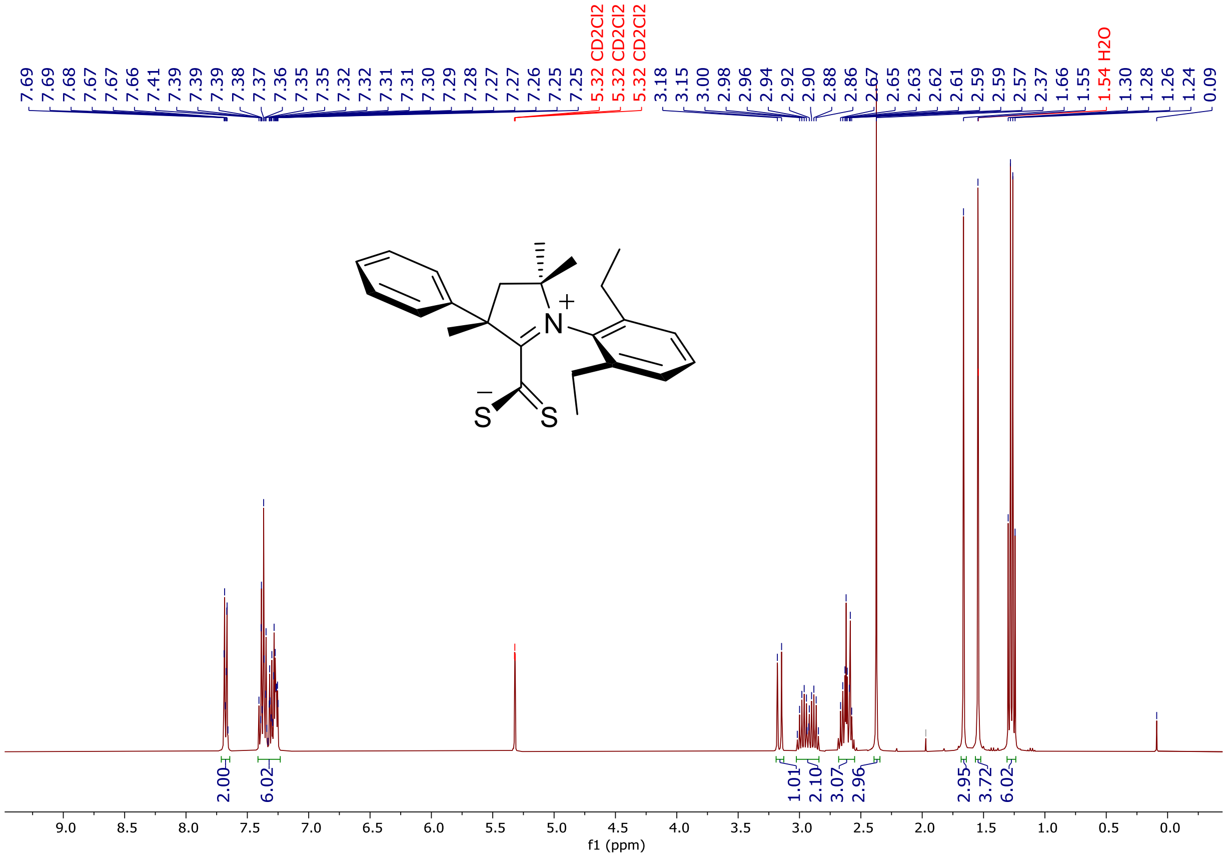

Figure S25. ^{1}H NMR spectrum (400 MHz, CD_2Cl_2, 298 K) of 1-(2,6-diethylphenyl)-2,2,4-trimethyl-4-phenyl-3,4-dihydro-2H-pyrrol-1-ium-5-dithiocarboxylate (**4c**).

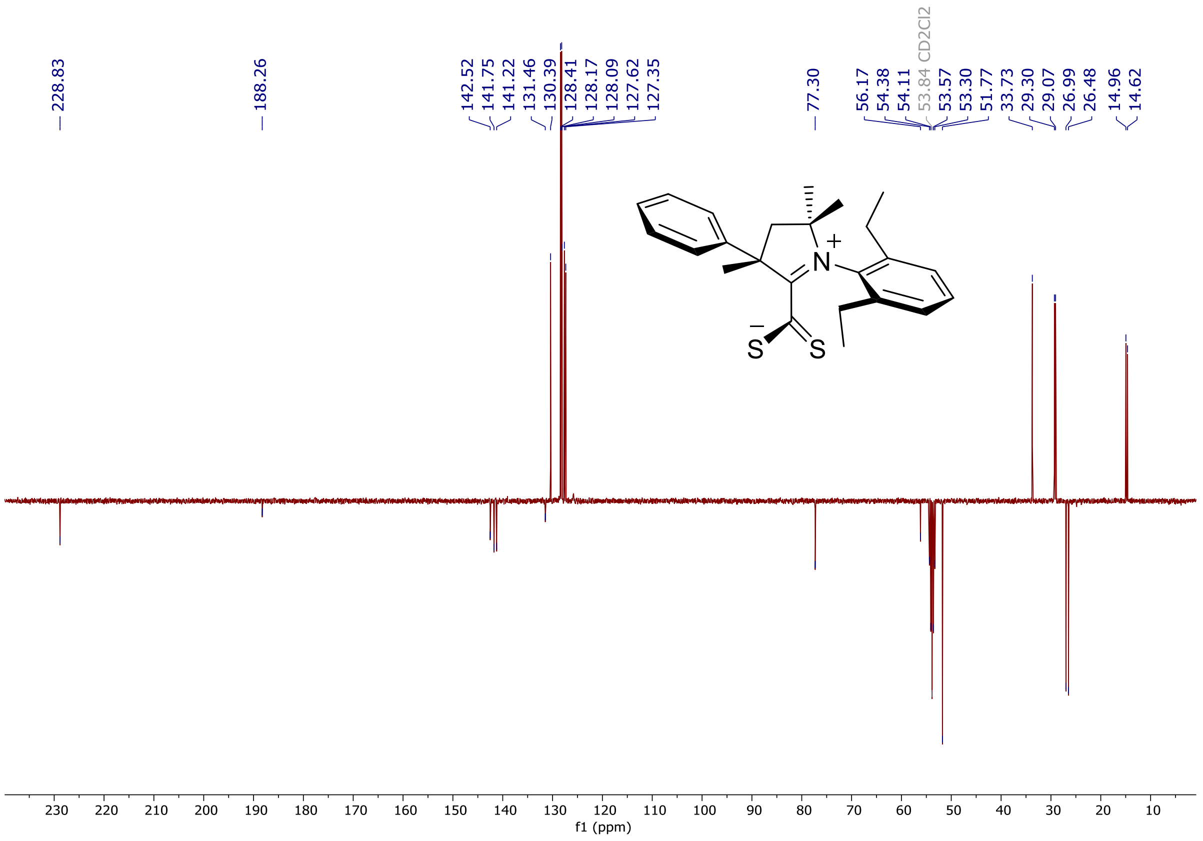

Figure S26. $^{13}C\{^{1}H\}$ APT NMR spectrum (100 MHz, CD_2Cl_2, 298 K) of 1-(2,6-diethylphenyl)-2,2,4-trimethyl-4-phenyl-3,4-dihydro-2H-pyrrol-1-ium-5-dithiocarboxylate (**4c**).

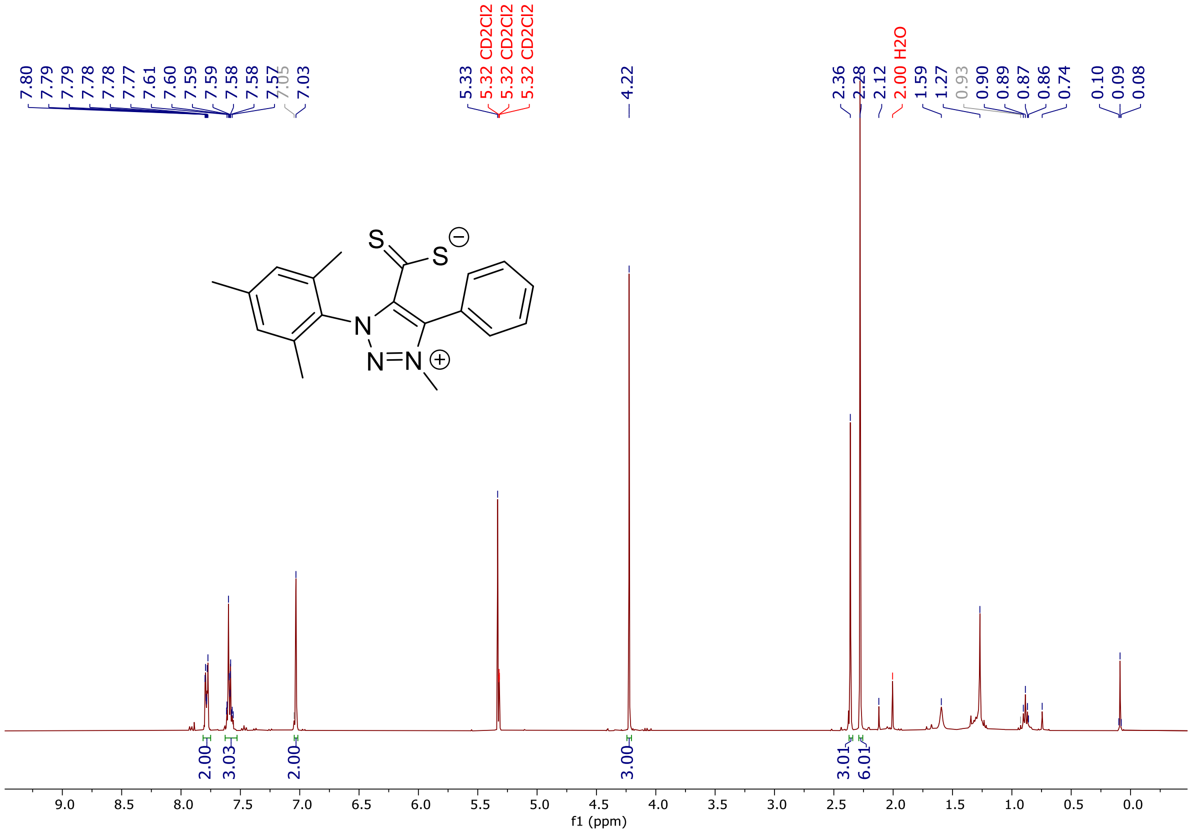

Figure S27. ^{1}H NMR spectrum (400 MHz, CD_2Cl_2, 298 K) of 1-mesityl-3-methyl-4-phenyl-1*H*-1,2,3-triazol-3-ium-5-dithiocarboxylate (**6a**).

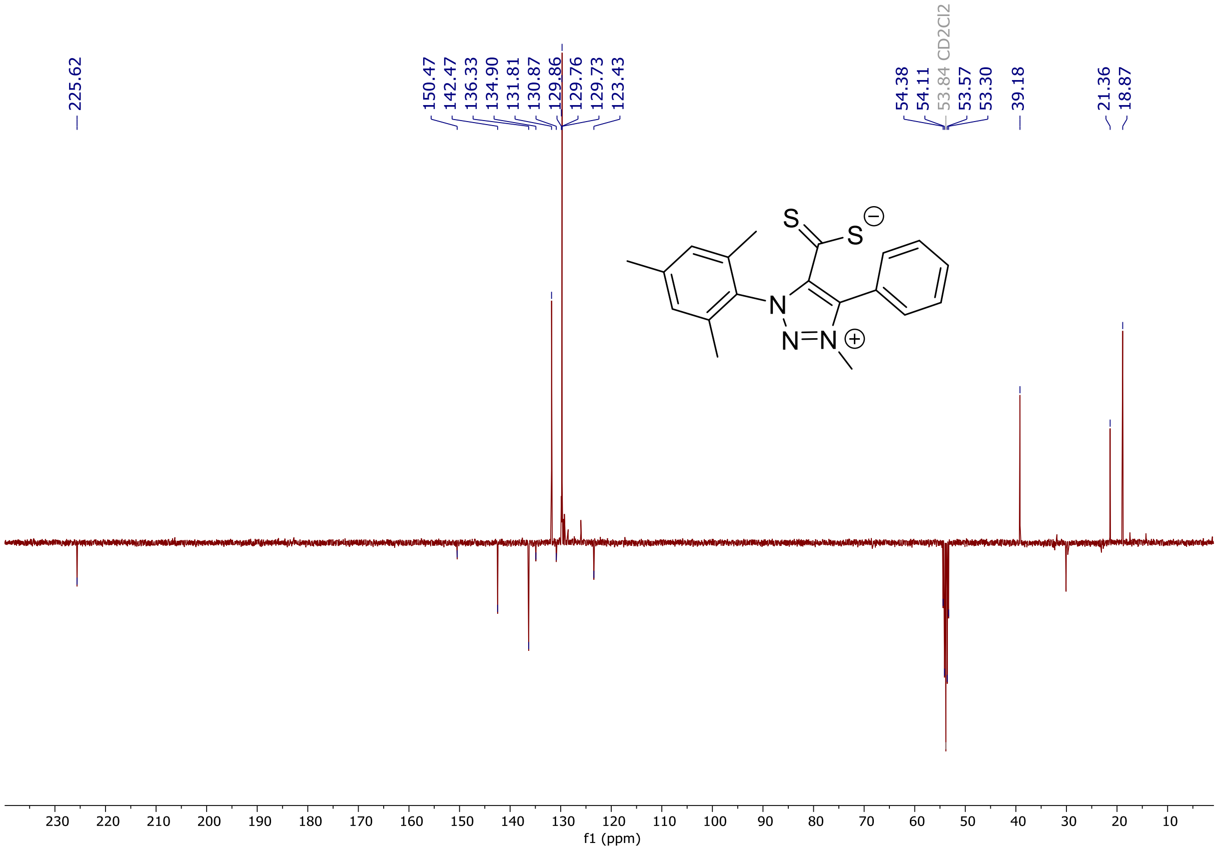

Figure S28. $^{13}C\{^{1}H\}$ APT NMR spectrum (100 MHz, CD_2Cl_2, 298 K) of 1-mesityl-3-methyl-4-phenyl-1*H*-1,2,3-triazol-3-ium-5-dithiocarboxylate (**6a**).

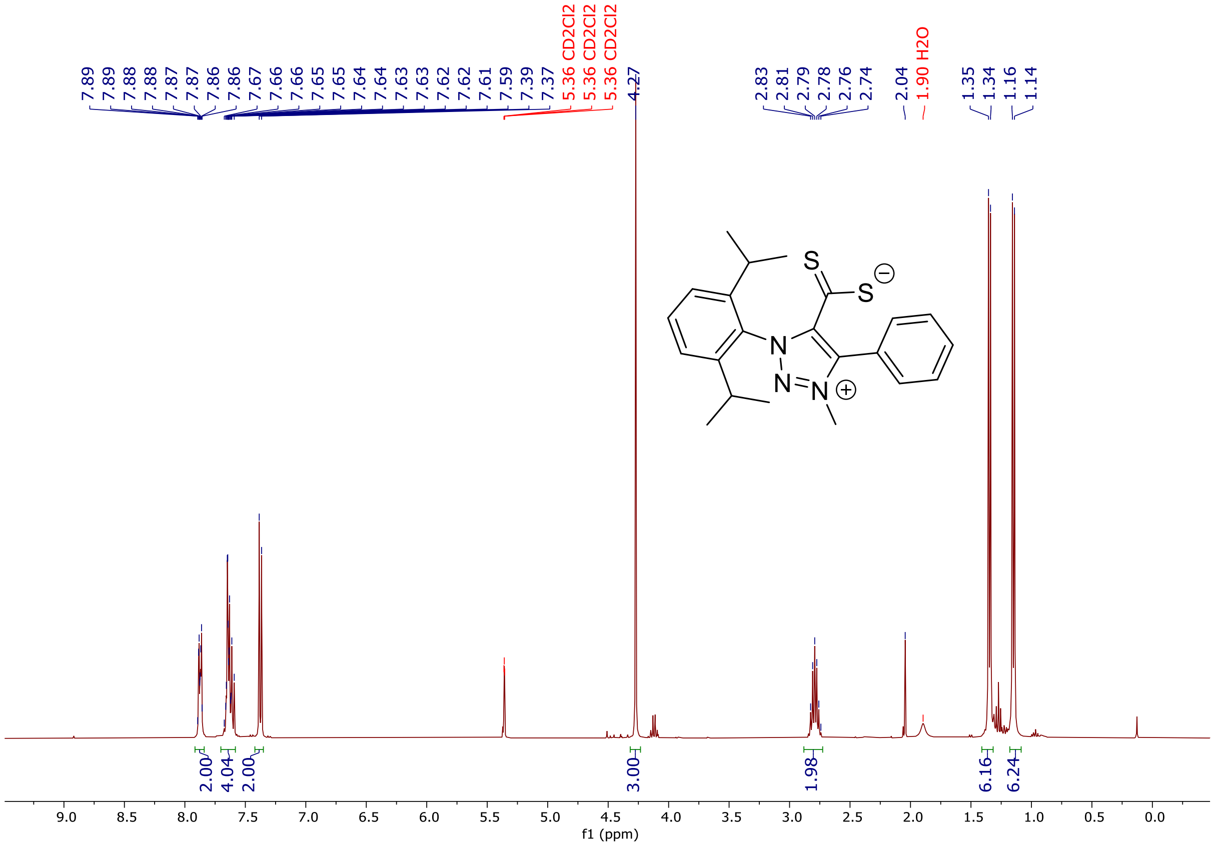

Figure S29. ^{1}H NMR spectrum (400 MHz, CD_2Cl_2, 298 K) of 1-(2,6-diisopropylphenyl)-3-methyl-4-phenyl-1*H*-1,2,3-triazol-3-ium-5-dithiocarboxylate (**6b**).

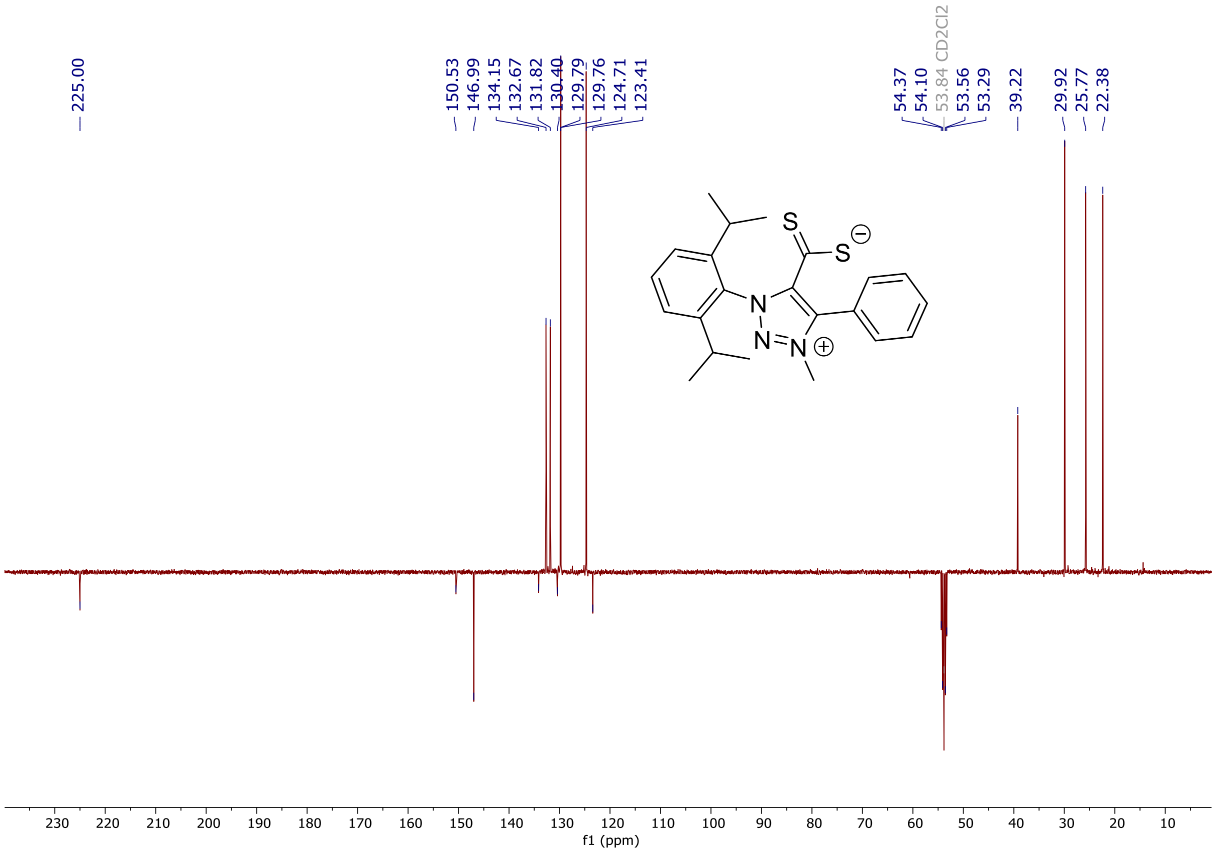

Figure S30. $^{13}C\{^{1}H\}$ NMR spectrum (100 MHz, CD_2Cl_2, 298 K) of 1-(2,6-diisopropylphenyl)-3-methyl-4-phenyl-1*H*-1,2,3-triazol-3-ium-5-dithiocarboxylate (**6b**).

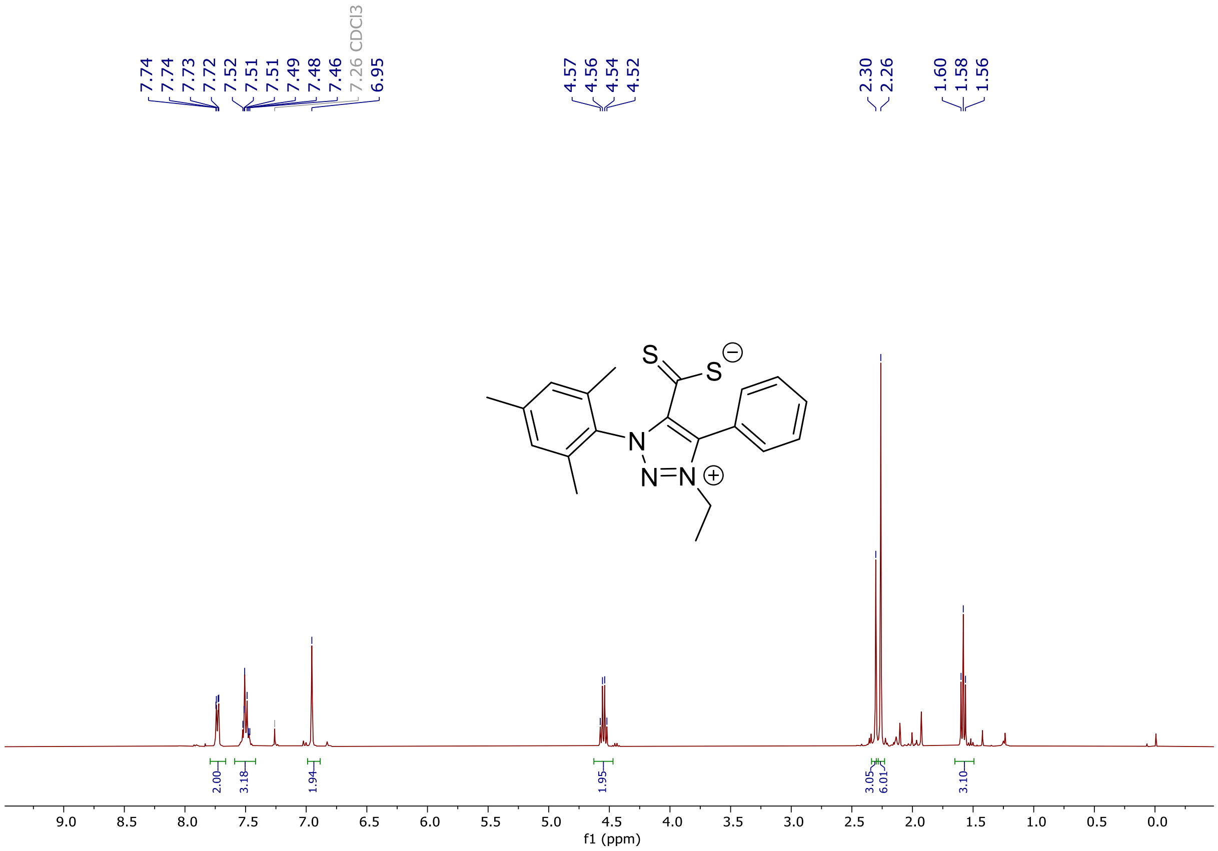

Figure S31. ^{1}H NMR spectrum (400 MHz, $CDCl_3$, 298 K) of 3-ethyl-1-mesityl-4-phenyl-1*H*-1,2,3-triazol-3-ium-5-dithiocarboxylate (**6c**).

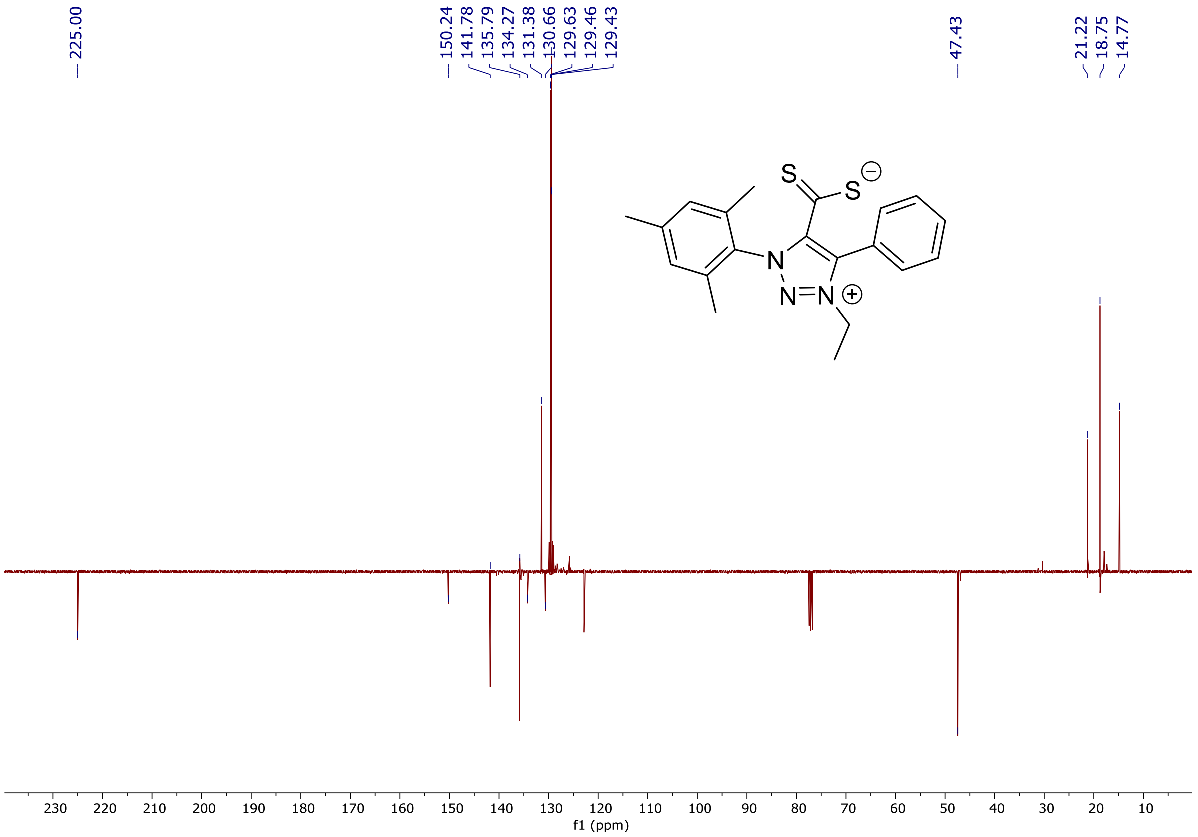

Figure S32. $^{13}C\{^{1}H\}$ APT NMR spectrum (100 MHz, $CDCl_3$, 298 K) of 3-ethyl-1-mesityl-4-phenyl-1*H*-1,2,3-triazol-3-ium-5-dithiocarboxylate (**6c**).

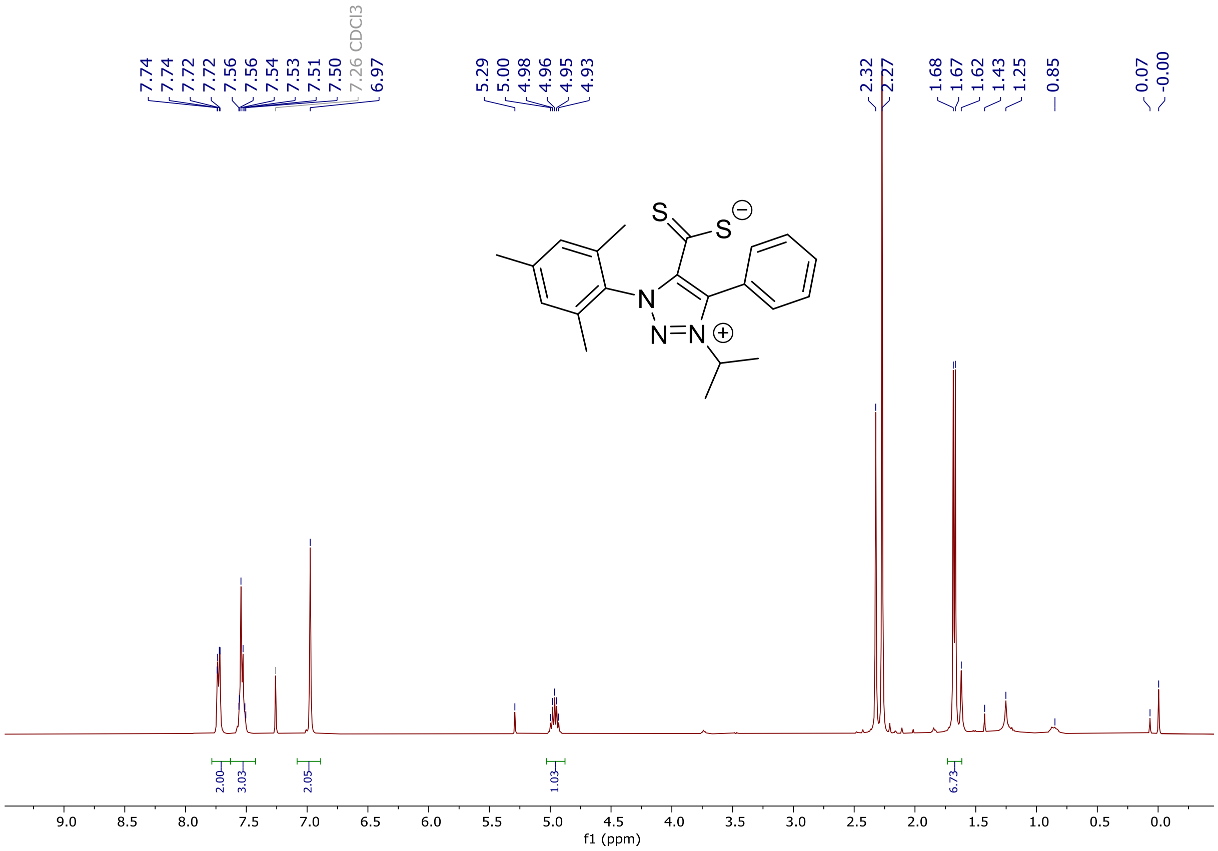

Figure S33. ^{1}H NMR spectrum (400 MHz, $CDCl_3$, 298 K) of 3-isopropyl-1-mesityl-4-phenyl-1*H*-1,2,3-triazol-3-ium-5-dithiocarboxylate (**6d**).

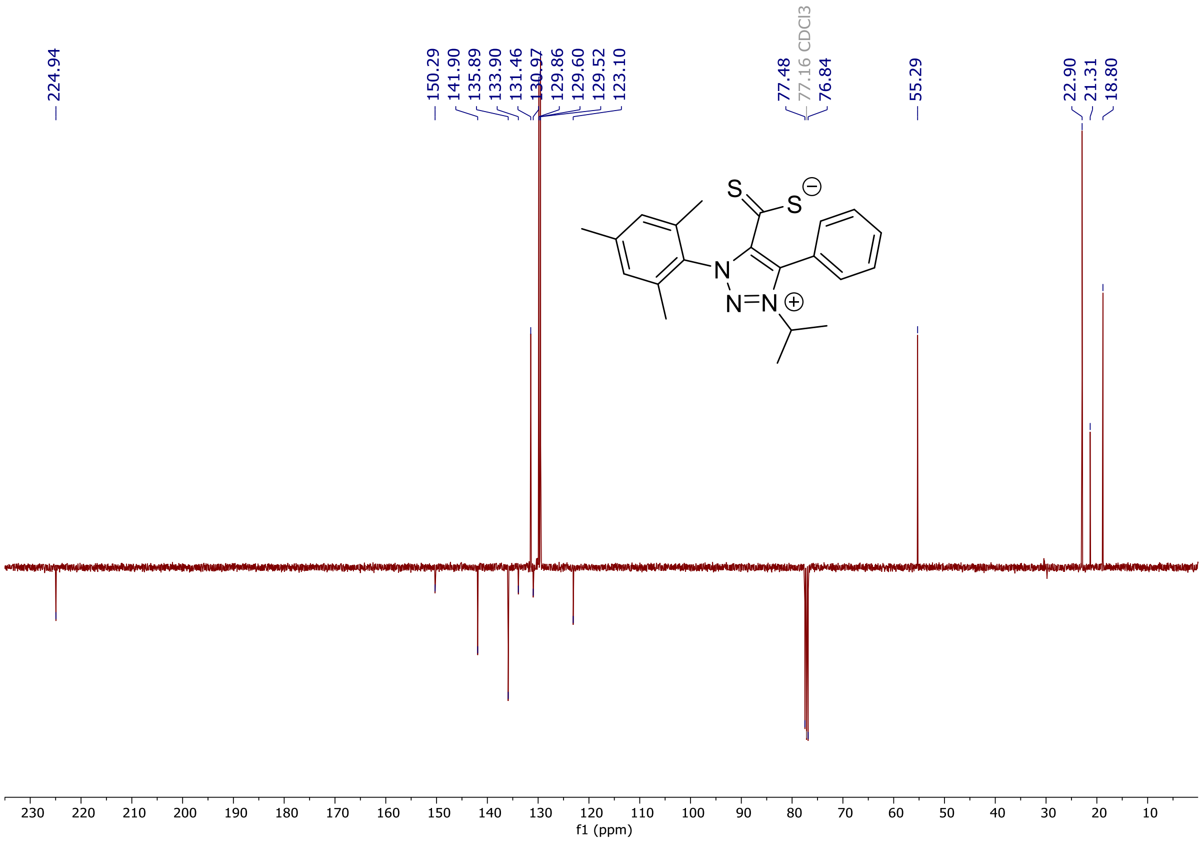

Figure S34. $^{13}C\{^{1}H\}$ APT NMR spectrum (100 MHz, $CDCl_3$, 298 K) of 3-isopropyl-1-mesityl-4-phenyl-1*H*-1,2,3-triazol-3-ium-5-dithiocarboxylate (**6d**).

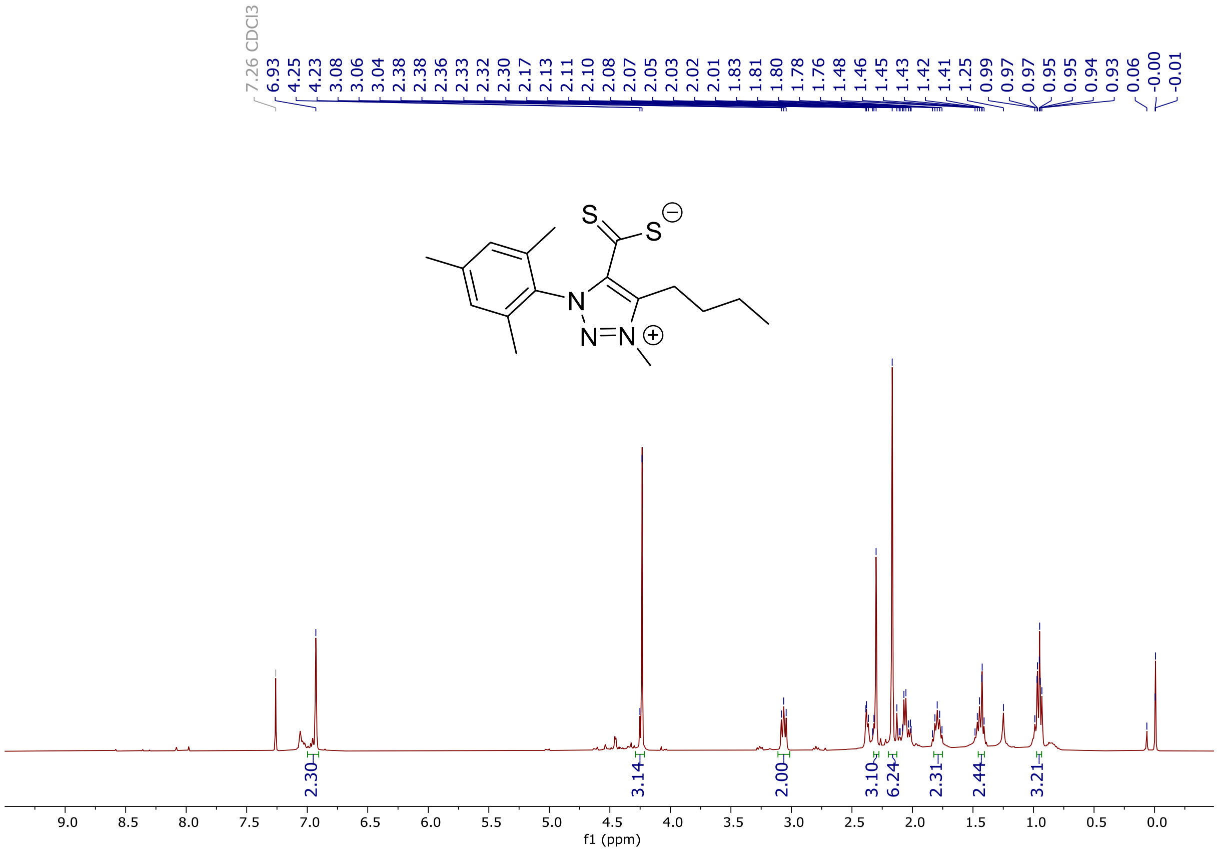

Figure S35. ^{1}H NMR spectrum (400 MHz, $CDCl_3$, 298 K) of 4-butyl-1-mesityl-3-methyl-1*H*-1,2,3-triazol-3-ium-5-dithiocarboxylate (**6e**).

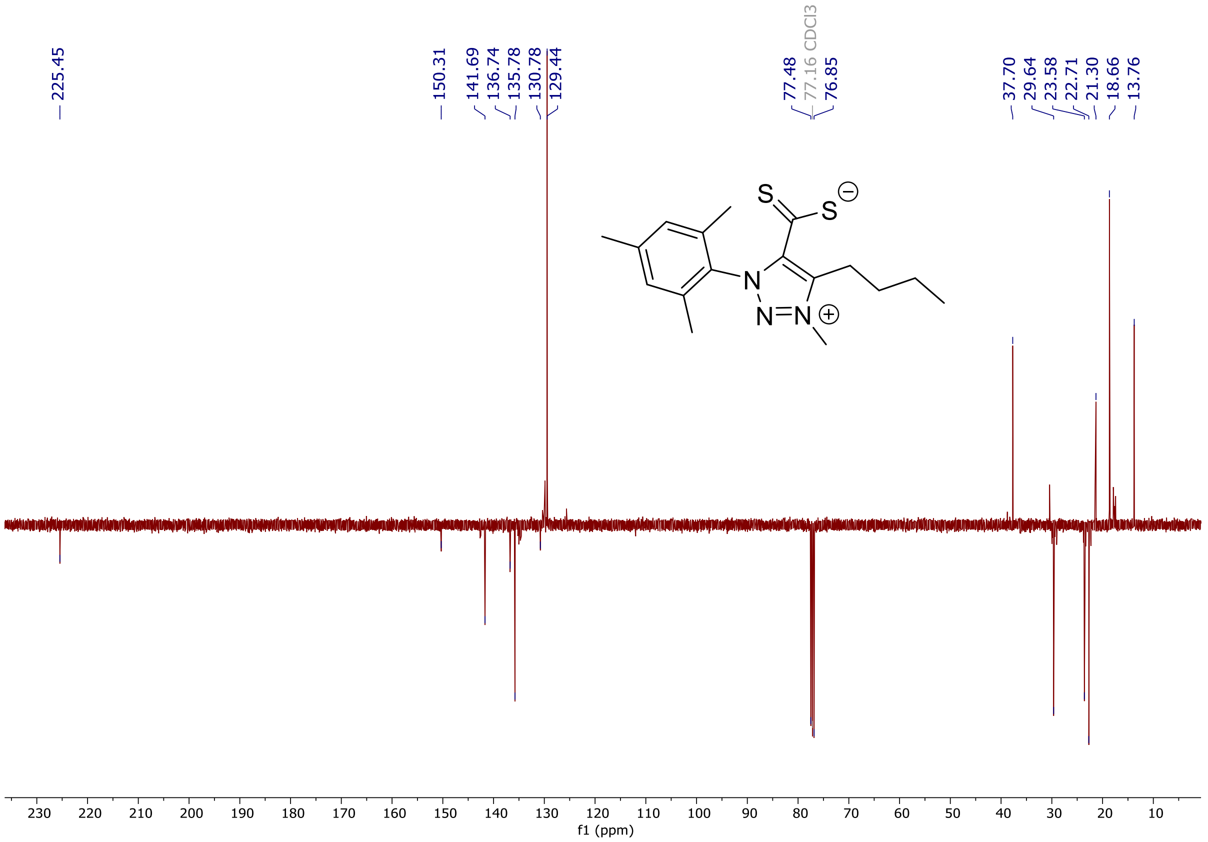

Figure S36. $^{13}C\{^{1}H\}$ APT NMR spectrum (100 MHz, $CDCl_3$, 298 K) of 4-butyl-1-mesityl-3-methyl-1*H*-1,2,3-triazol-3-ium-5-dithiocarboxylate (**6e**).

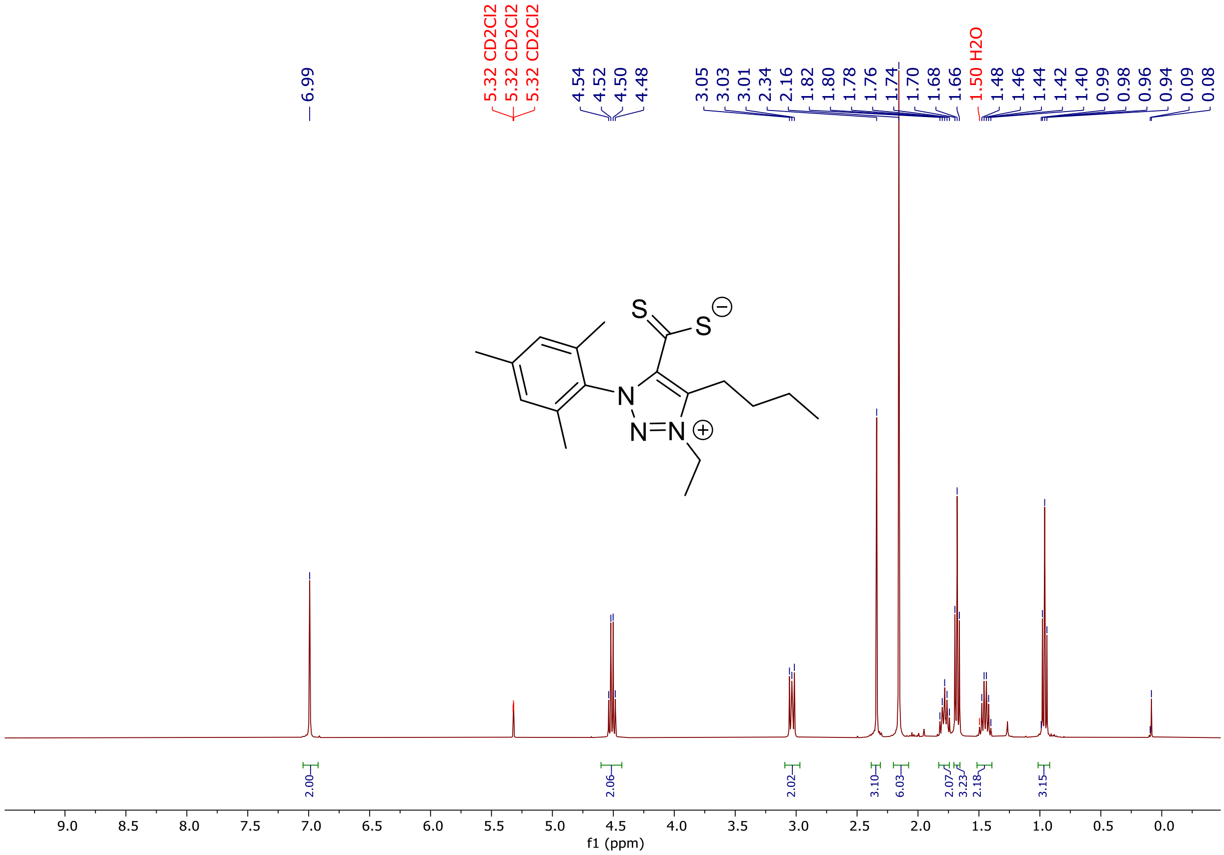

Figure S37. ^{1}H NMR spectrum (100 MHz, CD_2Cl_2, 298 K) of 4-butyl-3-ethyl-1-mesityl-1*H*-1,2,3-triazol-3-ium-5-dithiocarboxylate (**6f**).

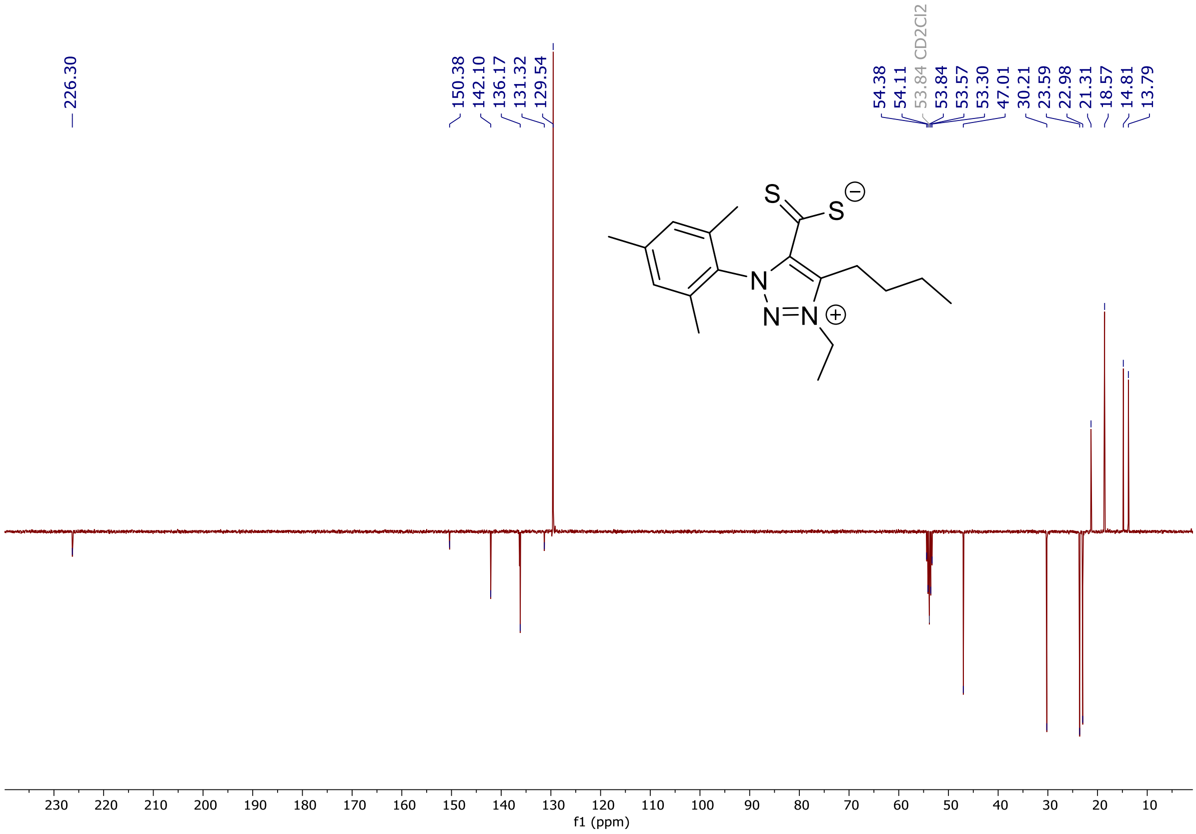

Figure S38. $^{13}C\{^{1}H\}$ APT NMR spectrum (100 MHz, CD_2Cl_2, 298 K) of 4-butyl-3-ethyl-1-mesityl-1*H*-1,2,3-triazol-3-ium-5-dithiocarboxylate (**6f**).

9. IR Spectra

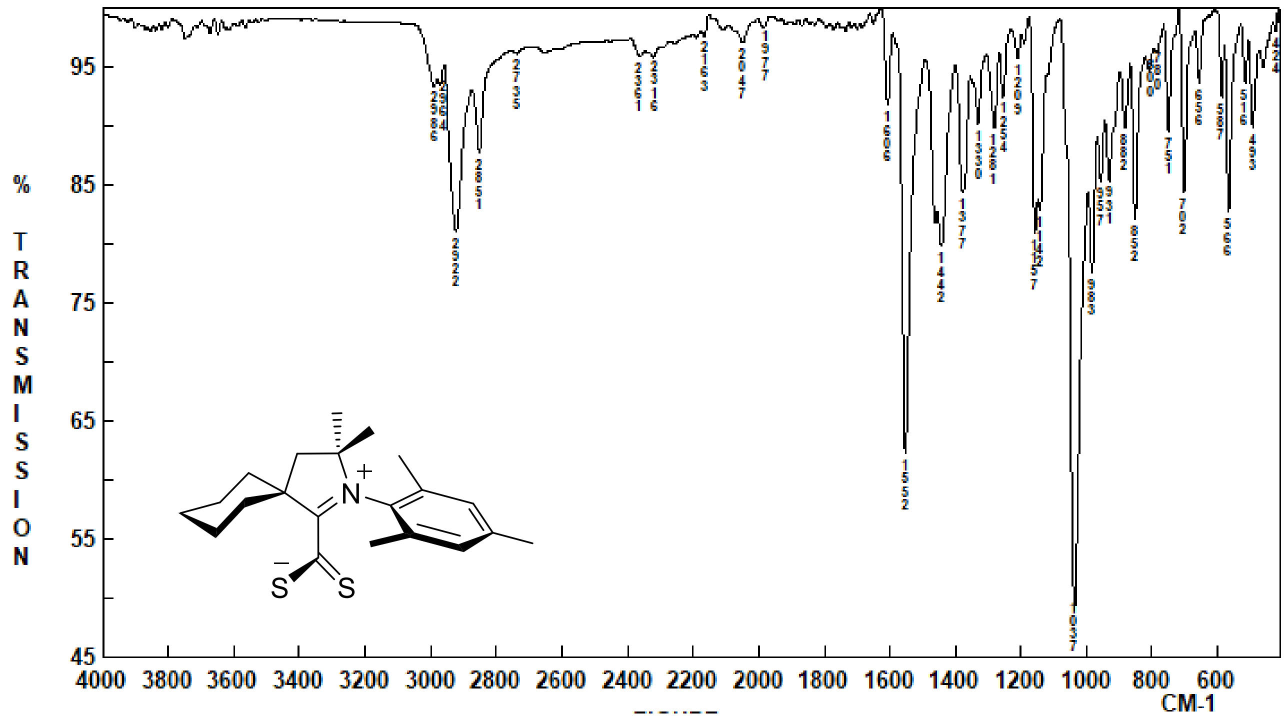

Figure S39. IR spectrum of 2-mesityl-3,3-dimethyl-2-azaspiro[4.5]dec-1-en-2-ium-1-dithiocarboxylate (**4a**).

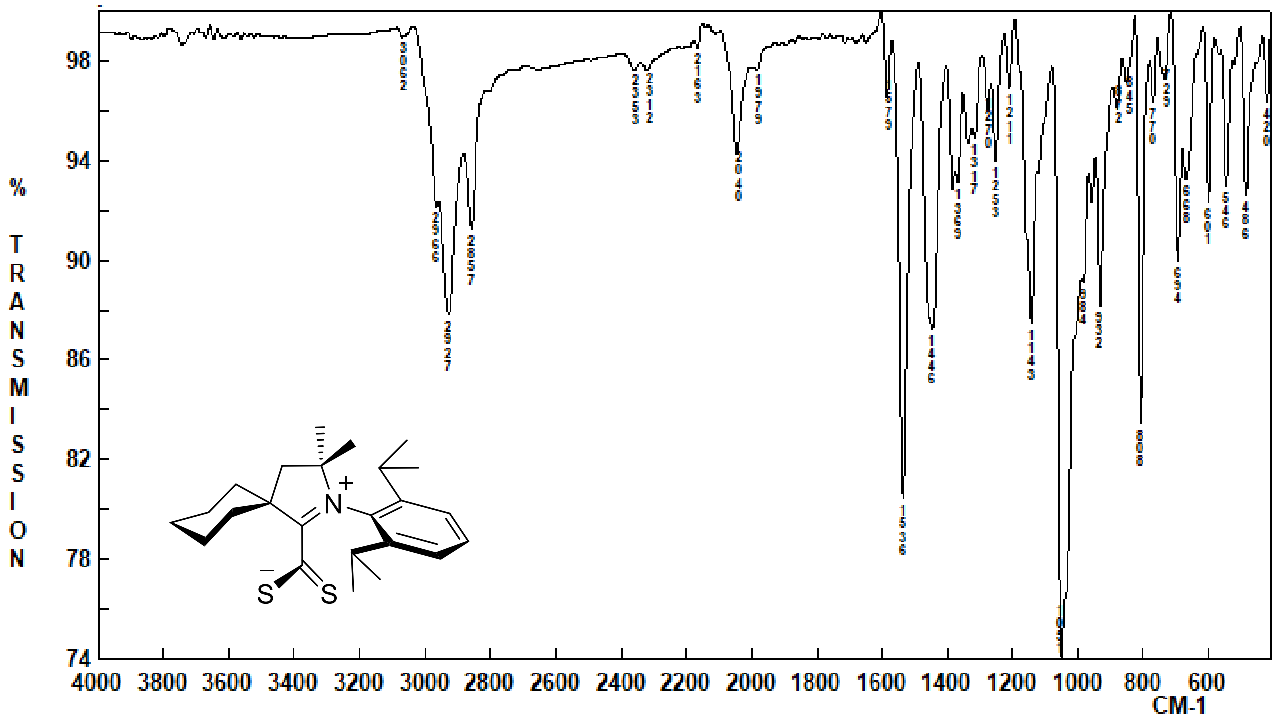

Figure S40. IR spectrum of 2-(2,6-diisopropylphenyl)-3,3-dimethyl-2-azaspiro[4.5]dec-1-en-2-ium-1-dithiocarboxylate (**4b**).

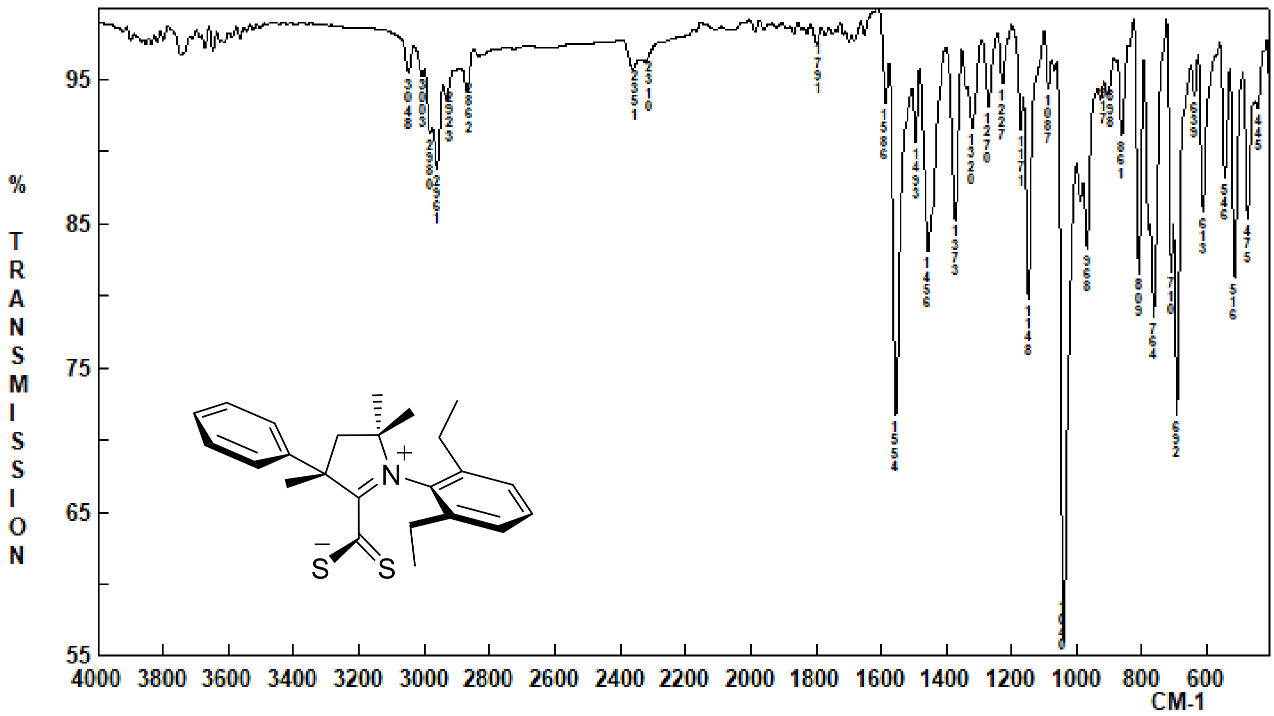

Figure S41. IR spectrum of 1-(2,6-diethylphenyl)-2,2,4-trimethyl-4-phenyl-3,4-dihydro-2H-pyrrol-1-ium-5-dithiocarboxylate (**4c**).

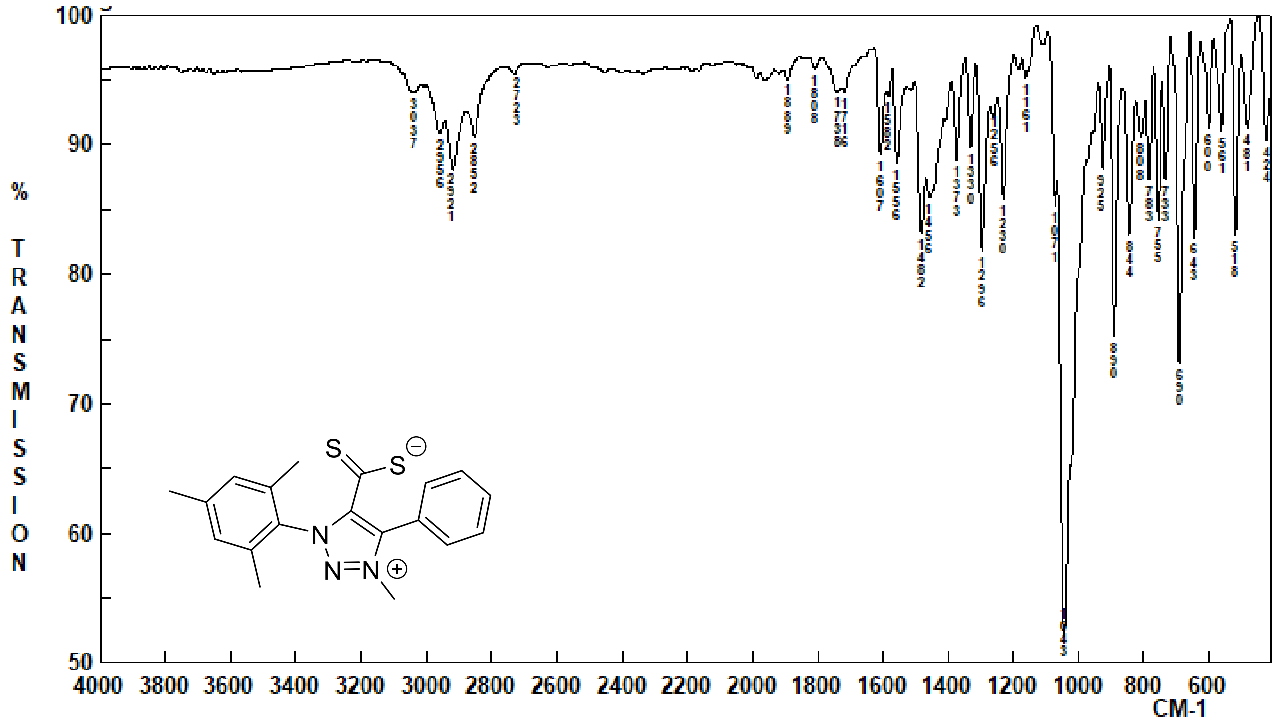

Figure S42. IR spectrum of 1-mesityl-3-methyl-4-phenyl-1*H*-1,2,3-triazol-3-ium-5-dithiocarboxylate (**6a**).

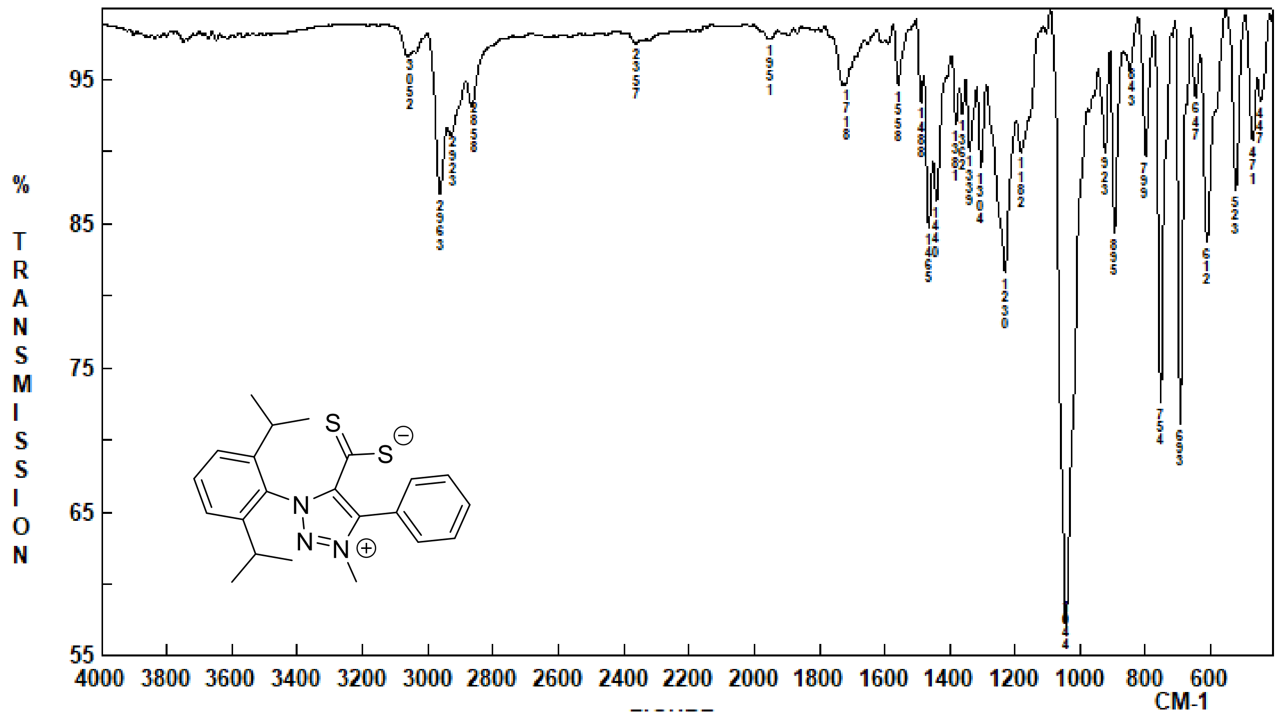

Figure S43. IR spectrum of 1-(2,6-diisopropylphenyl)-3-methyl-4-phenyl-1*H*-1,2,3-triazol-3-ium-5-dithiocarboxylate (**6b**).

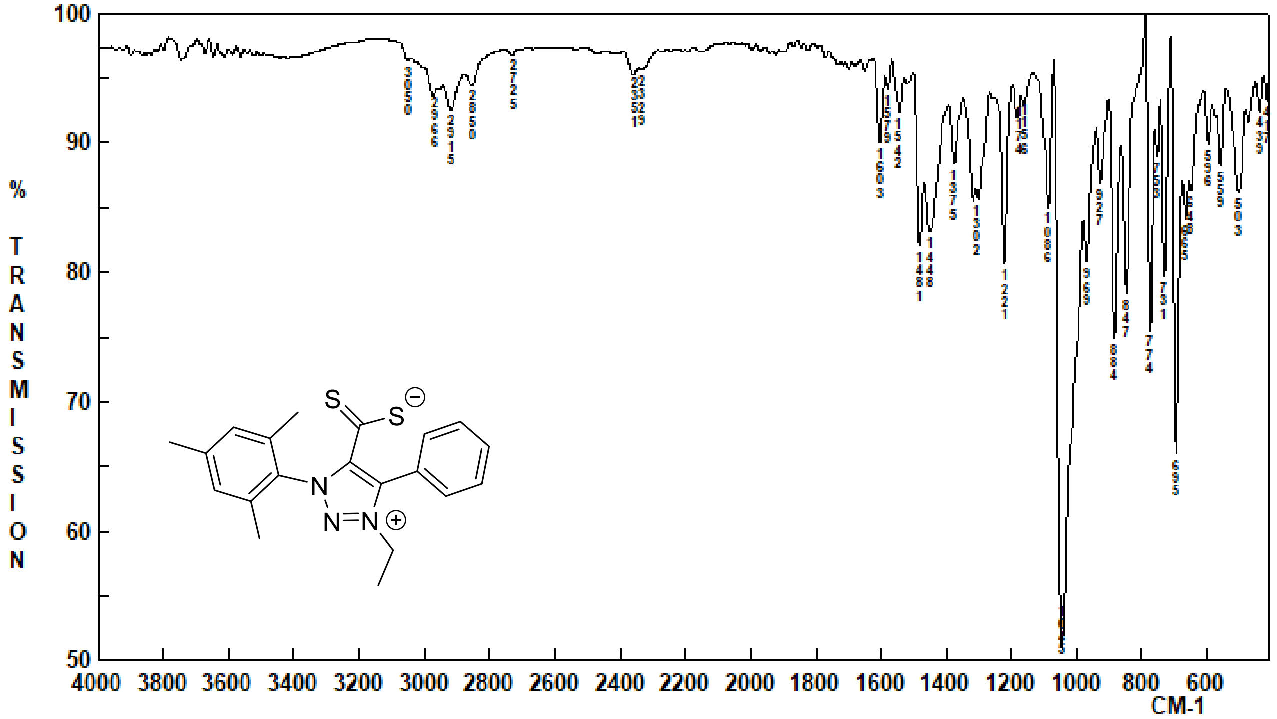

Figure S44. IR spectrum of 3-ethyl-1-mesityl-4-phenyl-1*H*-1,2,3-triazol-3-ium-5-dithiocarboxylate (**6c**).

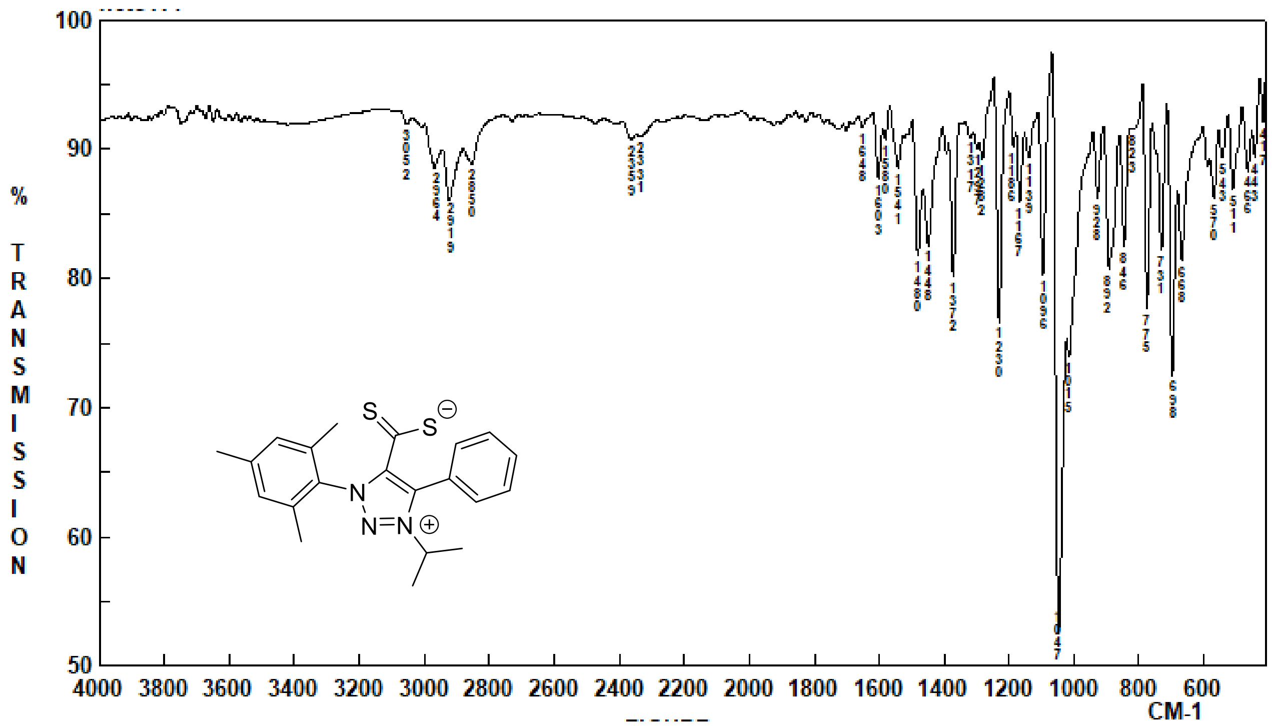

Figure S45. IR spectrum of 3-isopropyl-1-mesityl-4-phenyl-1*H*-1,2,3-triazol-3-ium-5-dithiocarboxylate (**6d**).

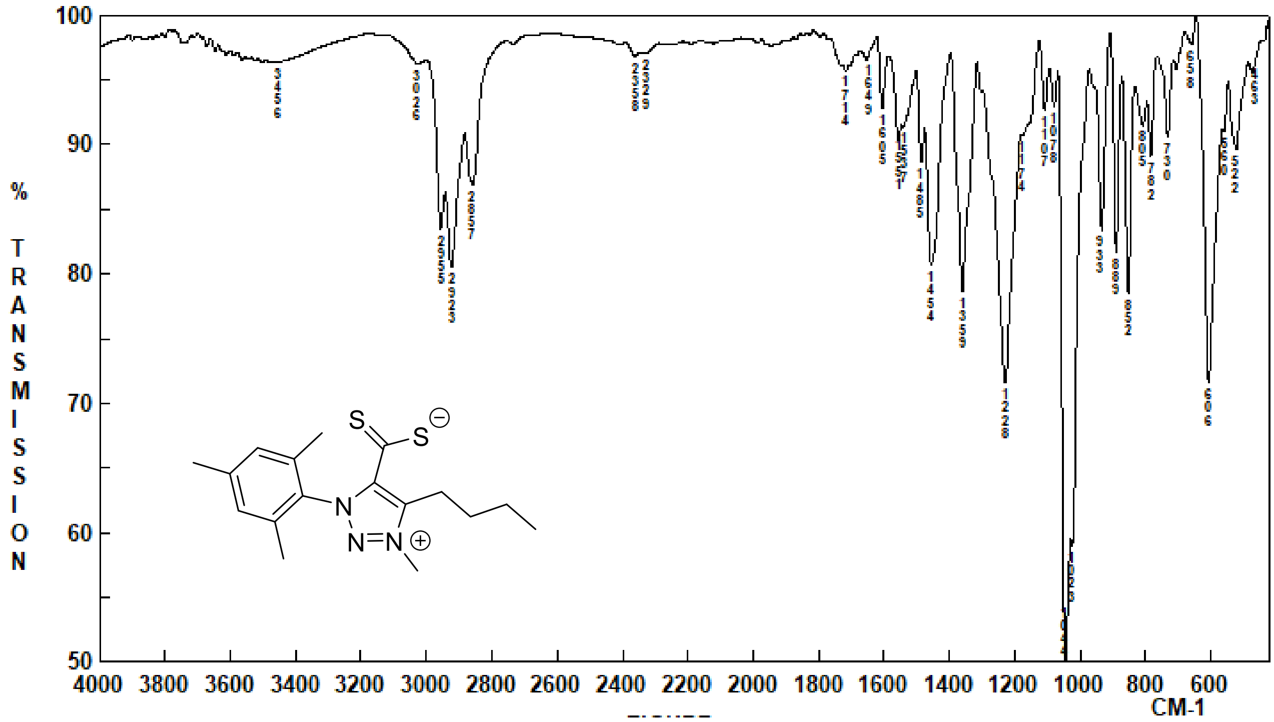

Figure S46. IR spectrum of 4-butyl-1-mesityl-3-methyl-1*H*-1,2,3-triazol-3-ium-5-dithiocarboxylate (**6e**).

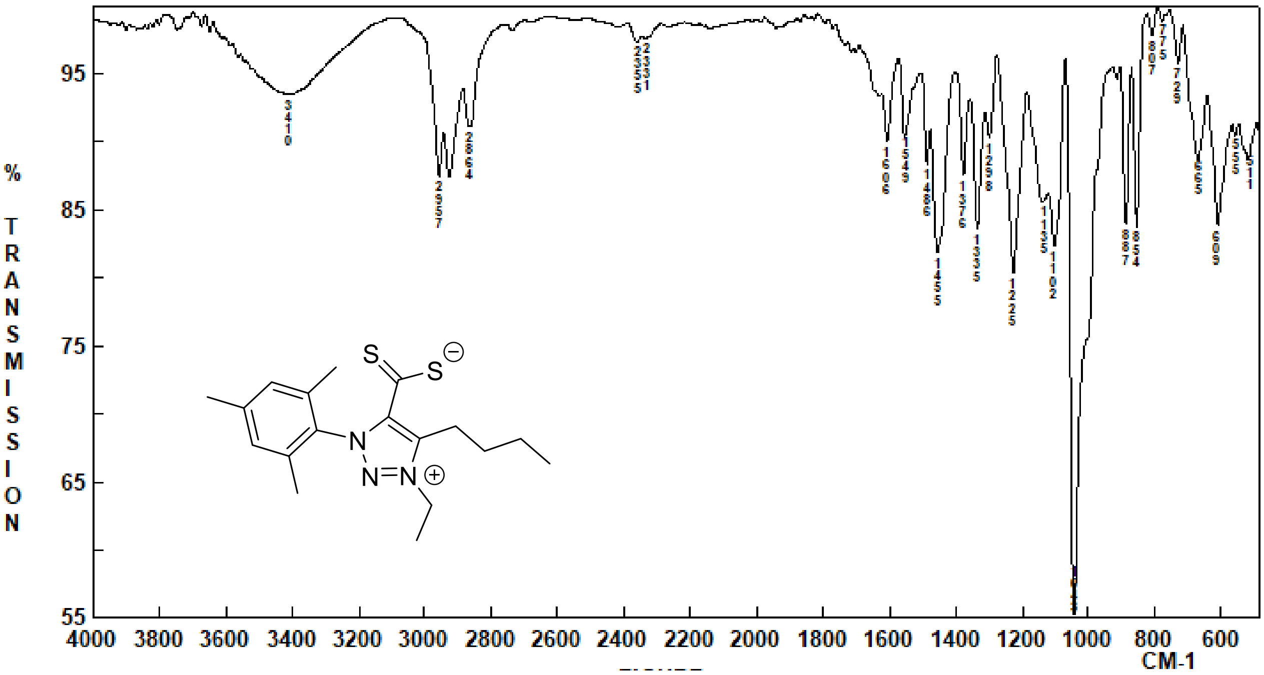

Figure S47. IR spectrum of 4-butyl-3-ethyl-1-mesityl-1*H*-1,2,3-triazol-3-ium-5-dithiocarboxylate (**6f**).
